# Chinese herbal compound preparation Qing-Xin-Jie-Yu granules for intermediate coronary lesions in patients with stable coronary artery disease: Study protocol for a multicenter, randomized, double-blind, placebo-controlled trial

**DOI:** 10.1371/journal.pone.0307074

**Published:** 2024-07-16

**Authors:** Luying Chen, Lulu Dai, Jiawei Xu, Lian Duan, Xiaoxia Hou, Lu Zhang, Libo Song, Fangfang Zhao, Yuerong Jiang

**Affiliations:** 1 National Clinical Research Center for Chinese Medicine Cardiology, Xiyuan Hospital, China Academy of Chinese Medical Sciences, Beijing, China; 2 Guang’anmen Hospital, China Academy of Chinese Medical Sciences, Beijing, China; 3 Cardiovascular Center, Beijing Tongren Hospital, Capital Medical University, Beijing, China; 4 Chinese Journal of Integrated Traditional and Western Medicine Press, Beijing, China; Saud Al-Babtain Cardiac Centre, SAUDI ARABIA

## Abstract

**Introduction:**

Despite the available secondary preventive treatments, the management of stable coronary artery disease (SCAD) remains challenging. Intermediate coronary lesion (ICL), defined as luminal stenosis between 50% and 70%, is a key stage of SCAD. However, existing therapeutic strategies are limitated in delaying plaque progression and associated with various adverse effects and economic burdens. Qing-Xin-Jie-Yu Granules (QXJYG) with proven anti-platelet, anti-inflammatory, and lipid-lowering effects may compensate for the drawbacks of current treatments and can be tested as a complementary therapy. Therefore, this study aims to investigate the efficacy and safety of QXJYG in treating ICL, with a particular focus on its impact on myocardial ischemia and plaque progression.

**Materials and methods:**

This is a multicenter, randomized, double-blind, placebo-controlled trial. A total of 120 participants with ICL will be randomly assigned to two groups in a 1:1 ratio. In addition to basic medications, the intervention group will receive QXJYG, while the control group will receive a placebo for over 6 months, followed by a 12-month follow-up. The primary efficacy outcome is computed tomography-derived fractional flow reserve. The secondary outcomes include the degree of coronary stenosis, coronary artery calcification score, Gensini score, Seattle Angina Questionnaire score, high-sensitivity C-reactive protein, matrix metalloproteinase-9, blood lipids, and carotid artery ultrasound parameters. Major adverse cardiovascular events are recorded as endpoints. The safety outcomes include composite events of bleeding, laboratory test results, and adverse events. Clinical visits are scheduled at baseline, every 2 months during the treatment, and after a 12-month follow-up.

**Discussion:**

This trial is anticipated to yield reliable results to verify the efficacy and safety of QXJYG in the treatment of ICL, which will provide novel insights to help address the prevailing therapeutic dilemma of ICL, thereby facilitating for the management of SCAD.

**Trial registration:**

Chinese Clinical Trial Registry, ChiCTR2200059262. Registered on April 27, 2022.

## Introduction

Coronary artery disease (CAD) is a leading cause of mortality globally, with its incidence increasing every year [1, 2]. Stable coronary heart disease (SCAD) has a higher incidence than myocardial infarction (MI) [3]. Despite the available secondary preventive treatment, managing the progression of SCAD remains challenging. Intermediate coronary lesion (ICL), defined as luminal diameter stenosis between 50% and 70% [4], is a key stage in the progression of SCAD. Approximately 78% MIs originate from ICL, which are often precipitated by plaque rupture [5]. However, in patients with SCAD, ICL resides within a gray area of revascularization [6], and drug therapy remains the mainstay treatment. Anti-platelet therapy, such as aspirin, can prevent ischemic adverse events but entails a risk of bleeding [7]. Intensive lipid-lowering therapy has demonstrated the potential to reduce the occurence of cardiovascular events [8]. However, statin intolerance is an ongoing problem, and using proprotein convertase subtilisin/kexin type 9 (PCSK-9) inhibitors is costly. A residual inflammatory risk also persists, leading to the onset of cardiovascular events [9]. The available anti-inflammatory agents are marked by inconclusive evidence and burdened by serious side effects [10, 11]. Moreover, the patient’s quality of life and mental well-being are negatively affected by recurrent angina, which raises an additional concern.

Qing-Xin-Jie-Yu Granules (QXJYG), a Chinese herbal preparation composed of *Astragalus membranaceus* (Huangqi), *Salvia miltiorrhiza Bunge* (Danshen), *Ligusticum chuanxiong Hort* (Chuanxiong), *Pogostemon cablin* (Guanghuoxiang), and *Coptis chinensis* (Huanglian), has been used to treat CAD for nearly two decades in China. QXJYG can alleviate angina and reduce the incidence of cardiovascular events [12–14], possibly due to its regulatory effects on glycolipid metabolism and anti-platelet and anti-inflammatory properties [15]. A previous animal study has affirmed the ability of QXJYG to lower lipid levels and counteract high-fat diet-induced inflammation through the modulation of the gut microbiota and bile acid metabolism. [16]. It also impedes the onset of atherosclerosis by inhibiting macrophage ferroptosis [17]. However, the impact of QXJYG on delaying atherosclerosis progression, thereby improving myocardial ischemia, necessitates substantiation through high-quality evidence from clinical trials. Therefore, this study designed as a multicenter, randomized, double-blind, placebo-controlled clinical trial aims to validate the efficacy and safety of QXJYG in treating ICL.

## Materials and methods

### Study objective

This exploratory study primarily aims to evaluate the efficacy and safety of QXJYG in treating ICL. It hypothesizes that QXJYG combined with conventional drug therapy will demonstrate superior outcomes when compared with conventional drug therapy alone.

### Study design

**Fig 1:**
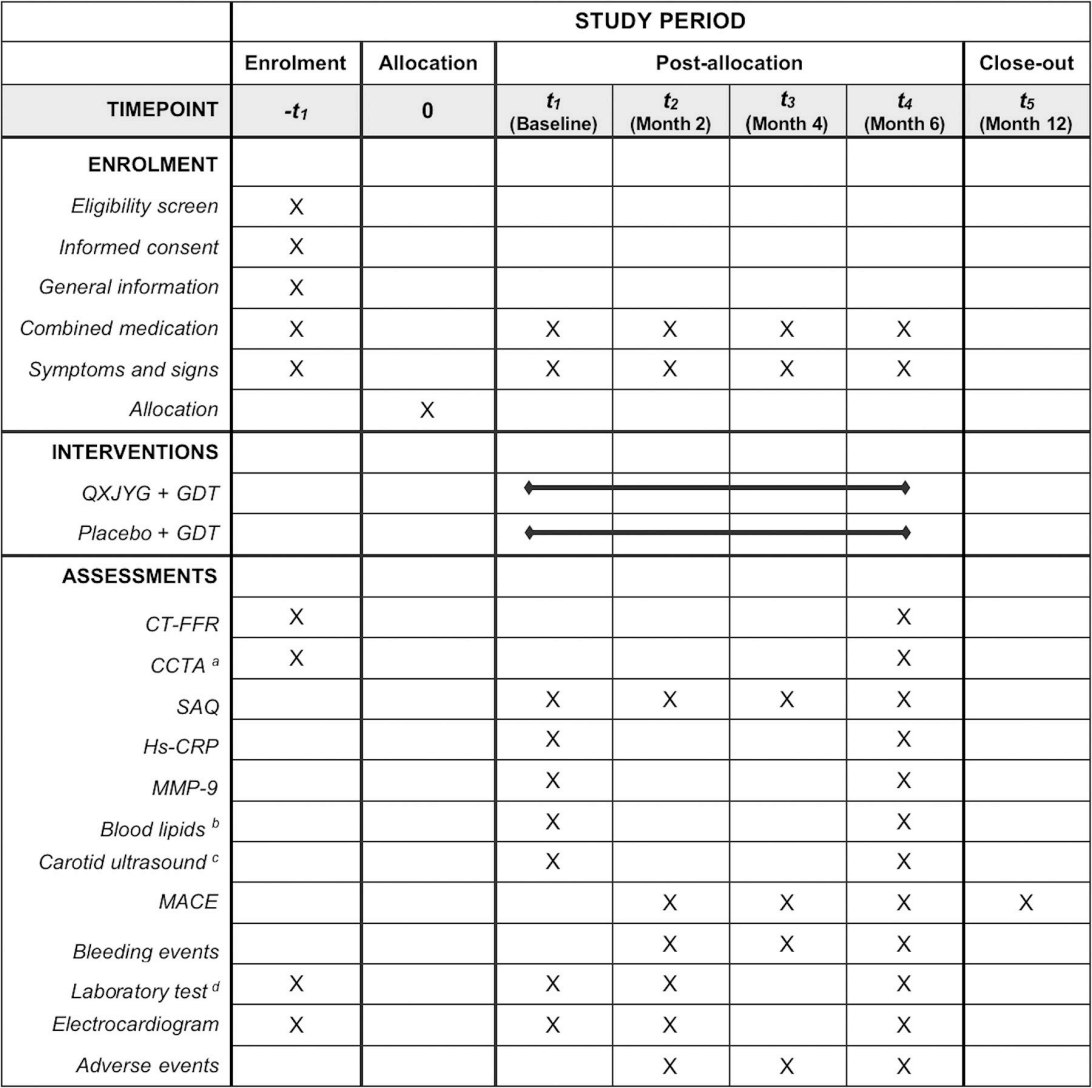
A SPIRIT schedule of study enrollment, interventions, and assessments. QXJYG: Qing-Xin-Jie-Yu Granules; GDT: Guideline-directed drug therapy; CT-FFR: Computed tomography-derived fractional flow reserve; CCTA: Coronary computed tomography angiography; SAQ: Seattle Angina Questionnaire; Hs-CRP: High-sensitivity C-reactive protein; MMP-9: Matrix metalloproteinase-9; MACE: Major adverse cardiovascular events. **Note:** ^a^ CCTA includes the percentage of diameter and area stenosis, coronary artery calcification score, and Gensini score derived from the CT images. ^b^ Blood lipids include total cholesterol (TC), triglycerides (TG), low-density lipoprotein cholesterol (LDL-C), high-density lipoprotein cholesterol (HDL-C), apolipoprotein A1 (Apo A1), Apolipoprotein B (Apo B) and lipoprotein (a) [Lp(a)]. ^c^ Carotid ultrasound parameters include IMT (mm), carotid plaque length × thickness (mm), and carotid lumen stenosis (%). ^d^ Laboratory tests include complete blood count, coagulation function, liver and renal function, blood glucose, and routine urine and stool examinations.

**Fig 2:**
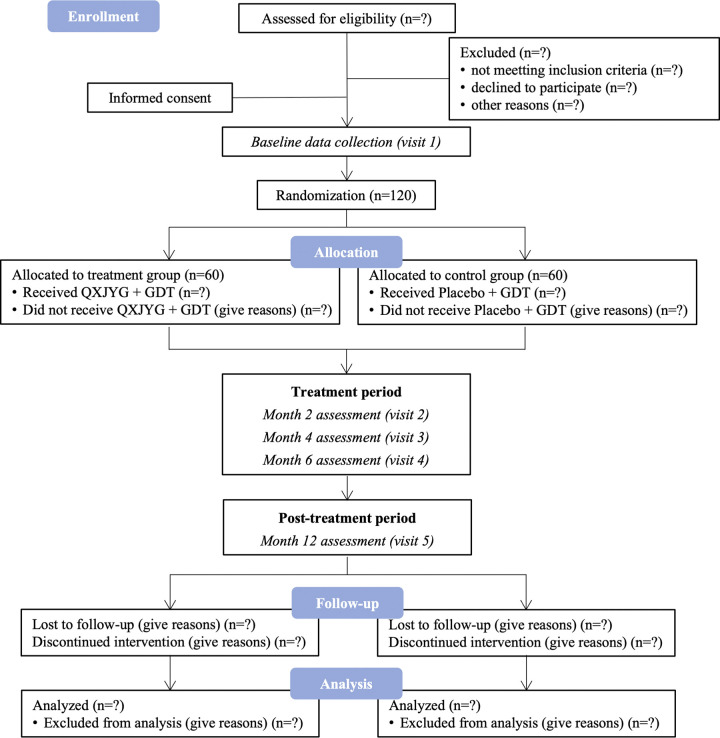
Study flow diagram. QXJYG: Qing-Xin-Jie-Yu Granules; GDT: Guideline-directed drug therapy.

This multicenter, block-randomized, double-blind, placebo-controlled trial has been registered in the Chinese Clinical Trial Registry (registration number: ChiCTR2200059262), and the protocol has been approved by the Ethics Committee of Xiyuan Hospital, China Academy of Chinese Medical Sciences (reference number: 2022XLA038-1) (**S1 and S2 Files**). This trial is expected to run from June 2022 to June 2025, following the principles of the Declaration of Helsinki and the Good Clinical Practice guidelines. Patients who meet the eligibility criteria will be recruited from three centers in China after signing a written informed consent form (ICF). Subsequently, 120 participants will be randomly assigned to the QXJYG group (n = 60) or the placebo group (n = 60) for 6-month treatment and total 12-month follow-up. Clinical visits are scheduled at baseline (visit 1), every 2 months during the treatment (visits 2–4), and after the 12-month follow-up (visit 5). Data management and statistical analysis will be conducted independently by third-party personnel stationed at the Clinical Pharmacology Center, Xiyuan Hospital, China Academy of Chinese Medicine Sciences. This protocol strictly adheres to the international recommendations of SPIRIT 2013 statements (**S3 File**) [18], and a detailed schedule is provided in **Fig 1**. When reporting the study results, compliance with the Consolidated Standards of Reporting Trials (CONSORT 2017) [19] will be ensured, as depicted in the flow diagram presented in **Fig 2**. The study design can be summarized as shown in **S1 Fig**.

### Participants

#### Diagnostic criteria

The “2021 ACC/AHA/SCAI Guideline for Coronary Artery Revascularization” will be followed as the diagnosis criteria for ICL [6]; ICL is defined as luminal diameter stenosis ranging from 50% to 70% in major coronary arteries. Only patients diagnosed with SCAD will be screened. The diagnosis criteria of SCAD will be followed as the “2014 ACC/AHA/AATS/PCNA/SCAI/STS focused update of the guideline for the diagnosis and management of patients with stable ischemic heart disease” [20] and the “2021 AHA/ACC/ASE/CHEST/SAEM/SCCT/SCMR Guideline for the Evaluation and Diagnosis of Chest Pain” [21].

#### Inclusion criteria

1. Patients with SCAD who have at least one major coronary artery with a luminal diameter stenosis of 50–70%, as confirmed using coronary computed tomography angiography (CCTA).
2. Patients with heart function graded as NYHA grade Ⅰ–Ⅱ.
3. Patients aged 18–75 years.
4. Patients who are well-informed and voluntarily sign the ICF.

#### Exclusion criteria

1. Patients who underwent coronary stent implantation or coronary artery bypass grafting, or those with a history of MI within the last 3 months.
2. Lesions characterized by diameter stenosis ≥50% in the left main coronary artery or in all three major arteries including the left anterior descending, left circumflex, and right coronary arteries.
3. Presence of diffuse lesions throughout the diseased vessels.
4. Severe calcified lesions with a coronary artery calcification score (CACS) ≥1000.
5. History of severe blood pressure fluctuations or poorly controlled hypertension, with systolic blood pressure ≥160 mmHg and diastolic blood pressure ≥100 mmHg within the last 3 months.
6. Resting heart rate >100 beats/min that is difficult to control.
7. Patients with a history of other cardiac operations, such as valve replacement.
8. Patients with severe cardiac, hepatic, or renal insufficiency rendering them unsuitable for CCTA and related examinations and treatments.
9. Patients with mental disorders.
10. Patients afflicted by hepatitis, tuberculosis, acquired immunodeficiency syndrome, or other infectious diseases.
11. Patients with allergic predispositions.
12. Pregnant and lactating women
13. Anticipated life expectancy ≤1 year.
14. Participation in other clinical trials within the last 3 months.

### Recruitment

The recruitment period is from June 2022 to December 2024 and will encompass both inpatients and outpatients across the three research centers (**Table 1**). Various recruitment methods will be employed, including direct identification during routine clinical processes, public notifications through advertising, and screening via hospital’s internal database. Each potentially qualified patient will undergo a thorough evaluation by two experienced cardiologists independently, to ascertain whether they meet the recruitment criteria. Prior to enrollment, each participant will be given a comprehensive introduction outlining the trial’s purpose, procedures, potential benefits, associated risks, and rights they possess and will be required to voluntarily sign a written ICF.

**Table 1:** Hospitals participating in this study.

Code	Participating Hospitals	Role
**01**	Xiyuan Hospital of China Academy of Chinese Medical Sciences, Beijing	Leader
**02**	Guan’anmen Hospital of China Academy of Chinese Medical Sciences, Beijing	Member
**03**	Beijing Tongren Hospital Affiliated to Capital Medical University, Beijing	Member

### Interventions

#### Intervention medications

According to the existing guidelines [22, 23], patients diagnosed with SCAD will be administered anti-platelet and lipid-lowering drugs as the basic treatment. The details of the basic treatment protocol and the drug information are shown in **Tables 2 and 3**. Other guideline-directed medications for MI prevention, symptom relief, and risk factor management will also be used at the patient’s discretion. Notably, anti-ischemic medications (including nitrates, beta-blockers, and calcium channel blockers) may affect the accuracy of computed tomography-derived fractional flow reserve (CT-FFR) assessment, while PCSK-9 inhibitors used for more than a year can significantly reduce the plaque volume [24–26]. Therefore, the regimens and dosages of the patients taking these medications before enrollment will be maintained, whereas such medications will not be initiated during the study in those who did not take them previously. Medications that are necessary for the patients’ concurrent conditions and do not impact the interpretation of outcomes will be permissible. All therapies used during the trial will be accurately documented, including the name, dosage, frequency, and duration of use, to optimize the statistical analysis and reporting of the effectiveness of the basic treatment. Meanwhile, lifestyle modifications will be recommended, such as smoking cessation, exercise, and diet modification [27].

**Table 2:** Basic treatment protocol.

Patients with a primary diagnosis of CAD	
Anti-platelet drugs	Aspirin 100 mg will be administrated daily.
	Clopidogrel 75 mg daily will be recommended as an alternative for patients with aspirin intolerance.
Lipid-lowering drugs	Atorvastatin 20 mg will be administrated daily.
Patients with a long-standing diagnosis of CAD	
Anti-platelet drugs	Aspirin 100 mg, or clopidogrel 75 mg, according to the original dosing regimen
Lipid-lowering drugs	For patients who achieved the goal*, the original lipid-lowering regimen will be continued.
	For patients who did not achieve the goal*, the original lipid-lowering regimen will be enhanced by adding ezetimibe 10mg daily.

*Lipid-lowering goals: LDL-C <1.8 mmol/L for patients with CAD, and LDL <1.4 mmol/L for CAD patients with diabetes. CAD: coronary artery disease; LDL-C: low-density lipoprotein cholesterol; HDL-C: high-density lipoprotein cholesterol.

**Table 3:** Information on the basic medications.

Medicine name	Brand name	Specifications	Manufacturer
Aspirin enteric-coated tablets	Bayaspirin^®^	100 mg/tablet	Bayer HealthCare Manufacturing S.r.l.
Clopidogrel bisulfate tablets	PLAVIX^®^	75 mg/tablet	Sanofi Winthrop Industrie
Atorvastatin calcium tablets	Lipitor®	20 mg/tablet	Pfizer Ireland Pharmaceuticals
Ezetimibe tablets	Crestor^®^	10 mg/tablet	MSD International Gmbh (Singapore Branch)

Based on the conventional therapy, participants will be randomly assigned to either the intervention group treated with QXJYG or the control group treated with a placebo for a continuous 6-month treatment. Both groups will receive 12-month follow-up. The test drugs, QXJYG and its placebo granules, are manufactured by China Resources Sanjiu Modern Chinese Medicine Pharmaceutical Co., Ltd. QXJYG consists of five herbal granules (**Table 4)**. The placebo is composed of 5% raw materials of QXJYG and 95% dextrin, mimicking the appearance, smell, and taste of the active granules. The drug production strictly complies with the Good Manufacturing Practice guidelines, and the drug quality conforms the National Drug Standards issued by the National Medical Products Administration. The details of drug quality inspections are provided in **S4 File**.

**Table 4:** Herbs and active components in QXJYG.

Chinese name	Latin or English name	Origin	Active components [15]	Weight (%) *
**Huang Qi**	*Astragalus membranaceus*	The dried root of *Astragalus membranaceus* (Fisch.) Bge.	Astragaloside IV, Calycosin, Astragaloside I, Formononetin, Calycosin-7-O-β-D-glucoside, and Astragaloside II	27.27
**Dan Shen**	*Salvia miltiorrhiza*	The dried root or rhizome of *Salvia miltiorrhiza* Bge.	Tanshinol, Protocatehydehyde, Salvianolic acid B, and Tanshinone IIA	27.27
**Chuan Xiong**	*Ligusticum chuanxiong Hort*	The dried rhizome of *Ligusticum chuanxiong Hort*.	Ferulic acid, Ligustrazine and ligustichamine, and Ligustilide oxide	18.18
**GuangHuo Xiang**	*Pogostemon cablin*	The dried above-ground part of *Pogostemon cablin* (Blanco) Benth	Patchouli alcohol	18.18
**Huang Lian**	*Coptis chinensis*	The dried rhizome of *Coptis chinensis* Franch.	Berberine hydrochloride, Epiberberine, Coptisine, Berberine, and Palmatine	9.09

*Weight of each herb in one packet of QXJYG (5 g).

#### Intervention programs

Regarding the administration process, the participants will be provided with the test drugs bearing the respective drug number based on their enrollment order and will be instructed to dissolve one packet of the granules (5 g/packet) in 100 mL of hot water and take it orally twice daily. Drugs for a 2-month supply will be distributed to the participants in person at each visit. A drug management record card will be established to record the drug quantity and verify the drug numbers. Throughout the trial period, the use of other Chinese medicines for CAD, atherosclerosis, or hyperlipidemia will be prohibited. To ensure compliance, investigators will engage in weekly phone calls with all participants to reinforce the medication instructions, remind the participants of their clinic appointments before each visit, and collect all remaining drugs to assess medication adherence.

#### Withdrawal and discontinuation criteria

In cases where the patient’s condition worsens during the treatment, leading to events such as acute coronary syndrome or necessitating medication adjustments, hospitalization, or surgery, decisions regarding the adjustment of therapeutic measures or discontinuation of the study will be made by experienced physicians.

Patients with the following conditions will be withdrawn after the investigator’s assessment: 1) conditions that continue to deteriorate; 2) presence of comorbidities, complications, or notable physiological changes; 3) poor compliance or usage of prohibited drugs; 4) adverse events (AE); and 5) compromised study blinding. Patients will also have the right to withdraw voluntarily from the study at any point. In the event of voluntary withdrawal during treatment, the investigators will make every effort to maintain contact with the dropouts, inquire about the reasons for dropping out, record the time of the last dose taken, and request the dropout to complete as many assessments as possible. Furthermore, participants who withdraw due to ineffectiveness or adverse reactions, will receive appropriate medical care and financial compensation.

The clinical trial will be discontinued under the following circumstances: 1) serious safety concerns arise; 2) poor efficacy of the test drug; 3) major protocol errors or significant deviations in its implementation; and 4) discontinuation is requested by the sponsor for financial or administrative reasons.

### Outcomes

#### Efficacy outcomes

The primary efficacy outcome is CT-FFR, including the CT-FFR values of target vessels (CT-FFR_vessel_), CT-FFR values of target lesions (CT-FFR_lesion_), and the ΔCT-FFR.

The secondary outcomes are as follows: First, the imaging indicators measured using CCTA included the percentage of diameter stenosis (% DS), the percentage of area stenosis (% AS), CACS, and Gensini Score. This non-invasive quantitative CCTA measurement allows for a comprehensive and visual assessment of coronary lesions. Second, the Seattle Angina Questionnaire (SAQ) will be employed to evaluate patients’ symptom improvement. Third, considering that lipid deposition causing luminal narrowing is the main pathological change of AS and inflammatory response may increase plaque instability, levels of high-sensitivity C-reactive protein (hs-CRP), matrix metalloproteinase-9 (MMP-9), and blood lipids, including total cholesterol (TC), triglycerides (TG), low-density lipoprotein cholesterol (LDL-C), high-density lipoprotein cholesterol (HDL-C), apolipoprotein A1 (Apo A1), apolipoprotein B (Apo B), and lipoprotein (a) [Lp(a)], will also be tested. Fourth, carotid artery ultrasound parameters, including the carotid intima-media thickness (IMT; mm), carotid plaque length × thickness (mm), and carotid lumen stenosis (%), will also be evaluated. These parameters are indirect efficacy indicators, as carotid plaque and increased carotid IMT are correlated with CAD severity [28]. Finally, major adverse cardiovascular events (MACE) will also be recorded as endpoints, including non-fatal MI, cardiovascular death and revascularization therapy.

#### Safety outcomes

The primary safety outcome is the composite events of type 2, 3, or 5 bleeding as defined by the Bleeding Academic Research Consortium [29]. Secondary safety outcomes include complete blood count, coagulation function, liver and renal function, fasting blood glucose, routine urine and stool tests, and electrocardiography. AEs will be recorded throughout the trial. Periodic physical examinations will also be conducted, including vital signs and cardiac examinations.

### CCTA measurements

#### CCTA scanning

A dual-source computed tomography (CT) scanner (SOMATOM Definition Flash, Siemens Healthcare, Forchheim, Germany) will be utilized to perform CCTA scans. The procedure will be as follows: First, the participants will be instructed to hold their breath. Nitroglycerin (0.5 mg) will be administered 3 min before the examination to dilate the coronary arteries. If necessary, beta-blockers will be administered to control the heart rate to ≤70 beats/min. Next, a nonionic contrast medium, ioversol (350 mgI/mL, Libbel Flarsheim, Canada), will be injected into the patient’s right anterior elbow vein using a high-pressure syringe. The injection will deliver 60 ml of ioversol at a rate of 5.0 mL/s, followed by 40 mL of saline at the same flow rate. Third, images will be acquired in a threshold-triggered scan mode, with the region of interest (ROI) set at the root of the aorta with a threshold of 100 HU. Scan monitoring will be initiated at the start of the injection, and once the average CT value within the ROI reaches the predetermined threshold, the scanner will be triggered with a 5-s delay. The scan area will range from the carina to the diaphragm of the heart. Finally, the best phase function will be selected to determine the optimal time-phase diagram for coronary reconstruction.

#### Image post-processing and FFR analysis

All CT scanning images will be sent to the Syngo. workstation for post-processing using advanced modeled iterative reconstruction technology. This image reconstruction techniques include maximum intensity projection, multiplanar reformation, volume rendering, and curved planner reformation. Subsequently, two independent radiologists from the Radiology Department of Xiyuan Hospital will perform a quantitative image analysis in accordance with the Guideline [30]. Qualified CCTA images will be exported in DICOM format and transmitted to the DEEPVESSLE FFR (DVFFR) platform (KEYA MEDICAL Tech. Co. Ltd., China) for CT-FFR measurement using a deep machine learning model.

#### Quantification of coronary lesions

According to a consensus [31], the CT-FFR_vessel_ value should be obtained at a diameter of 2 mm distal to the target vessel. In the case of multiple vessel lesions, the CT-FFR_vessel_ value of the vessel with more severe stenosis is adopted. The CT-FFR_lesion_ value should be obtained 2 cm distal to the lesions. The ΔCT-FFR is the difference between the proximal and distal CT-FFR values of the lesions. The % DS will be calculated as [1-(minimum lumen diameter/reference vessel diameter) ×100%], whearas the % AS will be calculated as (plaque area/reference vessel area) ×100%. To ensure the consistency of the pre-/post-measurements, the most severe stenosis will be selected as the target lesion, and its distance to the coronary opening will serves as a reproducible reference index. The Gensini score employs a 15-segment model of the coronary arteries to quantify stenosis in each vessel [32]. It then calculates a total score to reflect the overall coronary stenosis in each subject [33]. The CACS employs the Agatston score to quantitatively assess the plaque burden [34]. The detailed calculation steps for these two scores are shown in **S1 and S2 Tables**.

### Adverse events

Participants will be obligated to honestly report any symptoms of discomfort during the trial and investigators will regularly monitor the patients’ physical examinations, vital signs, and laboratory tests. In the event of an AE, the investigators will provide symptomatic treatments or discontinue the intervention based on the severity of the event. Detailed information regarding the AE and possible causes will be documented in the case report form (CRF). Furthermore, in case of serious AEs, investigators will mandatorily complete the Serious Adverse Event Report Form and immediately report them to the National Medical Products Administration, the ethics committee, and the sponsor within 24 h. Simultaneously, the Data and Safety Monitoring Committee will periodically verify the reporting and handling of AEs.

### Study-specific visits and procedures

The schedule of the study procedures is illustrated in **Fig 1.** Eligible patients who have signed the ICF will be enrolled in the trial, followed by basic data collection and random allocation at baseline (visit 1). Clinical visits will take place at 2 months ± 3 days (visit 2), 4 months ± 7 days (visit 3), and 6 months ± 7 days (visit 4). Follow-up visits will take place at 12 months ± 14 days (visit 5) via phone interviews or in-hospital meetings. The efficacy will be measured at baseline (visit 1) and after 6 months of treatment (visit 4). The SAQ, physical examination, and medication adherence will be assessed every 2 months during the treatment phase. Composite events of bleeding and AEs will be recorded throughout the treatment period, while other safety outcomes will be measured at baseline (visit 1), after 2 months (visit 2) and 6 months (visit 4). MACE will be documented after the 12-month follow-up (visit 5). Additionally, the patients will be assessed by the same investigator in each procedure to maintain consistency.

### Sample size calculation

The sample size was calculated based on the estimated post-treatment CT-FFR values. The PROMISE study revealed an average CT-FFR value of 0.77 ± 0.1 for patients with ICL [35]. A previous clinical study indicated that after intervention with traditional Chinese medicine, patients with CAD had CT-FFR values of 0.92 ± 0.16 [36]. Therefore, the CT-FFR value in the control group was assumed to be 0.77, while the value in the intervention group could be elevated to 0.92 after treatment; the standard deviation (σ) was 0.13 for both groups. Results from the NXT study indicated that for every 0.05 unit decrease in CT-FFR, there was an independent correlation with an increased rate of composite cardiovascular events [37]. Based on this, we set the cut-off value to *Δ* = 0.03 in this superiority trial. The number of cases in the two groups will be allocated in a ratio of 1:1, i.e., c = 1. Given a type I error rate of α = 0.05 (two-sided test), a power of 95% (type II error rate of β = 0.05), so u_α_ = 1.96, u_β_ = 1.64, and n_1_ = n_2_ ≈ 55. Considering a maximum dropout rate of 10%, a total of 120 patients will be needed. The calculation formula is as follows:

$$n_{1}=\left(\frac{1+c}{c}\right){\left[\frac{(u_{\alpha }+u_{\beta })\sigma }{\mu_{T}-\mu_{C}-\Delta }\right]}^{2}+\frac{1}{4}u_{\alpha }^{2},\;\mathrm{and}\;n_{2}=cn_{1}$$

### Randomization and blinding

This study will be conducted using simultaneous randomization and blinding. First, the random seed, block length of 4, grouping ratio of 1:1, sample size allocation ratio to each center of 1:1:1, and a total number of patients have been determined. Based on these, a third-party statistician will use SAS 9.4 software to generate 120 random sequences. The sequence numbers arranged according to the random sequences will also serve as drug numbers. The blinding envelope containing the random sequence, drug numbers, and corresponding groups (Group A or Group B) has been sealed in duplicate and stored at the Clinical Pharmacology Center, Xiyuan Hospital of the China Academy of Chinese Medicine Sciences. Participants will get the unique drug number based on their enrollment sequence and subsequently be assigned to either the treatment or control group. The test drugs will be individually packed in large identical boxes labeled with the respective drug numbers. Anyone participating in the trial (i.e., participants, attending physicians, inspection operators, and statisticians) will remain blinded to the treatment assignments. This study will employ a two-step, unblinding process. Primary unblinding will occur after data entry and database locking, revealing the assignments of each drug number to either Group A or B. Secondary unblinding will occur after statistical analysis, revealing the treatment methods corresponding to the two groups. In addition, an emergency letter (electronic) containing drug numbers and group assignments has been prepared for situations warranting unblinding, such as serious AEs, serious complications, and patient deterioration.

### Data collection and management

Data collection will be conducted through the completion of a CRF for each enrolled patient. The baseline data collection will include demographic and clinical data. Data on vital signs, concomitant medications, and remaining test drugs will be documented at each visit. Outcome indicator results will be recorded following each measurement. The full content is provided in the **S5 File**. For participants who withdraw from the trial, the reasons for discontinuation and the results of their last measurements will also be documented in the CRF. Meanwhile, following the paper-based version of the CRF, an electronic CRF (eCRF) has been designed for data entry. To ensure the reliability of data collection, all investigators will receive training to complete the CRF, and data entry will be performed by two independent operators after each visit.

An electronic database will be created using SQL Sever2000 to facilitate data preservation, acquisition, and verification. Access to the data will be controlled through password protection and account authorization to ensure data security and confidentiality. The Clinical Pharmacology Center of Xiyuan Hospital is responsible for data management.

### Data monitoring and quality control

Data monitoring and quality control procedures will be consistently enforced throughout the study to guarantee data integrity, validity, and authenticity. A comprehensive data verification and cleaning approach will be implemented at regular 2-month intervals. First, clinical research associates (CRAs) will conduct on-site inspections at each participating center to ensure the consistency of the eCRF data with the source data. Second, an automatic logic check system will be run when data are inputted. This system will identify simple, logically contradictory, and questionable data through a predefined logic program. Third, manual checks will be performed to identify complex and specific data issues. For questionable data, the CRAs will issue a Data Clarification Form to the investigators, seeking clarification and verification. These forms, along with the raw data, will be diligently retained as source files. All error data, queries, and revision results will be documented with the audit trials. Moreover, an independent Data and Safety Monitoring Committee composed of physicians, statisticians, and medical ethicists will be responsible for data monitoring and provision of an additional layer of scrutiny.

Data for patients meeting the following criteria will be eliminated: 1) instances of inclusion criteria violations; 2) non-compliance with randomization; 3) minimal drug intake (<10%) after randomization; and 4) inability to assess efficacy and safety due to the use of prohibited drugs. Decisions regarding data elimination will be made jointly by the principal investigators, data managers, statisticians, and sponsors during a blind audit process.

### Statistical analysis

After a blind audit, data collected from the three participating centers will be consolidated and subjected to statistical analysis by an independent statistician. The analysis of efficacy indicators will adopt two primary sets: the full analysis set, in accordance with the intention-to-treat principle, and the per-protocol set, which will focus on patients who have good compliance with the protocol. The results from these two sets will be compared to assess the consistency and stability of the results. For the safety analysis set, all patients who have received at least one dose of the test drugs will be included. In cases where key efficacy data are missing, the multiple imputation method will be used to supplement the data in full analysis set.

Continuous variables will be expressed as the mean ± standard deviation or median and interquartile range, as appropriate. Categorical variables will be expressed as frequencies and percentages (%), with average ranks provided when necessary. For efficacy analysis, a linear mixed-effects model will be used to estimate the mean difference and 95% confidence interval (CI) of the primary efficacy indicator CT-FFR, adjusting for baseline values, age, gender, body mass index, blood pressure, LDL-C, fasting blood glucose, smoking and drinking history, comorbidities, and the use of PCSK-9 inhibitors as confounding factors. The SAQ score will be analyzed using a mixed model for repeated measures to estimate the mean difference and 95% CI at 2, 4, and 6 months after treatment. Other efficacy indicators will also be analyzed using the linear mixed-effects models, with baseline values as covariates. A Cox proportional hazards model will be employed to estimate the hazard ratio and 95% CI for the incidence rate of MACE. Subgroup analysis will be performed in patients with SCAD complicated diabetes and those using PCSK9 inhibitors. Safety analysis will detail a list of composite bleeding events, adverse events/reactions, abnormal laboratory safety indicators, and electrocardiography changes before and after treatment, with assessment of their clinical significance. Medication adherence will be described by actual medication dosage and durations. A kappa test will be used to guarantee the consistency of the measurements and results in this multicenter trial.

All analysis will be conducted using SAS version 9.4 software (SAS Institute, Cary, NC, USA). A two-sided test will be generally employed, and P < 0.05 will be considered statistically significant.

## Discussion

This multicenter, randomized, double-blind, placebo-controlled trial meticulously designed following the SPIRIT statement will evaluate the efficacy and safety of Chinese herbal medicine combined with conventional drug therapy for ICL in patients with SCAD. This study, using CT-FFR as the primary outcome, will provide a fresh perspective for evaluating the efficacy of QXJYG and is expected to yield reliable data supporting its therapeutic benefits.

The global increase in CAD due to population growth and aging necessitates the development of novel methods for CAD prevention and treatment. In China, there are 11.39 million patients with CAD [38], yet achieving universal health coverage to meet the substantial healthcare demands remains challenging [39]. ICL is a key stage in CAD development; hence, proactive treatment and early prevention may improve prognosis and reduce the associated disease burden [40]. However, existing drug therapies have limitations, such as adverse drug reactions and high costs.

The Chinese herbal preparation QXJYG, which treats CAD through a multi-component, multi-target, and multi-pathway mechanism, may offer enhanced efficacy and cost-effectiveness compared to single-component medications. Therefore, QXJYG combined with conventional drug therapy for ICL, may be an effective and affordable treatment strategy for SCAD. However, the toxicity of traditional Chinese herbs remains a concern. For instance, Danshen, the main ingredient in QXJYG, has major interactions with anticoagulant/anti-platelet drugs [41, 42]. This suggests an increased risk of bleeding when using QXJYG combined with anti-platelet drugs. However, no bleeding events were observed in a previous large-scale clinical study [13]. In addition, no studies have reported liver or kidney function abnormalities associated with taking QXJYG for 6 months [12, 13]. These findings provide evidence supporting the safety of QXJYG.

When it comes to assessing ICL, the key lies in identifying vulnerable plaques and myocardial ischemia. Intravascular ultrasound (IVUS) and optical coherence tomography (OCT) are recognized methods for evaluating plaque morphology, such as identifying thin-cap fibroatheromas [43, 44]. The ACC/AHA guideline affirmed the importance of coronary physiological assessment in patients with ICL [6]. However, the invasive nature and high cost of these examinations hinder their widespread clinical use. Recently, non-invasive quantitative CCTA has evolved into a comprehensive tool for assessing plaque morphology, vessel anatomy, and function with high accuracy, making it a valuable alternative to invasive examinations [45]. Adverse plaque characteristics visualized by CCTA are highly correlated with those on OCT and IVUS [46, 47]. Additionally, numerous studies utilized plaque parameters from CCTA to evaluate treatment efficacy and disease progression [48–50]. However, the functional assessment based on CT-FFR has not been sufficiently considered in previous studies. CT-FFR has shown diagnostic accuracy consistent with invasive FFR [51]. It also addresses the limitations of functional assessment with CCTA [52, 53]. Even in the presence of calcification, CT-FFR can facilitate the identification of ischemic lesions in addition to CCTA [54]; this is attributed to the optimization of machine learning-based CT-FFR through artificial intelligence algorithms and the 3D reconstruction of the coronary arteries, resulting in higher accuracy. Therefore, we employed CT-FFR as the primary outcome. On the one hand, CT-FFR is particularly valuable for patients with ICL [55]. It has been shown to decrease the need for invasive coronary angiography and have high safety [56, 57]. Furthermore, CT-FFR is negatively correlated with the occurrence of cardiovascular events [37, 58]. It combined with other parameters from CCTA holds superior predictive performance for long-term prognosis [59]. On the other hand, CT-FFR may aid in identifying vulnerable plaques. Driessen et al. demonstrated a correlation between decreased FFR and adverse plaque characteristics visualized on CCTA [60]. This correlation may be attributed to factors such as endothelial dysfunction, inflammation, and alterations in shear stress patterns during the formation of adverse plaques, which ultimately lead to impaired coronary vasodilation and abnormal FFR. Additionally, the feasibility of using CT-FFR as an outcome to assess clinical efficacy has been demonstrated [36, 61, 62], and the improvement in hemodynamics measured using CT-FFR may be attributed to enhanced vascular dilation capability and reduction in the length of plaque lesions. Therefore, in this multicenter trial, coronary CT imaging is a reasonable measurement tool for monitoring the efficacy of the test drugs. An improvement in the CT-FFR in the study results will indicate that QXJYG treatment can enhance coronary blood supply and potentially reduce the occurrence of cardiovascular events.

Inflammation and lipid levels are the secondary concerns in this study. The PROSPECT study emphasized the significance of thin-cap fibroatheromas leading to MACE [43]. The effective control of blood lipids and inflammation is imperative to counteract the formation and rupture of such plaques. Among the various inflammatory factors, hs-CRP is a highly accurate predictor of cardiovascular risk [9]. Additionally, MMP-9 is a potent predictor of plaque instability in patients with SCAD and is closely associated with inflammation [63]. Therefore, this study will use hs-CRP, MMP-9, and blood lipid levels to verify the anti-inflammatory and lipid-regulating effects of QXJYG, ultimately determining its potential to prevent MACE.

This study may also have some limitations. First, when measuring the primary outcome, the CT-FFR may not be calculated due to poor CT image quality. To prevent this, we have standardized the measurement process. Initially, restrictions have been imposed on the patient’s heart rate and CACS upon enrollment. Subsequently, a 256-slice dual-source CT will be used for examination, preceded by heart rate control and breath-hold training. These measures will help eliminate motion and calcification artifacts on the CT images. Second, positive results of cardiovascular endpoint events may not be obtained due to the low occurrence of MACE. Therefore, we intend to continue the follow-up even after the end of the study to assess the long-term outcomes of the intervention. Third, although this is a multicenter study, patients will be recruited only from the three hospitals in Beijing equipped for CT-FFR computation. As the CT-FFR technology matures and spreads, subsequent studies can be expanded to more regions.

In conclusion, this study is expected to deliver dependable results through meticulous design and rigorous implementation. A favorable result will support that treatment of ICL with QXJYG could improve the efficacy and safety of existing conservative treatments and prolong the time to revascularization. This will help to address the prevailing therapeutic dilemma of ICL, thereby providing innovative ideas for the prevention and treatment of SCAD.

## Supporting information

S1 Fig Striking image. (TIF)

S1 Table Illustration of the steps for calculating the Gensini score. (DOCX)

S2 Table Illustration of the steps for calculating the CACS. (DOCX)

S1 File Study protocol submitted to the ethics committee (English version). (PDF)

S2 File Study protocol submitted to the ethics committee (Chinese version). (PDF)

S3 File SPIRIT checklist. (PDF)

S4 File Drug quality inspections report (Chinese and English version). (PDF)

S5 File Case report form. (PDF)

## List of abbreviations

AE: Adverse event
CACS: Coronary artery calcification score
CAD: Coronary artery disease
CCTA: Coronary computed tomography angiography
CI: Confidence interval
CRA: Clinical research associate
CRF: Case report form
CT-FFR: CT-derived fractional flow reserve
Hs-CRP: High-sensitivity C-reactive protein
ICF: Inform consent form
ICL: Intermediate coronary lesion
LDL-C: Low-density lipoprotein cholesterol
MACE: Major adverse cardiovascular event
MI: myocardial infarction
MMP-9: Matrix metalloproteinase-9
PCSK-9: Proprotein convertase subtilisin/kexin type 9
QXJYG: Qing-Xin-Jie-Yu Granules
SAQ: Seattle angina questionnaire
SCAD: Stable coronary artery disease
% AS: Percentage of area stenosis
% DS: Percentage of diameter stenosis
