# Supplementary figures and images for: Chinese herbal compound preparation Qing-Xin-Jie-Yu granules for intermediate coronary lesions in patients with stable coronary artery disease: Study protocol for a multicenter, randomized, double-blind, placebo-controlled trial
Source: PLoS One. 2024 Jul 16;19(7):e0307074. doi: 10.1371/journal.pone.0307074 (PMC11251585; doi:10.1371/journal.pone.0307074)

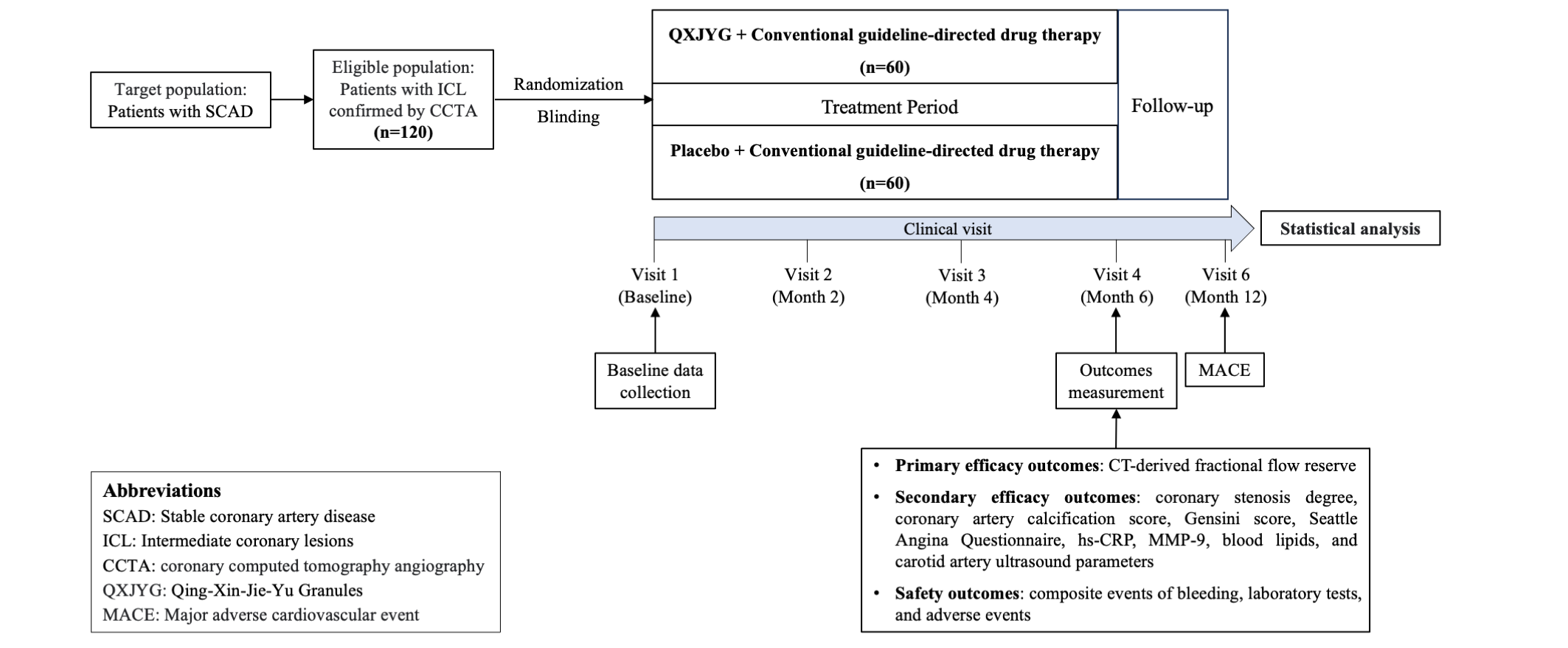

Supplement: S1 Fig — (TIF) [file pone.0307074.s001.tif]
