# Supplementary material for: Chinese herbal compound preparation Qing-Xin-Jie-Yu granules for intermediate coronary lesions in patients with stable coronary artery disease: Study protocol for a multicenter, randomized, double-blind, placebo-controlled trial
Source: PLoS One. 2024 Jul 16;19(7):e0307074. doi: 10.1371/journal.pone.0307074 (PMC11251585; doi:10.1371/journal.pone.0307074)
Supplement: S1 Table — (DOCX) [file pone.0307074.s002.docx]

**S1 Table.** Illustration of the steps for calculating the Gensini Score.

| **Step 1:** Calculation of the severity score for each lesion. | |
| --- | --- |
| *Degree of stenosis (%)* | *Severity Score* |
| 1-25 | 1 |
| 26-50 | 2 |
| 51-75 | 4 |
| 76-90 | 8 |
| 91-99 | 16 |
| 100 | 32 |
| **Step 2:** Determination of the multiplication factor of each lesion based on its location in the coronary tree | |
| *Segment** | *Multiplication factor* |
| RCA proximal | 1 |
| RCA mid | 1 |
| RCA distal | 1 |
| PD | 1 |
| LM | 5 |
| LAD proximal | 2.5 |
| LAD mid | 1.5 |
| LAD apical | 1 |
| 1^st^ diagonal | 1 |
| 2^nd^ diagonal | 0.5 |
| LCX proximal | 2.5(right dominance) or 3.5(left dominance) |
| OM | 1 |
| LCX apical | 1(right dominance) or 2(left dominance) |
| PD | 1 |
| PL | 0.5 |
| **Step 3:** Sum of all the lesion severity scores. | |

*According to the American Heart Association 15-segment model. RCA: right coronary artery; LM: left main coronary artery; LAD: left anterior descending branch; LCX: left circumflex branch; OM: obtuse marginal branch; PD: posterior descending branch; PL: posterior branches of left ventricul.
