# Supplementary material for: Chinese herbal compound preparation Qing-Xin-Jie-Yu granules for intermediate coronary lesions in patients with stable coronary artery disease: Study protocol for a multicenter, randomized, double-blind, placebo-controlled trial
Source: PLoS One. 2024 Jul 16;19(7):e0307074. doi: 10.1371/journal.pone.0307074 (PMC11251585; doi:10.1371/journal.pone.0307074)
Supplement: S2 Table — (DOCX) [file pone.0307074.s003.docx]

**S2 Tabl****e.** Illustration of the steps for calculating the CACS*.

| **Step 1:** Quantification of plaque area (mm^2^) with CCTA | |
| --- | --- |
| **Step 2:** Determination of a lesion score in the following manner: | |
| *CT value (HU)* | *Density score* |
| 130-199 | 1 |
| 200-299 | 2 |
| 300-399 | 3 |
| ≥ 400 | 4 |
| **Step 3:** Calculation of the score for each region: a region score = density score × the area. | |
| **Step 4:** Sum of all the region scores | |

*The threshold for a calcified lesion is a plaque with an area ≥ 1mm^2^ and CT value > 130 HU.
