# Supplementary material for: Chinese herbal compound preparation Qing-Xin-Jie-Yu granules for intermediate coronary lesions in patients with stable coronary artery disease: Study protocol for a multicenter, randomized, double-blind, placebo-controlled trial
Source: PLoS One. 2024 Jul 16;19(7):e0307074. doi: 10.1371/journal.pone.0307074 (PMC11251585; doi:10.1371/journal.pone.0307074)
Supplement: S1 File — (PDF) [file pone.0307074.s004.pdf]

Chinese herbal compound preparation Qing-Xin-Jie-Yu granules for intermediate coronary lesions in patients with stable coronary artery disease: a randomized, double-blinded, placebo-controlled trial

Study Protocol

Sponsor Unit: Xiyuan Hospital of China Academy of Chinese Medical Sciences

Leader Unit: Xiyuan Hospital of China Academy of Chinese Medical Sciences

Member Unit: Guan'anmen Hospital of China Academy of Chinese Medical Sciences; Beijing Tongren Hospital Affiliated to Capital Medical University

Version Number: XYYY-V-1.0

Version Date: December 26, 2021

1. Study objective

To evaluate the efficacy and safety of Qing-Xin-Jie-Yu Granules (QXJYG) for intermediate coronary lesions (ICL) in patients with stable coronary artery disease (SCAD).

2. Participants

2.1 Diagnostic criteria

1) Diagnosis criteria of ICL: 2021 ACC/AHA/SCAI Guideline for Coronary Artery Revascularization.
2) Diagnosis criteria of SCAD: 2014 ACC/AHA/AATS/PCNA/SCAI/STS focused update of the guideline for the diagnosis and management of patients with stable ischemic heart disease; 2021 AHA/ACC/ASE/CHEST/SAEM/SCCT/SCMR Guideline for the Evaluation and Diagnosis of Chest Pain.

2.2 Inclusion criteria

1) Patients with SCAD who have at least one major coronary artery with luminal diameter stenosis of 50% to 70% confirmed by coronary computed tomography angiography (CCTA).
2) Heart function grade NYHA grade Ⅰ-Ⅱ.
3) Patients aged 18 to 75 years.
4) Patients who are informed and voluntarily sign the ICF.

2.3 Exclusion criteria

1) Patients have previously undergone coronary stent implantation, coronary artery bypass grafting or MI within three months.
2) Lesions with diameter stenosis ≥ 50% in the left main coronary artery or in all three major arteries.
3) Diffuse lesions throughout the diseased vessels.
4) Severe blood pressure fluctuations or difficult-to-control hypertension with systolic blood pressure ⩾ 160mmHg and diastolic blood pressure ⩾ 100mmHg in the past 3 months.
5) Resting heart rate > 100 beats/min and difficult to control.
6) Patients have previously undergone other cardiac operations, such as valve replacement.
7) Patients with severe cardiac, hepatic or renal insufficiency that are unsuitable for CCTA and related examination and treatment.
8) Patients suffering from mental disorders.
9) Patients suffering from hepatitis, tuberculosis, AIDS, or other infectious diseases.
10) Allergic sufferers.
11) Pregnant and lactating women
12) Life expectancy is less than one year.
13) Participation in other clinical trials within the last three months.

2.4 Elimination criteria

1) Cases where the patient selection violates the inclusion/exclusion criteria and should not have been randomized.
2) Subjects who do not cooperate with randomization or do not take any investigational drugs after randomization, or use drugs very minimally (<10%).
3) Cases where prohibited drugs were used, making it impossible to assess efficacy and safety.
4) No data available after randomization.

The decision to exclude such cases must be made during the blind review by the principal investigator, data manager, statistical analysis expert, and sponsor after discussion.

2.5 Withdrawal criteria

1) Conditions continue to worsen.
2) Comorbidities, complications, or special physiological changes.
3) Poor compliance or use of prohibited drugs.
4) Adverse events or serious adverse events.
5) Leak of blindness

2.5.1 Determination of dropout cases

Subjects who have completed the informed consent form (ICF) and qualified for entry into the trial but withdraw for any reason before completing the specified observation period, are considered as dropout cases. The reasons for dropout should be documented. If baseline efficacy data are available, the last recorded primary efficacy results should be used as the final result for statistical analysis, and the case report forms (CRFs) should be retained for reference.

2.5.2 Handling of dropout cases

After a subject drops out, the investigator should maintain contact with the subject, inquire about the reasons for dropout, record the last medication administration time, and make the dropouts complete as many assessment items as possible. Then the CRF should be completed. If someone withdraws due to allergic reactions, adverse events, or ineffective treatment, the investigator should provide appropriate treatment measures and some form of financial compensation to ensure the subject's rights.

All enrolled cases, regardless of dropout status, should have their data recorded and retained in the CRF. These records are essential for the Full Analysis Set (FAS) analysis. Dropout cases do not need to be supplemented separately.

2.6 Termination criteria

1) Discovery of severe safety issues during the trial.
2) The efficacy of the test drug is poor or even ineffective.
3) Identification of major errors in the study protocol or important deviations in its implementation, making it difficult to evaluate the drug's efficacy.
4) Request for trial termination by the sponsor due to financial reasons or management issues.

3. Study design

This study is designed as a multicenter, randomized, double-blind, placebo-controlled trial.

3.1 Multicenter

This study is simultaneously conducted at 3 centers: Xiyuan Hospital of China Academy of Chinese Medical Sciences; Guan'anmen Hospital of China Academy of Chinese Medical Sciences; Beijing Tongren Hospital Affiliated to Capital Medical University.

3.2 Randomization.

Stratified and block randomization methods are employed. SAS statistical software is used to generate the 'Center Code Randomization Table' based on the number of cases from participating units and the randomization ratio. The selected block length, randomization seed parameters, and other confidential data were sealed in the blind envelope. Unrelated personnel encoded the drugs according to this randomization table. The intervention group and control group are allocated in a 1:1 ratio.

3.3 Control

Placebo-controlled.

3.4 Sample size calculation

The sample size is calculated based on the estimated post-treatment CT-FFR values. The PROMISE study revealed an average CT-FFR value of 0.77 ± 0.1 for patients with ICL. A clinical study indicated that after traditional Chinese medicine intervention, patients with CAD had CT-FFR values of 0.92 ± 0.16. Therefore, it was assumed that the CT-FFR value in the control group is 0.77 and the value in the intervention group could be elevated to 0.92 after treatment, the standard deviation σ = 0.13 for both two groups. Results from the NXT study indicated that for every 0.05 unit decrease in CT-FFR, there was an independent correlation with the increasing rate of composite cardiovascular events. Based on this, we have set the cut-off value Δ=0.03 in this superiority trial. The number of cases in the two groups is arranged in a ratio of 1:1, so c=1. Given a type I error rate of α = 0.05 (two-side test), a power of 95% (type II error rate of β = 0.05), so u_α=1.96, u_β=1.64, n_1=n_2≈55. Considering the maximum dropout rate of 10%, a total of 120 patients is needed. The calculation formula is as follows:

$$n_1 = \left(\frac{1+c}{c}\right)\left[\frac{(u_\alpha + u_\beta)\,\sigma}{\mu_T - \mu_C - \Delta}\right]^2 + \frac{1}{4}u_\alpha^2, \text{ and } n_2 = cn_1$$

3.5 Blinding Method

A double-blind design is adopted. The first level is the group corresponding to each case number (e.g., Group A, Group B), and the second level is the treatment corresponding to each group (experimental group, control group).

3.5.1 Blinding and Blind Envelope Retention

Random numbers are generated using SAS statistical software package based on a stratified randomization method, and drugs are blinded and packaged according to these random numbers. The trial utilizes a double-blind, double-dummy technique, and all study drugs and control drugs should be packaged in the same manner, and blinded according to the random numbers. Both the investigational drug and the placebo should be uniformly packaged, ensuring that the appearance of the active drug and the placebo is indistinguishable. The blinding process is jointly completed by the head of the responsible clinical research unit, the sponsor, and the statistical personnel. Two copies of the blind envelopes are prepared and sealed, and they are kept by the primary research unit and the sponsor. During the trial, the blind envelope cannot be opened or read.

3.5.2 Emergency Unblinding

Along with the drug packaging and coding, an emergency envelope is prepared for each drug, containing the sealed information about the group assignment corresponding to the drug's code. In the event of a medical emergency, the emergency envelope (emergency unblinding) can be opened. Emergency unblinding may be considered in the following situations, but not limited to:

a) When a patient experiences severe adverse reactions/adverse events.
b) When a patient experiences severe complication.
c) When urgent measures are required due to deteriorating symptoms.

In the case of an emergency requiring unblinding, the researcher must seek approval from the head of the center, and upon obtaining their signed agreement, the emergency unblinding envelope can be opened. Detailed records of the unblinding reason, time, location, and signatures must be made. Within 24 hours of unblinding, the head of the clinical research team and relevant personnel from the sponsor should be notified, and the reasons for unblinding should be explained. Cases that withdrew due to efficacy reasons should not be unblinded. Data related to unblinded cases should be kept complete.

3.5.3 Unblinding Protocol

This study adopts a two-step unblinding process. Once all Case Report Forms (CRFs) have been entered into the database and the statistical analysis plan is finalized following clarifications, verifications, and blind review, the database will be locked. At this stage, the first unblinding will take place, conducted jointly by the principal investigator of the study and the statistical personnel. During this step, the blind codes corresponding to each drug (Group A or Group B) will be revealed to facilitate grouping and statistical analysis of all data. After the analysis is completed, the second unblinding will be carried out by the principal investigator at the clinical research summary meeting, disclosing the treatment measures corresponding to Group A and Group B. Detailed records of all unblinding processes should be maintained.

4. Intervention

4.1 Drug Administration

All eligible patients will be randomly assigned to either the experimental group or the control group. Both groups will receive conventional Western medical treatment according to the guidelines for coronary heart disease. Additionally, the experimental group will be treated with a clinically effective traditional Chinese medicine, named Qing-Xin-Jie-Yu Granules. The control group will receive a placebo treatment. The treatment period will last for 6 months, followed by a 12-month follow-up.

4.2 Drug Packaging

Each participant's medication will be packaged in a large package, consisting of 3 medium packages and a convenience bag containing a 2-day reserve of medication. The dosage for one visit corresponds to one medium package, and the 2-day reserve dosage is separately packaged in a plastic convenience bag and stored inside the large package box. Both the large and medium packages must have labels affixed, indicating the drug number.

4.3 Drug Management

Drugs should be uniformly managed and stored. The researchers will establish a clinical trial drug management record card to inspect the quantity, quality, and correctness of drug coding. The drugs will be dispensed, and their usage will be recorded by designated personnel. After the trial is completed, another designated person will be responsible for the collection and proper disposal of any remaining drugs.

4.4 Combination Drug.

1) During the trial, participants are not allowed to take other traditional Chinese medicines used to treat coronary heart disease, atherosclerosis, or hyperlipidemia simultaneously.
2) If other medications are necessary for the treatment of other diseases, they can be continued. However, the names of the drugs (or other treatment methods), dosages, frequency of use, and timing must be documented in CRF for analysis and reporting.

5. Outcomes

5.1 General information

1) Demographic indicators: date of birth, gender, height, weight, ethnicity, occupation, body mass index (BMI), blood pressure.
2) General clinical data: present medical history, past medical history, smoking history, alcohol consumption history, and medication history.

The past medical history focuses on inquiring about early-onset cardiovascular disease family history (occurrence of the disease in first-degree relatives before the age of 55 in men and 65 in women), history of cerebrovascular disease, peripheral vascular disease, diabetes, hypertension, and dyslipidemia.

5.2 Efficacy outcomes

5.2.1 Primary efficacy outcomes

1) CT-derived fractional flow reserve (CT-FFR).

5.2.2 Secondary efficacy outcomes

1) Imaging indicators measured by CCTA: the percentage of diameter stenosis (% DS), the percentage of area stenosis (% AS), coronary artery calcification score (CACS), and Gensini Score.
2) Seattle angina questionnaire (SAQ).
3) Inflammatory factors: high-sensitivity C-reactive protein (hs-CRP), matrix metalloproteinase-9 (MMP-9).
4) Blood lipids: total cholesterol (TC), triglycerides (TG), low-density lipoprotein cholesterol (LDL-C), high-density lipoprotein cholesterol (HDL-C), apolipoprotein A1 (Apo A1), Apolipoprotein B (Apo B) and lipoprotein (a) [Lp(a)].
5) Carotid artery ultrasound: carotid intima-media thickness (IMT) (mm), carotid plaque length × thickness (mm), and carotid lumen stenosis (%).

5.3 Safety outcomes

5.3.1 Primary safety outcome

1) the composite events of type 2, 3, or 5 bleeding as defined by the Bleeding Academic Research Consortium (BARC).

5.3.2 Secondary safety outcomes

1) Coagulation function: prothrombin time (PT), thrombin time (TT), international normalized ratio (INR), activated partial thromboplastin time (APTT).
2) Complete blood count: red blood cells (RBC), hemoglobin (HB), white blood cells (WBC), neutrophil percentage (NEUT%), platelets (PLT).
3) Urinalysis: urine protein (PRO), urine glucose (GLU), urine red blood cells, urine white blood cells.
4) Stool routine: white blood cells, fecal occult blood.
5) Liver function: alanine aminotransferase (ALT), aspartate aminotransferase (AST).
6) Renal function: blood urea nitrogen (BUN), uric acid (UA), serum creatinine (Scr).
7) Blood glucose.
8) Electrocardiogram.

5.4 Endpoint

1) Major adverse cardiovascular events (MACE): non-fatal myocardial infarction, cardiovascular death, and revascularization.

6. Follow-up

The treatment period will last for 6 months, and the follow-up will be a total of 12 months, with visits scheduled at the following time points: before treatment (visit 1),

2 months after treatment (visit 2), 4 months after treatment (visit 3), 6 months after treatment (visit 4), and 12 months after treatment (visit 5).

The process of discontinued case exiting the trial:
a) Retrieve and record remaining drugs and packaging.
b) Inquire about any adverse events or other relevant information.
c) Perform a physical examination.
d) Complete efficacy and safety evaluation.
e) Record the reason for discontinuation.

7. Adverse events

7.1 Records of Adverse events.

Upon discovering an adverse event, the researcher should immediately document it in the original records and case report forms. If the adverse event qualifies as a serious adverse event, a new adverse drug reaction, or a severe adverse drug reaction, it should be reported following the appropriate procedures.

7.2 Judgment of Adverse events

7.2.1 Severity Assessment

Mild	The subject can tolerate the adverse event, and it does not impact the treatment. No special treatment is required, and it has no effect on the subject's recovery.
Moderate	The subject finds it difficult to tolerate the adverse event, and it may require discontinuation of the drug or special treatment. It directly affects the subject's recovery.
Severe	The adverse event poses a threat to the subject's life, and it can result in death or disability. Immediate discontinuation of the drug or emergency treatment is required.

7.2.2 Analysis method

According to China's "Measures for the Management of Adverse Drug Reaction Reporting and Monitoring", mainly following the five principles:

a) Is there a reasonable temporal relationship between drug use and the occurrence of adverse reactions/events? Yes □ No □
b) Does the reaction correspond to a known adverse reaction type of the drug? Yes □ No □ Unclear □
c) After discontinuation or dose reduction, did the reaction/event disappear or decrease? Yes □ No □ Unclear □ Not discontinued or dose not reduced □
d) After the suspected drug is used again, did the same reaction/event reoccur? Yes □ No □ Unclear □ Not used again □
e) Can the reaction/event be explained by the effect of drugs, the progression of the patient's condition, or the influence of other treatments? Yes □ No □ Unclear □

7.2.3 Association Evaluation

Based on the five principles above, the association evaluation is classified into six levels: "Definite", "Very likely", "Probable", "Possible unrelated", "To be evaluated", and "Unable to evaluate". Among them, levels 1, 2, and 3 (Definite, Very likely, Probable) are considered as adverse drug reactions, indicating a certain or strong association with drug use. Other levels require further assessment or data collection to determine the association.

	1	2	3	4	5
Definite	+	+	+	+	-
Very likely	+	+	+	?	-
Probable	+	±	± ?	?	± ?
Possible unrelated	-	-	± ?	?	± ?
To be evaluated	There is a need for supplementary materials to evaluate				
Unable to evaluate	The necessary information cannot be obtained				

Noted: "+" indicates a positive association; "-" indicates a negative association; "±" indicates uncertain or difficult to determine; "?" indicates unclear or unknown association.

7.3 Outcomes of Adverse Event

Death	The event leads to the end of life (the cause of death and time of death should be collected).
Not Recovered/ Not Resolved	After treatment, the adverse event's symptoms did not improve or recover.
Recovered	After treatment, the adverse event's symptoms completely disappeared, and there were no residual symptoms.
Symptoms Disappeared with Residual Effects	After treatment, the adverse event's symptoms disappeared, but there are residual effects (the name or manifestation of the residual effect should be specified when recording).
Improved	After treatment, the adverse event's symptoms improved.
Not Specified	The situation is unknown, not followed up, recorded, or the subject refused to provide further information.

7.4 Reporting of Adverse Events:

General adverse events will be recorded in the CRF without the need for specific reporting.

Serious adverse events will be reported within 24 hours of becoming aware of the event, completing a Serious Adverse Event Report form, and submitted to National Medical Products Administration (NMPA), Drug Regulatory Authorities where the research center is located, Ethics Committee, and the sponsor. Written records of the report should also be retained.

8. Clinical Data Management

8.1 Electronic Data Management.

This study utilizes SQL Server 2000 to establish a database for data entry and

management. A designated individual is responsible for data management and confidentiality.

8.2 Data Management Plan

1) The Data Management Plan (DMP) will be authored by the Project Data Manager (PL).
2) Before the study commences, the Director of Data Management (DMD) will review the DMP to ensure it includes all the required steps and information.
3) The DMP must receive approval from the sponsor before implementation.

8.3 Electronic Case Report Forms (eCRF)

1) The PL will create the eCRF pages based on the paper-based CRF.
2) Once the pages are designed by PL, they will be handed over to another PL2 not involved in the current project for testing. After successful testing, PLs will submit an eCRF release application to the DMD.

8.4 Data Entry

1) Each center will have two clinical research associates (CRA) responsible for data entry.
2) The research data will be entered into the Drug Clinical Trial Data Management System of Xiyuan Hospital according to the scheduled follow-up time points.
3) Independent double data entry will be performed.
4) The eCRF is not considered the original record; its content is sourced from the original CRF.

8.5 Data Verification and Cleaning

8.5.1 Data Verification Plan

The PL will develop a Data Verification Plan (DVP) based on the eCRF. The DVP should encompass thorough verification of primary and secondary efficacy outcomes, as well as critical safety indicators. Data verification should be conducted without knowledge of the treatment groups, and any data discrepancies or issues will be recorded in a Data Clarification Form (DCF).

8.5.2 Data Verification Methods

a) Source Data Verification (SDV). The monitor will log into the data monitoring section of the data management system at each research center to perform on-site verification. This involves a 100% check of the eCRF data against the source data.

   Any inconsistencies will be flagged and questioned online.

b) System Automated Logic Checks. PL will analyze the DVP and identify portions suitable for automated system checks. Programs will be developed and integrated into the eCRF pages. During data entry, the computer will automatically verify these data to enhance data entry quality and data management efficiency.

c) Manual Checks. Data that cannot be automatically verified by the computer will undergo manual online review. If any doubts arise during the process, they can be directly communicated by clicking on a query.

8.6 Queries handling

1) Issuing. Data entry personnel will regularly log into the system to download the Data Clarification Form (DCF) containing queries specific to their institution.
2) Answering. During the data cleaning process, PL or monitors will raise all data queries in the form of DCF. Researchers will be required to provide written responses and sign the DCF. Data modifications should only be made on the DCF, which will be preserved as the source document along with the original data in the research medical records.
3) Online Updates. Data entry personnel will perform online updates based on the responses provided in the DCF.
4) Resolution. PL or monitors will review the updated data until all queries are resolved.

8.7 Data Review Meeting

During the meeting, PL presents the data management status. And the sponsor, principal investigator, data management personnel, and statisticians collectively review unresolved data issues. According to the statistical plan, they discuss data set partitioning, verification of serious adverse event reports, and handling records.

Once a consensus is reached on all queries and data analysis set, and after updating the data based on the data review opinions, the data manager will lock the database upon receiving approval. The clean database will then be handed over to the statistical team.

8.8 Database Locking and Unlocking

8.8.1 Database Locking

The database locking requires to fulfill the following prerequisites:

a) All data has been correctly entered into the database.

b) All data clarification forms have been resolved and incorporated into the database.

c) The eCRFs have been reviewed and approved by the researchers.

d) Medical coding has been completed.

e) Final data and logical consistency checks have been performed and reviewed.

f) Final review of evident errors or abnormalities has been conducted.

g) All trial-related documents have been updated and saved in accordance with standard operating procedures.

After confirming that the trial meets the above criteria, PL submits a written request for database locking and obtains signatures from relevant trial personnel (including DMD, biostatisticians, clinical monitors, and principal investigators) on the written approval documents.

8.8.2 Database Unlocking

After the database has been locked, if any erroneous data is identified, PL submits a written request for unlocking along with the data audit findings. PL unlocks the database and revises the data according to the data audit findings. After the necessary revisions are made, the database is locked again. All revised data are traceable and maintain an audit trail.

9. Statistical Analysis

All statistical tests will be conducted as two-tailed tests, and a P-value ≤ 0.05 will be considered statistically significant for the tested differences.

9.1 Population for Statistical Analysis

1) Intent-to-Treat (ITT) Analysis: All participants who express the intention to receive treatment and sign the informed consent form will be included in the analysis.
2) Full Analysis Set (FAS): This includes participants who signed the informed consent form, received the investigational drug at least once, and have corresponding efficacy indicator records. Baseline data analysis will focus on the FAS as the target population, and efficacy analysis will be based primarily on FAS results.
3) Per-Protocol Set (PPS): This includes participants who meet the inclusion criteria, do not meet the exclusion criteria, and complete the treatment regimen. PP analysis involves analyzing participants who adhere well to the trial protocol.
4) Safety Set (SS): This includes participants with safety indicator records. Missing values for safety indicators will not be estimated.

9.2 Statistical Analysis method

9.2.1 General Considerations

1) All statistical tests will be two-sided, with a P-value ⩾ 0.05 considered statistically significant.
2) Statistical description: Continuous variables will be expressed as mean ± standard deviation, median, and interquartile range. Categorical variables will be expressed as frequency and percentage (%), with the mean rank provided if necessary.
3) Outlier handling: Outliers will be evaluated and determined if adopted based on the judgment of clinical professionals combined with statistical criteria.
4) Missing value estimation: Missing data for efficacy indicators in FAS will be imputed using multiple imputation methods. Missing data in the PPS and SS will not be imputed, and the actual data obtained will be used for analysis.

9.2.2 Efficacy Analysis

1) Primary Efficacy Outcome: CT-FFR.

- Description: CT-FFR values of the target vessels at baseline and 6 months after

treatment, as well as the difference from baseline to 6-month will be described. For multiple vessel lesions, the CT-FFR value of the most severely narrowed vessel will be selected.

- Intergroup Comparison: A linear mixed model will be used for comparison between groups. The dependent variable is the difference in CT-FFR from baseline to 6-month. Fixed effects include group and center, while random effects include subjects. Covariates include baseline values of CT-FFR, age, gender, BMI, systolic blood pressure, diastolic blood pressure, LDL-C, FBG, smoking status, alcohol consumption, family history of early-onset cardiovascular disease, presence of cerebrovascular disease, peripheral vascular disease, diabetes mellitus, hypertension, dyslipidemia, and concomitant use of PCSK-9 inhibitors.
- Estimation: The least squares method will be used to estimate mean differences and 95% confidence intervals (CI).

2) Secondary Efficacy Outcomes

a) Coronary imaging assessment indicators

- Description: %DS measurement will use the most narrowed location as the result; CACS and Gensini scores will use the total score as the result. Values of %DS, CACS, and Gensini scores at baseline and 6 months after treatment, as well as the difference from baseline to 6-month will be described.
- Intergroup Comparison. A linear mixed model will be used, with the dependent variable being the difference from baseline to 6-month. Fixed effects include group and center, random effects include subjects, and covariates include baseline values.
- Estimation: The least squares method will be used to estimate the mean difference and 95% CI.

b) SAQ score

- Description: SAQ scores at baseline, 2, 4, and 6 months after treatment will be described, along with the differences from baseline to 2, 4, and 6 months.
- Intergroup Comparisons: A mixed model for repeated measures will be used, with the dependent variable being the difference in SAQ scores at 2, 4, and 6 months relative to baseline, with the primary assessment time point at 6 months after treatment. Fixed effects include group and center, random effects include subjects. Covariates include baseline SAQ scores, visit times, the interactions between baseline and visit, and the interactions between treatment group and visit.
- Estimation: The least squares method will be used to estimate the mean difference between groups and the 95% CI.

c) Serum inflammatory factors, blood lipids, and carotid ultrasound indicators

- Description: Values at baseline and 6 months after treatment, as well as the difference from baseline to 6-month will be described.
- Intergroup Comparisons: A linear mixed model will be used, with the dependent variable being the difference from baseline to 6-month. Fixed effects include group and center, and random effects include the subjects, with baseline values as covariates.

- Estimation: The least squares method will be used to estimate the mean difference and 95% CI.

3) Endpoint Event: MACE

- Description: The incidence rate of MACE from baseline to the 1-year follow-up will be described.
- Intergroup Comparisons: The Cox proportional hazards model will be used, with the dependent variable being the occurrence of MACE and the survival time variable being the time from baseline to either the occurrence of MACE or the end of follow-up. The primary independent variable is the group. Covariates include age, gender, BMI, systolic blood pressure, diastolic blood pressure, LDL-C, FBG, smoking status, alcohol consumption, family history of early-onset cardiovascular disease, presence of cerebrovascular disease, peripheral vascular disease, diabetes mellitus, hypertension, dyslipidemia, concomitant use of PCSK-9 inhibitors, and study center.
- Estimation: The partial likelihood method will be used to estimate the hazard ratio and its 95% CI.

4) Subgroup analyses: Considering the significant impact of concomitant diabetes and the use of PCSK-9 inhibitors on drug efficacy, subgroup analysis of the primary efficacy outcome and endpoint events will be performed in SCAD patients with diabetes and SCAD patients with the use of PCSK-9.

9.2.3 Safety analysis

1) Primary safety outcome: A detailed list of composite bleeding events will be provided.
2) Secondary safety outcomes: A statistical description of pre- and post-treatment laboratory safety measurements will be presented. Cross-tabulations of pre- and post-treatment laboratory safety indicators and ECG will be listed to assess their clinical significance in terms of normal/abnormal transitions.
3) Adverse events: A detailed list of adverse events/reactions will be provided, along with an assessment of their relationship to the test medication.

9.2.4 Medication adherence analysis

The actual dosage and duration of medication will be described to assess medication adherence.

9.3 Statistical software

The SAS version 9.4 (SAS Institute, Cary, NC, USA) will be used for all the analyses.

10. Quality Control

10.1 Data Quality Assurance

1) For most laboratory tests, efforts are made to perform the measurements in the same laboratory using the same instrument and reagents from the same batch. In cases where the same laboratory test is performed in two or more laboratories, it is required to use the same model of instrument, reagents from the same batch,

and follow the same operating procedures. Regular comparison of laboratory measurement results is also carried out.

2) Periodic quality control checks are conducted for all major equipment and instruments.

10.2 Quality Control Methods

1) Develop detailed work manuals and provide standardized training for researchers and laboratory technicians. All instrument operators and laboratory technicians must undergo uniform training and assessment. The consistency among different operators or technicians is assessed using the Kappa statistical test, with a requirement that the Kappa value should be above 0.85.
2) Establish a quality control team consisting of inspectors who regularly inspect the original data from each center and verify the consistency between the original data and the entered data.

11. Ethics Requirements

11.1 Ethics Review

This clinical study follows the principles of the Helsinki Declaration and relevant clinical research regulations in China. Before the study commences, the research project undergoes ethical review by the ethics committee of the leading research institution, and each center's ethics committee records the study. In the event of a serious adverse event, the ethics committee of each center should promptly convene a meeting for review and inform the conclusions to other centers' ethics committees.

11.2 Informed Consent of Participants

Before enrolling any participant in this study, the investigator is responsible for providing a complete and comprehensive written explanation to the participant regarding the purpose, nature, procedures, and potential benefits and risks of this research. Participants should be informed of their right to withdraw from the study at any time. Prior to enrollment, each participant must be given written informed consent to ensure they understand the study and voluntarily agree to participate. Only after voluntarily signing the informed consent document can a participant be included in the clinical research. The informed consent form should be retained as one of the original documents of the clinical study for future reference.

11.3 Confidentiality

In all submitted documents to the sponsor, the identity of clinical research participants should only be referenced using their assigned patient numbers. Their names or hospital numbers should not be included. Researchers must securely store the participants' names, addresses, and corresponding patient numbers in a separate enrollment log. These enrollment logs must be strictly kept confidential by the researchers.

12. Study Flow Chart

	Enrollment	Treatment			Close-out
	Baseline (Visit 1)	Month 2 (Visit 2)	Month 4 (Visit 3)	Month 6 (Visit 4)	Month 12 (Visit 5)
**ENROLLMENT**					
Eligibility screen	X				
Informed consent	X				
General information	X				
Concomitant medication	X	X	X	X	
Symptoms and signs	X	X	X	X	
Allocation	X				
**INTERVENTIONS**					
QXJYG + GDT	X	X	X	X	
Placebo + GDT	X	X	X	X	
**ASSESSMENTS**					
Efficacy outcomes					
CT-FFR	X			X	
CCTA [a]	X			X	
SAQ	X	X	X	X	
hs-CRP	X			X	
MMP-9	X			X	
Blood lipids [a]	X			X	
Carotid artery ultrasound [b]	X			X	
Safety outcomes					
Composite events of bleeding		X	X	X	
Laboratory test	X	X		X	
Electrocardiogram	X	X		X	
Adverse events		X	X	X	
Endpoints					
MACE		X	X	X	X
Medication adherence		X	X	X	

Note: [a] CCTA includes the percentage of diameter and area stenosis, coronary artery calcification score, and Gensini score derived from the CT images. [b] Blood lipids include TC, TG, LDL-C, HDL-C, Apo A1, Apo B1, Lp(a). [c] Carotid ultrasound parameters include IMT (mm), carotid plaque length × thickness (mm) and carotid lumen stenosis (%). [d] Laboratory tests include complete blood count, coagulation function, liver and renal function, blood glucose, urine and stool routine.
