# Supplementary material for: Chinese herbal compound preparation Qing-Xin-Jie-Yu granules for intermediate coronary lesions in patients with stable coronary artery disease: Study protocol for a multicenter, randomized, double-blind, placebo-controlled trial
Source: PLoS One. 2024 Jul 16;19(7):e0307074. doi: 10.1371/journal.pone.0307074 (PMC11251585; doi:10.1371/journal.pone.0307074)
Supplement: S2 File — (PDF) [file pone.0307074.s005.pdf]

活血解毒干预冠状动脉临界病变的循证评价研究

临床研究方案

申办单位：中国中医科学院西苑医院

负责单位：中国中医科学院西苑医院

参加单位：中国中医科学院广安门医院

首都医科大学附属北京同仁医院

版本号 XYYY-V-1.0

版本日期 2021 年 12 月 26 日

1. 研究目的

通过多中心、随机、双盲、安慰剂对照临床研究,评价清心解瘀方干预稳定性冠心病患者冠脉临界病变的有效性和安全性。

2. 研究对象

2.1 诊断标准

1) 冠脉临界病变诊断标准：《2021 年 ACC/AHA/SCAI 冠脉血运重建标准》
2) 稳定性冠心病诊断标准 《2014 年 ACC/AHA/AATS/PCNA/SCAI/STS 重点更新稳定性缺血性心脏病患者诊断和治疗指南》；《2021 年 AHA/ACC/ASE/CHEST/SAEM/SCCT /SCMR 胸痛评估和诊断指南》。

2.2 纳入标准

1) 冠脉 CTA 检查确认至少一支主要冠状动脉管腔直径狭窄在 50%-70%之间的稳定性冠心病患者；
2) 心功能Ⅰ-Ⅱ级(NYHA 心功能分级)；
3) 18 岁≤年龄≤75 岁；
4) 受试者知情，自愿签署知情同意书。

2.3 排除标准

1) 既往行冠脉支架置入术、冠脉搭桥术或急性心肌梗死 3 个月内的患者；
2) 左主干直径狭窄≥50%，或三支主要血管均直径狭窄≥50%；
3) 病变血管全程弥漫性病变者；
4) 近 3 个月内血压剧烈波动，或难以控制的高血压（收缩压≥160mmHg、舒张压≥100mmHg）；
5) 静息心率>100 次/分，且难以控制；
6) 既往曾行其他心脏手术者，如瓣膜置换等；
7) 合并严重心、肝、肾功能不全，不适宜行冠脉造影及相关检查及治疗者；
8) 患有精神障碍性疾病者；
9) 患有肝炎、结核、艾滋病等传染性疾病者；

10) 过敏性体质者；

11) 孕妇及哺乳期妇女；

12) 预期寿命不足 1 年者；

13) 近 3 个月内参加过其他临床研究者。

2.4 剔除标准

1) “违反合法性”，即指病例选择违反了纳入/排除病例标准，本不应当进行随机化入组。

2) 受试者不配合随机化入组，或随机化入组后未服用任何试验药物，或用药极少（＜10%）。

3) 使用了禁用的药物以致有效性和安全性无法判定的病例。

4) 在随机化入组之后没有任何数据。

必须在盲态审核时由主要研究者、数据管理员、统计分析专家和申办者共同讨论后，判断该病例是否剔除。

2.5 脱落相关标准

1) 受试者病情持续加重，有可能发生危险事件，根据医生判断应该停止试验者。

2) 受试者发生了某些合并症、并发症或特殊生理变化，不适宜继续接受试验。

3) 试验中，受试者依从性差，或使用试验方案规定的禁用药品。

4) 发生不良事件或严重不良事件的受试者。

5) 泄盲病例。

2.5.1 <u>脱落病例判定</u>

所有填写过知情同意书并筛选合格进入试验的受试者，无论何时何故退出，只要没有完成规定的观察周期的受试者，均为脱落病例。脱落的病例应说明原因，如有基线药效数据，可将其最后一次的主要疗效指标结果转接为最终结果进行统计分析，其病例报告表（case report form, CRF）表应保留备查。

2.5.2 <u>脱落病例的处理</u>

当受试病例脱落后，研究者应采取电话、预约、随访、信件等方式与受试者保持联系，并询问理由、记录最后一次服药时间、完成所能完成的评估项目。随后填写

CRF 表。因过敏反应、不良反应、治疗无效而退出试验病例，研究者应根据受试者实际情况，采取相应的治疗措施和一定的经济补偿，以保障受试者的权益。

凡是入选病例，无论是否脱落，均应记录和保留 CRF 表，这也是进行 FAS 集分析（full analysis set）资料的原始记录。脱落病例无需另补，所有脱落病例均应将病例报告表交予临床负责单位汇总，并进行统计分析。

2.6 全面终止临床试验的标准

1) 试验中发现严重安全性问题。
2) 试验中发现药物治疗效果太差，甚至无效，不具有临床价值。
3) 发现临床试验方案有重大失误，难以评价药物疗效；或一项设计较好的方案，在实施中发生了重要偏差。
4) 申办者因经费原因、管理原因要求终止试验。

3. 试验设计

本试验采用多中心、随机、双盲、安慰剂对照设计。

3.1 多中心

本研究在 3 家中心同时开展：中国中医科学院西苑医院，中国中医科学院广安门医院，北京同仁医院。

3.2 随机

采用分层、区组随机的方法。运用 SAS 统计软件，按参加单位的病例分配数及随机比例生成《中心编码随机数字表》，所选择的区组(block)长度和随机初值种子参数等作为保密数据一起密封在盲底中。根据此随机数字表由与本试验无关的人员对药品进行编码。试验组与对照组按 1：1 分配。

3.3 对照

采用安慰剂对照。

3.4 样本量估算依据

样本量是根据 CT-FFR 值计算的。PROMISE 研究显示，临界病变患者的平均 CT-FFR 值为 0.77±0.1。临床研究表明，经中药干预后，冠心病患者的 CT-FFR 值为

0.92±0.16。因此，假设治疗后对照组的 CT-FFR 值为 0.77，干预组的 CT-FFR 值为 0.92，两组的标准差σ=0.13。NXT 研究结果表明，CT-FFR 每下降 0.05 个单位，与复合心血管事件发生率的增加独立相关。基于此，我们在本次优效性试验中设定界值 Δ=0.03。两组病例数按 1:1 的比例安排病例数，故 c=1。假设 I 类错误率α= 0.05（双侧检验），功效为 95%（II 类错误率β= 0.05），所以 u_α=1.96，u_β=1.64，n_1=n_2≈55。考虑最大脱落率为 10%，则总共需要 120 名患者。计算公式如下：

$$n_1=\left(\frac{1+c}{c}\right)\left[\frac{(u_\alpha+u_\beta)\,\sigma}{\mu_T-\mu_C-\Delta}\right]^2+\frac{1}{4}u_\alpha^2, \text{ and } n_2=cn_1$$

3.5 盲法

本次试验采用二级盲法设计，第一级为各病例号所对应的组别（如 A、B 组），第二级为各组所对应的处理（试验组、对照组）。

3.5.1 编盲、盲底保存

用 SAS 统计软件包按分层随机方法产生随机数，根据此随机数对药品进行编盲包装。试验使用双盲双模拟技术，所有的研究药物和对照药均应采用相同的包装，并按照随机数字进行分装编盲。对试验药和对照药进行统一包装，同时保证真药和模拟药从外观上无差异。设盲工作由临床研究负责单位负责人与申办者及统计人员共同完成。盲底一式二份，密封后由主要研究单位和申办者保存，试验期间盲底不能拆阅。

3.5.2 紧急揭盲

在药物包装编码的同时，为每个药品编号设置一份应急信件，信件内密封相应编号药物所属组别。遇医学紧急情况方可拆阅应急信件（紧急揭盲）。在下列情况可以考虑进行揭盲，包含但不限于：

a) 当患者发生严重的不良反应/不良事件；

b) 当患者发生严重的并发症；

c) 症状恶化、必须采取紧急措施者；

紧急情况下确需揭盲时，由研究者请示中心负责人，经中心负责人签字同意后可拆阅应急破盲信件，紧急揭盲时，应详细记录揭盲原因、时间、地点，并签字。揭盲后 24 小时内通知临床研究组长单位以及申办者相关人员并解释揭盲原因。由于疗效的原因而退出的病例，不得揭盲。揭盲病例资料应保存完整。

3.5.3 揭盲规定

本研究采用二次揭盲法，当 CRF 全部录入数据库，并经答疑、核查、盲态审核并确认最终的统计计划书后，数据库将被锁定。此时进行第一次揭盲，由本次研究的主要研究者会同统计人员一起揭盲，即将各药物编号所对应的组别（A 组或 B 组）的盲底揭晓，以便对全部数据进行分组后的统计分析。分析完成后在临床研究总结会上由主要研究者进行第二次揭盲，揭晓 A 组、B 组所对应的治疗措施。所有揭盲过程均应有记录。

4. 试验干预措施

4.1 试验用药

所有入选患者随机分为试验组和对照组。两组均按照冠心病诊疗指南进行西医常规治疗。在此基础上，试验组选择临床疗效确切的活血解毒中药清心解瘀颗粒治疗；对照组采用安慰剂治疗。疗程 6 个月，随访 12 月。

4.2 药物包装

每个受试者的药物为一个大包装，包含 3 个中包装（每个访视期为一个中包装）及 2 天备用药量的一个方便袋。一个访视疗程药量为一个中包装，2 天的备用药量单独用塑料方便袋包装后存放于大包装纸盒内。大、中包装需粘贴带有药物编号的标签。

4.3 药品管理

药品应统一管理，统一保存。课题负责单位按照临床试验要求提供试验药品及对照药品，研究者建立临床试验用药品管理记录卡，检查药品的数量、质量及药品编码是否正确。在药品发放过程中，由专人清点药物，记录使用情况，保证按照药物编码正确发放药物。试验结束后，由专人进行剩余药品的回收及销毁。

4.4 合并用药规定

1) 试验期间不可同时服用其它治疗冠心病、动脉粥样硬化、高脂血症的中成药。
2) 合并其它疾病所必须继续服用的药物，可继续服用，所用的药物或其他治疗方法必须在格式病案记录药名（或其它疗法名）、用量、使用次数和时间等，以便总结时加以分析和报告。

5. 观察项目及指标

5.1 一般资料

1) 人口统计学指标：出生年月、性别、身高、体重、民族、职业、BMI、血压；

2) 一般临床资料：现病史、既往史、吸烟史、饮酒史、用药史。

既往史重点询问：早发心血管病家族史（一级亲属，男 55 岁、女 65 岁以前发病）、脑血管病史、周围血管病病史、糖尿病病史、高血压病史、血脂异常病史。

5.2 疗效指标

5.2.1 主要疗效指标

基于定量 CTA 评估的冠脉血流储备分数（CT-FFR）

5.2.2 次要疗效指标

1) 冠脉病变影像学评估：冠脉直径狭窄百分比（% DS）；冠脉钙化积分、冠脉 Gensini 评分；

2) 西雅图心绞痛量表评分（SAQ）；

3) 血清炎症因子：高敏 C 反应蛋白（hs-CRP）；基质金属蛋白酶 9(MMP-9)；

4) 血脂七项：总胆固醇（TC）、甘油三酯（TG）、低密度脂蛋白胆固醇（LDL-C）、高密度脂脂蛋白胆固醇（HDL-C）、脂蛋白（a）[Lp(a)]、载脂蛋白 AⅠ(Apo AI)、载脂蛋白 B(Apo B)；

5) 颈动脉超声；颈动脉内中膜厚度（IMT)（mm）、颈动脉斑块长度×厚度（纵切面）（mm）、颈动脉管腔狭窄程度（%）

5.3 安全性指标

5.3.1 主要安全性指标

复合出血事件：根据 BARC 联盟定义的 2、3 或 5 型出血事件。

5.3.2 次要安全性指标

1) 凝血四项：凝血酶原时间（PT）、凝血酶时间（TT）、国际标准化比值（INR）、活化部分凝血活酶时间（APTT）；

2) 血常规：红细胞（RBC）、血红蛋白（HB）、白细胞（WBC）、中性粒细胞比例（NEUT%）、血小板（PLT）；

3) 尿常规：包括尿蛋白（PRO）、尿糖（GLU）、尿红细胞、尿白细胞。

4) 大便常规：包括白细胞、便潜血等；

5) 肝功能：谷丙转氨酶（ALT）、谷草转氨酶（AST）；

6) 肾功能：血尿素氮（BUN）、血尿酸（UA）、血清肌酐（Scr）

7) 空腹血糖（FBG）；

8) 心电图

5.4 终点指标

主要心血管事件（MACE 事件）：包括非致命性心肌梗死、心源性死亡、血运重建治疗(包括介入治疗、冠脉搭桥术)。

6. 随访

治疗 6 个月，随访 12 个月。随访时间点为：治疗前（访视 1）、治疗后 2 个月（访视 2）、治疗后 4 个月（访视 3）、治疗后 6 个月（访视 4）和治疗后 12 个月（访视 5）。

中止病例退出试验时的流程

a) 回收剩余药品及包装，并记录；

b) 问诊（询问任何不良事件及其他相关信息）；

c) 体格检查；

d) 完成疗效和安全性评价；

e) 记录中止的原因；

7. 不良事件

7.1 不良事件的记录

研究者发现不良事件后，应立即记载 CRF 表中，如不良事件属于严重不良事件、新的药品不良反应或者药品严重不良反应，需按照相应流程进行报告。

7.2 不良事件的判断

7.2.1 严重程度判断

轻度	受试者可忍受，不影响治疗、不需要特别处理，对受试者康复无影响。
中度	受试者难以忍受，需要撤药或做特殊处理，对受试者康复有直接影响。
重度	危及受试者生命，致死或致残，需立即撤药或作紧急处理。

7.2.2 分析方法

根据我国《药品不良反应报告和监测管理办法》，使用的分析方法主要遵循以下五条原则。

a)	用药与不良反应/事件的出现有无合理的时间关系？有□无□
b)	反应是否符合该药已知的不良反应类型？是□否□不明□
c)	停药或减量后，反应/事件是否消失或减轻？是□否□不明□未停药或未减量□
d)	再次使用可疑药品后是否再次出现同样反应/事件？是□否□不明□未再使用□
e)	反应/事件是否可用并用药的作用、患者病情的进展、其他治疗的影响来解释？是□否□不明□

7.2.3 关联性评价

依据以上不良反应/事件分析的五条原则，将关联性评价分为：肯定、很可能、可能、可能无关、待评价、无法评价 6 级。其中"肯定、很可能、可能"3 级为药品不良反应。

	1	2	3	4	5
肯定	+	+	+	+	−
很可能	+	+	+	?	−
可能	+	±	±?	?	±?
可能无关	−	−	±?	?	±?
待评价	需要补充材料才能评价				
无法评价	评价的必须资料无法获得				

注：＋表示肯定；－表示否定；±表示难以肯定或否定；？表示不明

7.3 不良事件转归

死亡	导致生命结束（须收集死亡原因和死亡时间）。
未痊愈/未缓解	AE 经治疗后，症状未改善/未恢复。
痊愈	AE 经治疗后，症状完全消失，且没有后遗症状。
症状消失但有后遗症	AE 经治疗后，症状已消失但留有后遗症（记录时应注明后遗症的名称或表现）。
缓解	AE 经治疗后，症状改善。
不详	情况不明，未进行跟踪观察、记录，或受试者拒绝。

7.4 不良事件的报告

一般不良反应只需在原始病历及病例报告表中记录，无需特别报告。

严重不良事件应该在获知该事件的 24 小时内，填写严重不良事件报告表并向国家药监局、研究中心所在省/市药品监督管理局、研究中心的伦理委员会及申办者报告，并保留相应的书面记录。

8. 临床数据管理

8.1 电子化数据管理

本研究采用采用 SQLSever2000 建立数据库，进行数据录入管理。专人负责数据管理和保密。

8.2 数据管理计划

1) 数据管理计划书由项目数据管理员(下称：PL)撰写。
2) 在研究启动之前，数据管理室主任(下称：DMD)对数据管理计划书进行审阅，以确保计划书包括了所要求的步骤和信息。
3) ⑶数据计划书必须得到申办方的批准。

8.3 电子病例报告表的设计与建立

1) 设置 eCRF 页面：PL 根据纸质 CRF 或研究病历建立 eCRF 的页面
2) eCRF 页面测试：PL 设置页面完成后，交与本项目无关的另一名项目数据管理员(下称：PL2) 进行测试，测试通过后，向 DMD 提交 eCRF 发布申请。

8.4 数据录入

1) 每个研究中心由 2 名护士担任研究助理(CRA)进行数据录入工作；
2) 登陆西苑医院药物临床试验数据管理系统，按随访时点录入研究数据；
3) 独立双份录入；
4) eCRF 不作为原始记录，其内容源于“研究病历”。

8.5 数据核查和清理

8.5.1 数据核查计划

由 PL 根据 eCRF 制定数据核查计划(下称：DVP)。DVP 应对方案中规定的主要和次要有效性指标、关键的安全性指标进行充分的核查以确保这些数据的正确性和完整性。数据核查应在未知试验分组情况下进行。对任何数据差异或问题生成的数据质疑表（下称：DCF）。

8.5.2 数据核查方式

1) 源数据现场核查(SDV)：监查员在各中心研究现场登陆数据管理系统的数据监查端，100% 的核对电子 CRF 数据与源数据的一致性。电子 CRF 与研究病历数据不一致时，在线发出疑问。
2) 系统自动逻辑核查：PL 对 DVP 进行分析，选出可以进行系统自动核查的部分，编写程序并加载到 eCRF 页面中。录入员在录入数据时，计算机会自动对这些数据进行核查，以提高数据录入的质量及数据管理的效率。
3) 人工核查：对无法使用计算机自动核查的数据，由CRA进行在线人工审核，如发现疑问可直接点击疑问发送。

8.6 疑问的处理

1) 发行：由数据录入员定期登录系统，下载本机构的疑问生成 DCF。
2) 答疑：PL 或监查员在进行数据清理期间发出的所有数据疑问(除 SDV 核查疑问)，都应该以 DCF 的方式要求研究者对疑问数据进行书面回答并签字,数据可只在 DCF 上修改，DCF 作为源文件与研究病历等原始数据一同保存。
3) 在线更新：数据录入员将 DCF 上的答疑内容进行在线数据更新
4) 解决：PL 或监查员应对更新的数据再次进行审核，直到所有疑问数据解决为止。

8.7 数据审核会议

PL 形成数据管理报告后，通知申办者召开数据审核会议。由 PL 报告试验过程中的数据管理情况，并由申办方、主要研究者、数据管理人员和统计师对未解决的数据问题进行最终审核，按照统计计划书，共同讨论数据集划分，核查严重不良事件报告与处理情况记录等。

在对所有数据疑问及数据集划分达成共识，根据数据审核意见更新数据后，数据管理员将在获得锁定批准文件后锁定数据库，并将干净的数据库移交统计方。

8.8 数据库的锁定与解锁

8.8.1 数据库锁定

数据锁定应满足以下前提条件：

- **所有数据已经正确录入数据库。**
- **所有的数据质疑表已经解答并进入数据库。**
- **研究者已审核所有 eCRF。**
- **已完成医学编码。**
- **已完成最终的数据和逻辑性和一致性验证结果审查。**
- **已完成最终的明显错误或异常的审查。**
- **根据标准操作程序更新并保存了所有试验相关文档。**

PL 确认试验符合以上条件后，提出书面锁定申请，并由试验相关人员（DMD、生物统计师、临床监查员代表、主要研究者）签名及签署日期，一旦获得数据库锁定的书面批准文件，由 PL 锁定数据库，并进行书面记录。

8.8.2 数据库解锁

数据库锁定后，如发现错误数据，由 pl 提出书面解锁申请，并附数据审核意见，数据审核意见应由数据管理室主任、生物统计师、申办者、主要研究者共同签署。由 PL 解锁数据库并根据数据审核意见修订数据，所有修订数据均具备稽查痕迹。数据库再次锁定遵循和数据库首次锁定同样的过程。

9. 统计分析

统计检验均采用双侧检验，$P \le 0.05$ 将被认为所检验的差别有统计意义。

9.1 统计分析人群

1) 意向性分析原则(ITT, intent-to-treat)：指所有表达接受治疗意向并签署知情同意书的受试者均纳入分析。
2) 全分析集(FAS, full analysis set)：指签署知情同意书，并且至少服用过 1 次试验药物且获得相应疗效指标记录的受试者。基线数据分析以 FAS 为对象，疗效分析以 FAS 分析结果为主要依据。
3) 符合方案集(PPS, pre-protocol set)：指符合纳入标准、不符合排除标准、完成治疗方案的受试者。PP 分析是指对符合试验方案、依从性好的受试者进行分析。PP 分析侧重于考察与 FAS 分析结果的一致性，以判断结果的稳定性。

4) 安全评价人群(SS)：有安全性指标记录的受试者。安全性指标的缺失值不做估计。

9.2 统计分析方法

9.2.1 一般考虑

1) 所有统计检验均采用双侧检验，P 值小于或等于 0.05 将被认为所检验的差异有统计意义。
2) 统计描述：连续变量将表示为平均值±标准差、中位数和四分位距。分类变量将表示为频率和百分比（%），如有必要，将给出平均秩次。
3) 离群值处理：离群值根据临床专业人员的判断结合统计学准则，决定取舍。
4) 缺失值估计：FAS 中，疗效性指标的缺失数据将采用多重插补法进行填补。PPS 和 SS 中的缺失数据不做填补，利用所获得的实际数据进行分析。

9.2.2 疗效分析

1) 主要疗效指标：CT-FFR。描述基线、疗后 6 个月病变血管的 CT-FFR 值、及疗后 6 个月相对基线的差值，多支病变时选取狭窄程度最重一支的测量值。组间比较采用线性混合模型，因变量为治疗 6 个月相对于基线的 CT-FFR 差值，固定因素是分组、中心，随机因素是受试者，协变量包括基线 CT-FFR 值、年龄、性别、BMI、SBP、DBP、LDL-C、FBG、吸烟状况、饮酒状况、是否存在早发心血管病家族史、脑血管病史、周围血管病病史、糖尿病病史、高血压病史、血脂异常病史、是否合并使用 PCSK-9 抑制剂，采用最小二乘法估计均值差和 95% 可信区间。

2) 次要疗效指标

   ① 冠脉病变影像学评估指标：%DS 选取最狭窄部位的测量值作为结果；冠脉钙化积分和 Gensini 评分选取总积分作为结果。描述基线、疗后 6 个月的%DS、冠脉钙化积分和 Gensini 评分，及疗后 6 个月相对基线的差值。组间比较采用线性混合模型，因变量为治疗 6 个月相对于基线的差值，固定效应是分组、中心，随机效应是受试者，基线值作为协变量，采用最小二乘法估计均值差和 95% 可信区间。

   ② 西雅图心绞痛量表评分（SAQ）：描述基线、疗后 2、4、6 个月的 SAQ 评分，及疗后 2、4、6 个月相对基线的差值。组间比较采用重复测量的混合模型，因变量为治疗后 2、4、6 个月的 SAQ 评分较基线的下降值，疗后 6 个月为主要

评估时间点，固定效应为分组、中心，随机效应是受试者，基线 SAQ 评分值、访视、以及基线和访视的交互项、治疗分组和访视的交互项作为协变量，估算组间的最小二乘均值差和 95% 可信区间。

③ 血清炎症因子、血脂、颈动脉超声评估指标：描述基线、疗后 6 个月的测量值，及疗后 6 个月相对基线的差值。采用线性混合模型，因变量为治疗 6 个月相对于基线的差值，固定效应为分组、中心，随机效应是受试者，基线值作为协变量，采用最小二乘法估计均值差和 95% 可信区间。

3) 终点指标：MACE 事件。描述基线至随访 1 年期间 MACE 事件发生率。采用 Cox 比例风险模型，因变量为 MACE 事件发生状态，生存时间变量为基线到 MACE 事件发生的时间或随访结束时间，主要自变量为分组，协变量包括年龄、性别、BMI、SBP、DBP、LDL-C、FBG、吸烟状况、饮酒状况、是否存在早发心血管病家族史、脑血管病史、周围血管病病史、糖尿病病史、高血压病史、血脂异常病史、是否合并使用 PCSK-9 抑制剂、以及研究中心，采用部分似然法估计风险比及其 95 %置信区间。

4) 亚组分析：考虑到合并糖尿病和合并使用 PCSK-9 对药物疗效的显著影响，分别对冠心病合并糖尿病的患者和合并使用 PCSK-9 的冠心病患者进行主要疗效指标和终点事件的亚组分析。

9.2.3 安全性分析

1) 主要安全性指标：列出复合出血事件的详细列表。
2) 次要安全性指标：统计描述实验室安全性指标疗前疗后的测量值，列出实验室指标和心电图用药前后交叉表。并判断其正常/异常转变的临床意义。
3) 不良事件：列出不良事件/反应的详细列表，并判断与试验药物的关系。

9.2.4 依从性分析

采用实际服药量和用药持续时间来描述服药依从性。

9.3 统计软件

采用 SAS 9.3 软件分析。

10. 质量控制

10.1 数据质量保障

1) 大部分化验指标尽量在同一个实验室用相同的仪器与批次相同的试剂进行检测，对于在两个或两个以上实验室进行的相同化验指标，也要求合用同一型号的仪器、同批次的试剂、同样的操作流程，并定期进行试验室测量结果的比对。
2) 对各主要设备仪器进行定期的质量控制检测。

10.2 质量控制方法

1) 制定详细的工作手册，对研究者、检验检测操作人员进行统一培训。所有仪器操作员与实验室化验员均要进行统一的培训与考核，不同操作员或化验员之前的一致性使用 Kappa 统计检验，要求 Kappa 值要达到 0.85 以上。
2) 成立质量控制小组，由监察员组成，定期检查各中心原始资料，并核对原始资料与录入资料的一致性。

11. 研究伦理学要求

11.1 伦理学审核

本临床研究将遵循赫尔辛基宣言和中国有关临床研究研究法规。本研究项目在研究开始前由课题组长单位伦理委员会审查，各中心伦理委员会备案。如果发生严重不良事件时，各中心伦理委员会应及时召开会议进行审查，并将审查结论通报其他中心伦理委员会。

11.2 受试者知情同意

每一位受试者入选本研究前，研究医师有责任以书面文字形式，向其或其指定代表完整、全面地介绍本研究的目的、性质、程序和可能的受益及风险。应让受试者知道他们有权随时退出本项研究。入选前必须给每位受试者一份书面的知情同意书使受试者了解后表示同意。并自愿签署知情同意书后，方可入选进行临床研究。知情同意书应作为临床研究的原始资料之一，保存备查。

11.3 信息保密性

研究人员必须保证维护临床研究受试者的隐私。在所有提交申办方的文件资料中，只能以临床研究的患者号来确定临床研究受试者的身份，而不能注明受试者的姓名及其住院号。研究者必须妥善保管有关临床研究受试者的姓名地址和与临床研究患者号相对应的入组表。这些入组表由研究者严格保密保存。

12.临床研究流程图

	基线	治疗			随访
	治疗前（访视 1）	治疗后 2 个月（访视 2）	治疗后 4 个月（访视 3）	治疗后 6 个月（访视 4）	治疗后 12 个月（访视 6）
**招募**					
确定纳排病例	X				
签署知情同意书	X				
填写一般资料	X				
合并用药记录	X	X	X	X	
症状与体征	X	X	X	X	
随机分配	X				
**干预**					
清心解瘀方	X	X	X	X	
安慰剂	X	X	X	X	
**评估**					
**疗效性指标**					
CT 血流储备分数	X			X	
冠脉 CTA [a]	X			X	
西雅图心绞痛量表	X	X	X	X	
hs-CRP	X			X	
MMP-9	X			X	
血脂七项 [b]	X			X	
颈动脉超声 [c]	X			X	
**安全性指标**					
复合出血事件		X	X	X	
实验室检测 [d]	X	X		X	
心电图	X	X		X	
不良事件		X	X	X	
**结局指标**					
MACE 事件		X	X	X	X
**药物依从性**		X	X	X	

注：[a] 冠脉 CTA 包括直径狭窄百分比、面积狭窄百分比、冠脉钙化积分、Gensini 评分。[b] 血脂包括总胆固醇、甘油三酯、低密度脂蛋白胆固醇、高密度脂蛋白胆固醇、载脂蛋白 AI、载脂蛋白 B、脂蛋白（a）。[c] 颈动脉超声参数包括颈动脉内中莫厚度（IMT）（mm）、颈动脉斑块长度×厚度（mm）和颈动脉管腔狭窄度（%）。[d] 实验室检查包括全血细胞计数、凝血功能、肝肾功能、血糖、尿常规和便常规。
