# Supplementary material for: Chinese herbal compound preparation Qing-Xin-Jie-Yu granules for intermediate coronary lesions in patients with stable coronary artery disease: Study protocol for a multicenter, randomized, double-blind, placebo-controlled trial
Source: PLoS One. 2024 Jul 16;19(7):e0307074. doi: 10.1371/journal.pone.0307074 (PMC11251585; doi:10.1371/journal.pone.0307074)
Supplement: S4 File — (PDF) [file pone.0307074.s007.pdf]

华润三九现代中药制药有限公司

成品检验报告书

样品编号: SA220619009	报告书编码: P20221223001S
检品名称: 黄芪（蒙古黄芪）配方颗粒	批 号: 2206001S
生产单位: 颗粒剂厂外车间（深圳）	包装规格: 250g/瓶
包 装: 口服固体药用高密度聚乙烯瓶	数 量: 2001 瓶
检验目的: 放行	检验项目: 全检
收验日期: 2022.06.19	保质期: 36 个月
报告日期: 2022.12.23	检验依据: 国家药品标准 YBZ-PFKL-2021065

检验结果:

检验项目	标准规定	检验数据	项目结论
[性状]	本品为灰黄色至棕黄色的颗粒；气微，味微甜、微苦	本品为棕黄色的颗粒；气微，味微甜、微苦。	符合规定
[鉴别]			
薄层鉴别 1	应检出黄芪甲苷	检出黄芪甲苷	符合规定
薄层鉴别 2	应检出黄芪（蒙古黄芪）	检出黄芪（蒙古黄芪）	符合规定
特征图谱	应符合规定	符合规定	符合规定
[检查]			
水分	不得过 8.0%	5.9%	符合规定
溶化性	应符合规定	符合规定	符合规定
粒度	不能通过一号筛与能通过五号筛的颗粒和粉末总和，不得超过 15%。	4.1%	符合规定
装量	应符合规定	符合规定	符合规定
重金属及有害元素	铅不得过 5mg/kg<br>镉不得过 1 mg/kg<br>砷不得过 2mg/kg<br>汞不得过 0. 2mg/kg<br>铜不得过 20mg/kg	铅 0.1mg/kg；<br>镉 0.01mg/kg；<br>砷 0.5mg/kg<br>汞 0mg/kg；<br>铜 3mg/kg；	符合规定
其他有机氯类农药残留量	含五氯硝基苯不得过 0.1mg/kg	未检出	符合规定
浸出物	不得少于 18.0%	38.5%	符合规定
[含量测定]			
毛蕊异黄酮葡萄糖苷	每 1g 应为 0.50mg~2.00mg	0.68mg	符合规定
黄芪甲苷	每 1g 应为 1.20mg~3.50mg	3.12mg	符合规定
[微生物限度]			
需氧菌总数	不得过 10^3cfu/g	<10cfu/g	符合规定
霉菌和酵母菌总数	不得过 10^2cfu/g	10cfu/g	符合规定
大肠埃希菌	每 1g 不得检出	每 1g 未检出	符合规定

检验结论：本品按国家药品标准 YBZ-PFKL-2021065 检验，结果符合规定 。

负责人: [signature]　　　　复核人: [signature]

China Resources Sanjiu Modern Chinese Medicine Pharmaceutical Co., Ltd
Finished product inspection report

Sample number: SA220619009	Report Code: P20221223001S
Sample name: Astragalus (Mongolian Astragalus) formula granules	Batch number: 2206001S
Production unit: granule off-site workshop (Shenzhen)	Packaging specification: 250g/bottle
Packaging: oral solid medicine high-density polyethylene bottle	Quantity: 2001 bottles
Inspection purpose: release	Inspection items: full inspection
Receipt date: June 19, 2022	Shelf life: 36 months
Report date: December 23, 2022	Inspection basis: National Drug Standard YBZ-PFKL-2021065

Inspection results:

Inspection items	Standard Requirement	Inspection data	Item conclusion
[Appearance]	The product should be grayish-yellow to brownish-yellow granules, with slight odor, mildly sweet and bitter taste	The product is grayish-yellow to brownish-yellow granules, with slight odor, mildly sweet and bitter taste.	Compliance
[Identification]			
Thin-layer identification 1	Astragaloside IV should be detected	Astragaloside IV is detected	Compliance
Thin-layer identification 2	Astragalus (Mongolian Astragalus) should be detected	Astragalus (Mongolian Astragalus) is detected	Compliance
Characteristic chromatogram	Should comply	Compliance	Compliance
[Inspection]			
Water content	Not exceed 8.0%	5.9%	Compliance
Solubility	Should comply	Compliance	Compliance
Particle size	The sum of particles and powders that cannot pass through a No. 1 sieve and those that can pass through a No. 5 sieve should not exceed 15%.	4.1%	Compliance
Volume	Should comply	Compliance	Compliance
Heavy metals and harmful elements	Lead: not exceed 5mg/kg<br>Cadmium: not exceed 1mg/kg<br>Arsenic: not exceed 2mg/kg<br>Mercury: not exceed 0.2mg/kg<br>Copper: not exceed 20mg/kg	Lead: 0.1mg/kg<br>Cadmium: 0.01mg/kg<br>Arsenic: 0.5mg/kg<br>Mercury: 0mg/kg<br>Copper: 3mg/kg	Compliance
Other organochlorine pesticide residues	Contains pentachloronitrobenzene: not exceed 0.1mg/kg.	Not detected	Compliance
Leachate	Not less than 18.0%	38.5%	Compliance
[Content determination]			
Calycosin-7-glucoside	0.50mg to 2.00mg per 1g	0.68mg	Compliance
Astragaloside IV	1.20mg to 3.50mg per 1g	3.12mg	Compliance
[Microbial Limits]			
Total aerobic microbial count	Not exceed 10^3 cfu/g	<10cfu/g	Compliance
Total mold and yeast count	Not exceed 10^2 cfu/g	10cfu/g	Compliance
Escherichia coli	Should not be detected per 1g	Not be detected per 1g	Compliance

Conclusion: according to the national drug standard YBZ-PFKL-2021065, inspection results of the product are in compliance with the requirements.

Responsible person: Reviewer:

华润三九现代中药制药有限公司

成品检验报告书

样品编号: SA220620011	报告书编码: P20220707014S
检品名称: 丹参配方颗粒	批　号: 2206011S
生产单位: 颗粒剂厂外车间（深圳）	包装规格: 250g/瓶
包　装: 口服固体药用高密度聚乙烯瓶	数　量: 1200 瓶
检验目的: 放行	检验项目: 全检
收验日期: 2022.06.20	产品有效期: 36 个月
报告日期: 2022.07.07	检验依据: 国家药品标准 YBZ-PFKL-2021035

检验结果:

检验项目	标准规定	检验数据	项目结论
[性状]	本品为黄棕色至棕色的颗粒；气微，味微苦、涩。	本品为棕色的颗粒；气微，味微苦、涩。	符合规定
[鉴别]			
薄层鉴别	应检出丹参、丹酚酸 B	检出丹参、丹酚酸 B	符合规定
[指纹图谱]	应符合规定	符合规定	符合规定
[检查]			
水分	应不得过 8.0%	4.0%	符合规定
溶化性	应符合规定	符合规定	符合规定
粒度	不能通过一号筛与能通过五号筛的颗粒和粉末总和，不得超过 15%	9.5%	符合规定
装量	应符合规定	符合规定	符合规定
重金属及有害元素	铜不得过 20mg/kg<br>砷不得过 2mg/kg<br>镉不得过 1mg/kg<br>汞不得过 0.2mg/kg<br>铅不得过 5mg/kg	铜 1mg/kg<br>砷 1mg/kg<br>镉 0.01mg/kg<br>汞 0mg/kg<br>铅 0.1mg/kg	符合规定
[浸出物]	不得少于 9.3%	19.5%	符合规定
[含量测定]			
丹酚酸 B	每 1g 应含 28.0mg~59.0mg	33.5mg	符合规定
[微生物限度]			
需氧菌总数	不得过 10^3cfu/g	<100cfu/g	符合规定
霉菌和酵母菌总数	不得过 10^2cfu/g	<10cfu/g	符合规定
大肠埃希菌	每 1g 不得检出	每 1g 未检出	符合规定

检验结论：本品按国家药品标准 YBZ-PFKL-2021035 检验，结果（~~不~~）符合规定 。

负责人:　　　　　　　　　　　　复核人:

China Resources Sanjiu Modern Chinese Medicine Pharmaceutical Co., Ltd
Finished product inspection report

Sample number: SA220620011	Report Code: P20220707014S
Sample name: Salvia miltiorrhiza formula granules	Batch number: 2206011S
Production unit: granule off-site workshop (Shenzhen)	Packaging specification: 250g/bottle
Packaging: oral solid medicine high-density polyethylene bottle	Quantity: 1200 bottles
Inspection purpose: release	Inspection items: full inspection
Receipt date: June 20, 2022	Shelf life: 36 months
Report date: July 07, 2022	Inspection basis: National Drug Standard YBZ-PFKL-2021035

Inspection results:

Inspection items	Standard Requirement	Inspection data	Item conclusion
[Appearance]	The product should be yellow-brown to brown granules, with slight odor, mildly bitter and astringent taste.	The product is yellow-brown to brown granules, with slight odor, mildly bitter and astringent taste.	Compliance
[Identification]			
Thin-layer identification	Salvia miltiorrhiza and salvianolic acid B should be detected	Salvia miltiorrhiza and salvianolic acid B is detected	Compliance
[Fingerprint chromatogram]	Should comply	Compliance	Compliance
[Inspection]			
Water content	Not exceed 8.0%	4.0%	Compliance
Solubility	Should comply	Compliance	Compliance
Particle size	The sum of particles and powders that cannot pass through a No.1 sieve and those that can pass through a No.5 sieve should not exceed 15%.	9.5%	Compliance
Volume	Should comply	Compliance	Compliance
Heavy metals and harmful elements	Copper: not exceed 20mg/kg<br>Arsenic: not exceed 2mg/kg<br>Cadmium: not exceed 1mg/kg<br>Mercury: not exceed 0.2mg/kg<br>Lead: not exceed 5mg/kg.	Copper: 1mg/kg<br>Arsenic: 1mg/kg<br>Cadmium: 0.01mg/kg<br>Mercury: 0mg/kg<br>Lead: 0.1mg/kg	Compliance
[Leachate]	Not less than 9.3%	19.5%	Compliance
[Content determination]			
Salvianolic acid B	28.0mg to 59.0mg per 1g	33.5mg	Compliance
[Microbial Limits]			
Total aerobic microbial count	Not exceed 10^3 cfu/g	<100cfu/g	Compliance
Total mold and yeast count	Not exceed 10^2 cfu/g	<10cfu/g	Compliance
Escherichia coli	Should not be detected per 1g	Not be detected per 1g	Compliance

Conclusion: according to the national drug standard YBZ-PFKL-2021035, inspection results of the product are in compliance with the requirements.

Responsible person: Reviewer:

华润三九现代中药制药有限公司

成品检验报告书

样品编号：BA221212018	报告书编号：P20221229020B
检品名称：川芎配方颗粒	批号： 2212002C
生产单位：华润三九现代中药制药有限公司厂外车间（淮北）	包装规格：250g/瓶
包装： 口服固体药用高密度聚乙烯瓶	数量： 1971 瓶
检验目的： 成品放行	检验项目： 全检
收验日期： 2022.12.12	保质期：36 个月
报告日期： 2022.12.29	检验依据：国家药品标准 YBZ-PFKL-2021029

检验结果：

检验项目	标准规定	检验数据	检验结论
[性状]	本品应为淡黄色至黄棕色的颗粒；气微香，味微苦、辛。	本品为黄棕色的颗粒；气微香，味微苦、辛。	符合规定
[鉴别]	应检出川芎<br>应检出阿魏酸	检出川芎<br>检出阿魏酸	符合规定
[检查]			
1.水分	应不得过 8.0%	2.4%	符合规定
2.溶化性	应符合规定	符合规定	符合规定
3.粒度	不能通过一号筛和能通过五号筛的颗粒与粉末的总和应不大于 15%	2.0%	符合规定
4.装量	应符合规定	符合规定	符合规定
[浸出物]	应不得少于 15.0%	47.0%	符合规定
[特征图谱]	应符合规定	符合规定	符合规定
[含量测定]	本品每 1g 含阿魏酸（$C_{10}H_{10}O_4$）应为 1.5mg~4.5mg。	2.3mg/g	符合规定
[微生物限度]			
需氧菌数	应≤10^3cfu/g	160cfu/g	符合规定
霉菌和酵母菌数	应≤10^2cfu/g	＜10cfu/g	符合规定
大肠埃希菌	每 1g 不得检出	每 1g 未检出	符合规定

检验结论：本品按 国家药品标准 YBZ-PFKL-2021029 标准检验,结果符合规定。

负责人：马湘云　　　　复核人：[illegible]

China Resources Sanjiu Modern Chinese Medicine Pharmaceutical Co., Ltd
Finished product inspection report

Sample number: BA221212018	Report Code: P20221229020B
Sample name: Ligusticum chuanxiong Hort formula granules	Batch number: 2212002C
Production unit: off-sit workshop of CR Sanjiu Modern Chinese Medicine Pharmaceutical Co., Ltd (Huaibei)	Packaging specification: 250g/bottle
Packaging: oral solid medicine high-density polyethylene bottle	Quantity: 1971 bottles
Inspection purpose: release	Inspection items: full inspection
Receipt date: December 12, 2022	Shelf life: 36 months
Report date: December 29, 2022	Inspection basis: National Drug Standard YBZ-PFKL-2021029

Inspection results:

Inspection items	Standard Requirement	Inspection data	Item conclusion
[Appearance]	The product should be light yellow to yellow-brown granules, with slightly fragrant odor, slightly bitter and pungent taste.	The product is yellow-brown to brown granules, with slight odor, mildly bitter and astringent taste.	Compliance
[Identification]	Ligusticum chuanxiong Hort should be detected<br>Ferulic acid should be detected	Ligusticum chuanxiong Hort is detected<br>Ferulic acid is detected	Compliance
[Inspection]			
Water content	Not exceed 8.0%	2.4%	Compliance
Solubility	Should comply	Compliance	Compliance
Particle size	The sum of particles and powders that cannot pass through a No.1 sieve and those that can pass through a No.5 sieve should not exceed 15%.	2.0%	Compliance
Volume	Should comply	Compliance	Compliance
[Leachate]	Not less than 15.0%	47.0%	Compliance
[Characteristic chromatogram]	Should comply	Compliance	Compliance
[Content determination]	The product should contain 1.5mg~4. 5mg ferulic acid ($C_{10}H_{10}O_4$) per 1g.	2.3mg/g	Compliance
[Microbial Limits]			
Total aerobic microbial count	Not exceed 10^3 cfu/g	160cfu/g	Compliance
Total mold and yeast count	Not exceed 10^2 cfu/g	<10cfu/g	Compliance
Escherichia coli	Should not be detected per 1g	Not be detected per 1g	Compliance

Conclusion: according to the national drug standard YBZ-PFKL-2021029, inspection results of the product are in compliance with the requirements.

Responsible person: Reviewer:

华润三九现代中药制药有限公司

成品检验报告书

样品编号：BA221216008	报告书编号：P20221228001B
检品名称：广藿香配方颗粒	批号：2212006C
生产单位：华润三九现代中药制药有限公司广外车间（淮北）	包装规格：250g/瓶
包装：口服固体药用高密度聚乙烯瓶	数量：1777 瓶
检验目的：成品放行	检验项目：全检
收验日期：2022.12.16	保质期：24 个月
报告日期：2022.12.28	检验依据：国家药品标准 YBZ-PFKL-2022002

质检章

检验结果：

检验项目	标准规定	检验数据	检验结论
[性状]	本品应为棕黄色至红棕色的颗粒；气微，味微苦。	本品为棕黄色的颗粒；气微，味微苦。	符合规定
[鉴别]	应检出百秋李醇	检出百秋李醇	符合规定
[检查]			
1. 水分	应不得过 8.0%	3.3%	符合规定
2. 溶化性	应符合规定	符合规定	符合规定
3. 粒度	不能通过一号筛和能通过五号筛的颗粒与粉末的总和应不大于 15%	2.1%	符合规定
4. 装量	应符合规定	符合规定	符合规定
[浸出物]	应不得少于 18.0%	21.4%	符合规定
[特征图谱]	应符合规定	符合规定	符合规定
[含量测定]	本品含挥发油应为 0.22%~0.64%（ml/g）。	0.47%（ml/g）	符合规定
	本品每 1g 含广藿香酮（$C_{12}H_{16}O_4$）应为 0.7mg~3.0mg。	1.65mg/g	
[微生物限度]			
需氧菌数	应≤10^3cfu/g	150cfu/g	符合规定
霉菌和酵母菌数	应≤10^2cfu/g	＜10cfu/g	符合规定
大肠埃希菌	每 1g 不得检出	每 1g 未检出	符合规定

检验结论：本品按 国家药品标准 YBZ-PFKL-2022002 标准检验,结果符合规定。

负责人：马湘云　　　　复核人：[illegible]

China Resources Sanjiu Modern Chinese Medicine Pharmaceutical Co., Ltd
Finished product inspection report

Sample number: BA221216008	Report Code: P20221228001B
Sample name: Pogostemon cablin formula granules	Batch number: 2212006C
Production unit: off-site workshop if CR Sanjiu Modern Chinese Medicine Pharmaceutical Co., Ltd (Huaibei)	Packaging specification: 250g/bottle
Packaging: oral solid medicine high-density polyethylene bottle	Quantity: 1777 bottles
Inspection purpose: release	Inspection items: full inspection
Receipt date: December 16, 2022	Shelf life: 24 months
Report date: December 28, 2022	Inspection basis: National Drug Standard YBZ-PFKL-2022002

Inspection results:

Inspection items	Standard Requirement	Inspection data	Item conclusion
[Appearance]	The product should be yellow-brown to red-brown granules, with slight odor, mildly bitter taste.	The product is yellow-brown to red-brown granules, with slight odor, mildly bitter taste.	Compliance
[Identification]	Patchouli alcohol should be detected.	Patchouli alcohol is detected.	Compliance
[Inspection]			
Water content	Not exceed 8.0%	3.3%	Compliance
Solubility	Should comply	Compliance	Compliance
Particle size	The sum of particles and powders that cannot pass through a No.1 sieve and those that can pass through a No.5 sieve should not exceed 15%.	2.1%	Compliance
Volume	Should comply	Compliance	Compliance
[Leachate]	Not less than 18.0%	21.4%	Compliance
[Characteristic chromatogram]	Should comply	Compliance	Compliance
[Content determination]	The product should contain volatile oil 0.22%~0.64%(ml/g).	0.47% (ml/g)	Compliance
	The product should contain patchouli alcohol ($C_{12}H_{16}O_4$) 0.7mg~3.0mg per 1g.	1.65mg/g	
[Microbial Limits]			
Total aerobic microbial count	Not exceed 10^3 cfu/g	150cfu/g	Compliance
Total mold and yeast count	Not exceed 10^2 cfu/g	<10cfu/g	Compliance
Escherichia coli	Should not be detected per 1g	Not be detected per 1g	Compliance

Conclusion: according to the national drug standard YBZ-PFKL-2022002, inspection results of the product are in compliance with the requirements.

Responsible person: Reviewer:

SOR-SMP-Z-QC-0005-01-002

华润三九现代中药制药有限公司

成品检验报告书

样品编号：BA220806021	报告书编号：P20220815024B
检品名称：黄连（黄连）配方颗粒	批号：　2208002C
生产单位：华润三九厂外车间（淮北）	包装规格：250g/瓶
包装：　口服固体药用高密度聚乙烯瓶	数量：　1970 瓶
检验目的：　成品放行	检验项目：　全检
收验日期：　2022.08.06	保质期：36 个月
报告日期：　2022.08.15	检验依据：国家药品标准 YBZ-PFKL-2021064

检验结果：

检验项目	标准规定	检验数据	检验结论
[性状]	本品应为黄棕色至深棕色的颗粒；气微，味极苦。	本品为黄棕色的颗粒；气微，味极苦。	符合规定
[鉴别]	应检出黄连（黄连）<br>应检出盐酸小檗碱	检出黄连（黄连）<br>检出盐酸小檗碱	符合规定
[检查]			
1. 水分	应不得过 8.0%	3.3%	符合规定
2. 溶化性	应符合规定	符合规定	符合规定
3. 粒度	不能通过一号筛和能通过五号筛的颗粒与粉末的总和应不大于 15%	2.1%	符合规定
4. 装量	应符合规定	符合规定	符合规定
[浸出物]	应不得少于 43.0%	64.6%	符合规定
[特征图谱]	应符合规定	符合规定	符合规定
[含量测定]	本品以盐酸小檗碱（$C_{20}H_{18}C_{1}NO_4$）计，每 1g 含小檗碱（$C_{20}H_{17}NO_4$）应为 110.0mg~210.0mg，	187.9mg/g	符合规定
	含表小檗碱（$C_{20}H_{17}NO_4$）、黄连碱（$C_{19}H_{13}NO_4$）和巴马汀（$C_{21}H_{21}NO_4$）的总量应为 78.0mg~160.0mg。	131.3mg/g	
[微生物限度]			
需氧菌数	应≤10^3cfu/g	100cfu/g	符合规定
霉菌和酵母菌数	应≤10^2cfu/g	＜100cfu/g	符合规定
大肠埃希菌	每 1g 不得检出	每 1g 未检出	符合规定

检验结论：本品按 国家药品标准 YBZ-PFKL-2021064 标准检验,结果符合规定。

负责人：刘伟　　　　复核人：张颖

China Resources Sanjiu Modern Chinese Medicine Pharmaceutical Co., Ltd
Finished product inspection report

Sample number: SA220620011	Report Code: P20220815024B
Sample name: Coptis Chinensis formula granules	Batch number: 2208002C
Production unit: off-site workshop of CR Sanjiu Co., Ltd (Huaibei)	Packaging specification: 250g/bottle
Packaging: oral solid medicine high-density polyethylene bottle	Quantity: 1970 bottles
Inspection purpose: release	Inspection items: full inspection
Receipt date: October 06, 2022	Shelf life: 36 months
Report date: October 15, 2022	Inspection basis: National Drug Standard YBZ-PFKL-2021064

Inspection results:

Inspection items	Standard Requirement	Inspection data	Item conclusion
[Appearance]	The product should be yellow-brown to dark brown granules, with slight odor, extremely bitter taste.	The product is yellow-brown to dark brown granules, with slight odor, extremely bitter taste.	Compliance
[Identification]	Coptis Chinensis should be detected	Coptis Chinensis is detected	Compliance
	Berberine hydrochloride should be detected	Berberine hydrochloride is detected	
[Inspection]			
Water content	Not exceed 8.0%	3.3%	Compliance
Solubility	Should comply	Compliance	Compliance
Particle size	The sum of particles and powders that cannot pass through a No.1 sieve and those that can pass through a No.5 sieve should not exceed 15%.	2.1%	Compliance
Volume	Should comply	Compliance	Compliance
[Leachate]	Not less than 43.0%	64.6%	Compliance
[Characteristic chromatogram]	Should comply	Compliance	Compliance
[Content determination]	Based on berberine hydrochloride ($C_{20}H_{18}C_1NO_4$), the product should contain the berberine ($C_{20}H_{17}NO_4$) 110.0mg~210.0mg per 1g;	187.9mg/g	Compliance
	Based on berberine hydrochloride ($C_{20}H_{18}C_1NO_4$), the product should contain epiberberine ($C_{20}H_{17}NO_4$), berberine ($C_{19}H_{13}NO_4$) and palmatine ($C_{21}H_{21}NO_4$) 78.0mg~160.0mg.	131. 3mg/g	
[Microbial Limits]			
Total aerobic microbial count	Not exceed 10^3 cfu/g	100cfu/g	Compliance
Total mold and yeast count	Not exceed 10^2 cfu/g	<100cfu/g	Compliance
Escherichia coli	Should not be detected per 1g	Not be detected per 1g	Compliance

Conclusion: according to the national drug standard YBZ-PFKL-2021064, inspection results of the product are in compliance with the requirements.

Responsible person: Reviewer:
