# Supplementary material for: Chinese herbal compound preparation Qing-Xin-Jie-Yu granules for intermediate coronary lesions in patients with stable coronary artery disease: Study protocol for a multicenter, randomized, double-blind, placebo-controlled trial
Source: PLoS One. 2024 Jul 16;19(7):e0307074. doi: 10.1371/journal.pone.0307074 (PMC11251585; doi:10.1371/journal.pone.0307074)
Supplement: S5 File — (PDF) [file pone.0307074.s008.pdf]

Chinese herbal compound preparation Qing-Xin-Jie-Yu granules for intermediate coronary lesions in patients with stable coronary artery disease: a randomized, double-blinded, placebo-controlled trial

Case Report Form

(Version Number: XYYY-V-1.0, Version Date: December 26, 2021)

**Subject Screening Number**	□□□
**Subject Number**	□□□
**Subject Name**	
**Subject Name Initial**	□□□□
**Subject Address**	
**Subject Phone number**	
**Test Centers Name**	□01 Xiyuan Hospital of China Academy of Chinese Medical Sciences
	□02 Guan'anmen Hospital of China Academy of Chinese Medical Sciences
	□03 Beijing Tongren Hospital Affiliated to Capital Medical University
**Investigator Name**	

Sponsor Unit: Xiyuan Hospital of China Academy of Chinese Medical Sciences
Leader Unit: Xiyuan Hospital of China Academy of Chinese Medical Sciences
Member Unit: Guan'anmen Hospital of China Academy of Chinese Medical Sciences; Beijing Tongren Hospital Affiliated to Capital Medical University

Instructions for filling out

1. Fill out the CRF in a black marker with clear handwriting.
2. Fill out the first page of the CRF correctly without any missing information.
3. Fill out the CRF completely and do not leave data items blank. Distinguish between unknown / not application / not available / not done. Data not known is indicated by “UK”. Data not applicable is indicated by “NA”. If results of the examination have not been obtained, it is not available and can be temporarily left out. If a piece of information or an experiment cannot be obtained or performed for some reason and cannot be remedied, it is not done and is indicated by “ND”.
4. For questions requiring a “0” (zero) answer, enter 0 instead of omitting it.
5. If fill in a mistake, do not use correction fluid or make arbitrary corrections, but draw a line through the error, write the correct data, and then indicate the initials of the modifier and the date of modification next to the data. For example, ~~23~~ 32, CJG 2004/09/27.
6. If an indicative choice such as Yes/No is encountered, please indicate with a “√” on the relevant option.
7. If a rating choice is encountered, please write down the number (e.g. 1 = mild, 2 = moderate, 3 = severe) and do not use a combination of choices (e.g. “1-2”, “1/2”). If necessary (e.g., to record adverse events), more than one number can be used.
8. Do not fill in the outside of the space, and do not confuse the data in different places by filling in improperly.
9. Do not use abbreviations that data processors may not know.
10. Comment only where specified in the CRF, and comments should be brief and concise.
11. Record the date in year/month/day/ format with 8 numbers. e.g., July 14, 2004 as 2004/07/14/. Record the time in a 24-hour format, e.g., 4:00 p.m. as 16:00, midnight as 00:00, and noon as 12:00.
12. Do your best to get the exact date. If the patient cannot remember a specific day or month, at least the year should be known. It is very important to record the dates of adverse events and medication dose changes.
13. Out-of-time visits: If, for special reasons, a subject is seen outside the visit time window or a test is performed outside the visit time window, please fill in the generated data on the out-of-time visits page, in the chronological order of data generation. If abnormal examination results require follow-up review, all review results are filled in the out-of-time visit page.

Measurement items and time window of data collection.

	Enrollment	Treatment			Close-out
	Baseline (Visit 1)	Month 2 (Visit 2)	Month 4 (Visit 3)	Month 6 (Visit 4)	Month 12 (Visit 5)
**ENROLLMENT**					
Eligibility screen	X				
Informed consent	X				
General information	X				
Concomitant medication	X	X	X	X	
Symptoms and signs	X	X	X	X	
Allocation	X				
**INTERVENTIONS**					
QXJYG + GDT	X	X	X	X	
Placebo + GDT	X	X	X	X	
**ASSESSMENTS**					
Efficacy outcomes					
CT-FFR	X			X	
CCTA [a]	X			X	
SAQ	X	X	X	X	
hs-CRP	X			X	
MMP-9	X			X	
Blood lipids [a]	X			X	
Carotid artery ultrasound [b]	X			X	
Safety outcomes					
Composite events of bleeding		X	X	X	
Laboratory test	X	X		X	
Electrocardiogram	X	X		X	
Adverse events		X	X	X	
Endpoints					
MACE		X	X	X	X
Medication adherence		X	X	X	

Note: [a] CCTA includes the percentage of diameter and area stenosis, coronary artery calcification score, and Gensini score derived from the CT images. [b] Blood lipids include TC, TG, LDL-C, HDL-C, Apo A1, Apo B1, Lp(a). [c] Carotid ultrasound parameters include IMT (mm), carotid plaque length × thickness (mm) and carotid lumen stenosis (%). [d] Laboratory tests include complete blood count, coagulation function, liver and renal function, blood glucose, urine and stool routine.

Study No. A00908	Center No. |_|_|	Name Initial |_|_|_|_|	Subject No. |_|_|_|	Visit Date |_|_|_|_|year|_|_|month|_|_|day	Visit |1|	Screening Period

Informed Consent Form (ICF)

Note: Subjects must sign an ICF prior to the start of all screening tests!

Is the informed consent signed by the subject?	☐$_0$ No ☐ ☐$_1$ Yes
Signing Date	|_|_|_|_|year|_|_|month|_|_|day

Demographic Information

**Birth date**	|_|_|_|_|year|_|_|month|_|_|day	Gender	☐$_1$ Male ☐$_2$ Female
**Ethnicity**	☐$_1$ Han Chinese	☐$_2$ Others → (please specify:____________)	

Basic Characteristics

**Marital status**	☐$_1$ Unmarried ☐$_2$ Married ☐$_4$ Divorced ☐$_5$ Widowed ☐$_{66}$ Unknown
**Education level**	☐$_1$ Illiterate ☐$_2$ Elementary school ☐$_3$ Junior high school ☐$_4$ High school/junior college ☐$_5$ College ☐$_{66}$ Unknown ☐$_7$ University and above
**Occupation**	☐$_1$ mental labor ☐$_2$ physical labor ☐$_{88}$ Others→(please specify:_________)

Past Medical History

Does the subject have a history of other diseases?

☐$_0$ No ☐$_1$ Yes→Please fill in the table below.

No	Disease	Start date*	End date*	Continue or not
1		|_|_|_|_|year|_|_|month|_|_|day	|_|_|_|_|year|_|_|month|_|_|day	☐$_1$ Yes ☐$_0$ No
2		|_|_|_|_|year|_|_|month|_|_|day	|_|_|_|_|year|_|_|month|_|_|day	☐$_1$ Yes ☐$_0$ No
3		|_|_|_|_|year|_|_|month|_|_|day	|_|_|_|_|year|_|_|month|_|_|day	☐$_1$ Yes ☐$_0$ No
4		|_|_|_|_|year|_|_|month|_|_|day	|_|_|_|_|year|_|_|month|_|_|day	☐$_1$ Yes ☐$_0$ No
5		|_|_|_|_|year|_|_|month|_|_|day	|_|_|_|_|year|_|_|month|_|_|day	☐$_1$ Yes ☐$_0$ No
6		|_|_|_|_|year|_|_|month|_|_|day	|_|_|_|_|year|_|_|month|_|_|day	☐$_1$ Yes ☐$_0$ No
7		|_|_|_|_|year|_|_|month|_|_|day	|_|_|_|_|year|_|_|month|_|_|day	☐$_1$ Yes ☐$_0$ No
8		|_|_|_|_|year|_|_|month|_|_|day	|_|_|_|_|year|_|_|month|_|_|day	☐$_1$ Yes ☐$_0$ No

*** Note: Please fill in start date and end date as completely as possible. if unclear, “UK” can be filled in.**

Study No.	Center No.	Name Initial	Subject No.	Visit Date	Visit	Screening
A00908	|_|_|	|_|_|_|_|	|_|_|_|	|_|_|_|_|year|_|_|month|_|_|day	|1|	Period

Pre-study Medication

Any medication or non-medication used in the three months prior to the current visit? ☐0 No ☐1 Yes → Please fill in the table below.

No	Medication or non-medication name①	Single dose	Dose unit②	Fre-quency③	Administra-tion route④	Indication of use	Start date⑤	End date⑥ or still in use
1							|_|_|_|_|year|_|_|month|_|_|day	|_|_|_|_|year|_|_|month|_|_|day or go on ☐1
2							|_|_|_|_|year|_|_|month|_|_|day	|_|_|_|_|year|_|_|month|_|_|day or go on ☐1
3							|_|_|_|_|year|_|_|month|_|_|day	|_|_|_|_|year|_|_|month|_|_|day or go on ☐1
4							|_|_|_|_|year|_|_|month|_|_|day	|_|_|_|_|year|_|_|month|_|_|day or go on ☐1
5							|_|_|_|_|year|_|_|month|_|_|day	|_|_|_|_|year|_|_|month|_|_|day or go on ☐1
6							|_|_|_|_|year|_|_|month|_|_|day	|_|_|_|_|year|_|_|month|_|_|day or go on ☐1
7							|_|_|_|_|year|_|_|month|_|_|day	|_|_|_|_|year|_|_|month|_|_|day or go on ☐1
8							|_|_|_|_|year|_|_|month|_|_|day	|_|_|_|_|year|_|_|month|_|_|day or go on ☐1
9							|_|_|_|_|year|_|_|month|_|_|day	|_|_|_|_|year|_|_|month|_|_|day or go on ☐1
10							|_|_|_|_|year|_|_|month|_|_|day	|_|_|_|_|year|_|_|month|_|_|day or go on ☐1

Study No.<br>A00908	Center No. |_|_|	Name Initial |_|_|_|_|	Subject No. |_|_|_|	Visit Date |_|_|_|_|year|_|_|month|_|_|day	Visit |1|	Screening<br>Period

11							|_|_|_|_|year|_|_|month|_|_|day	|_|_|_|_|year|_|_|month|_|_|day or go on □1
12							|_|_|_|_|year|_|_|month|_|_|day	|_|_|_|_|year|_|_|month|_|_|day or go on □1
13							|_|_|_|_|year|_|_|month|_|_|day	|_|_|_|_|year|_|_|month|_|_|day or go on □1
14							|_|_|_|_|year|_|_|month|_|_|day	|_|_|_|_|year|_|_|month|_|_|day or go on □1
15							|_|_|_|_|year|_|_|month|_|_|day	|_|_|_|_|year|_|_|month|_|_|day or go on □1

Remarks: ① If non-medication, fill in NA in the column of Single Dose, Dose Unit and Administration Route.

② Dose unit: 1.mg, 2.ug, 3.mL, 4.g, 5.IU, 6.TABLET, 7.CAPSULE, 8.PUFF, 9.Others, please specify, 10.NA

③ Frequency: 1.BID (twice a day), 2.TID (three times a day), 3.QID (four times a day), 4.QOD (every other day), 5.QM(once a month), 6.PRN(on demand), 7.UNKONWN(unknown), 8.QD(once a day), 9.Others, please specify

④ Administration route: 1.oral, 2.topical, 3.subcutaneous, 4. transdermal, 5.intraocular, 6.intramuscular, 7.respiratory/inhalation, 8.intralesional, 9.intraperitoneal, 10. nasal, 11. vaginal, 12. rectal, 13. sublingual, 14. intravenous injection, 15.Others, please specify, 16.NA

⑤ Please fill in start date and end date as completely as possible. if unclear, "UK" can be filled in.

.

<table>
<tr><td>Study No.
A00908</td><td>Center No.
|_|_|</td><td>Name Initial
|_|_|_|_|</td><td>Subject No.
|_|_|_|</td><td>Visit Date
|_|_|_|_|year|_|_|month|_|_|day</td><td>Visit
|1|</td><td>Screening
Period</td></tr>
</table>

Smoking History

<table>
<tr><td>Smoking status</td><td>☐$_{1}$ Never used
☐$_{2}$ Use now
☐$_{3}$ Used in past</td><td>Number of cigarettes smoked</td><td>☐$_{1}$ Less than 3 cigarettes/day
☐$_{2}$ 3-10 cigarettes/day
☐$_{3}$ More than 10 cigarettes/day</td></tr>
<tr><td>Start date</td><td>|_|_|_|_|year|_|_|month|_|_|day</td><td>End date</td><td>|_|_|_|_|year|_|_|month|_|_|day</td></tr>
</table>

Drinking History

<table>
<tr><td>Drinking status</td><td>☐$_{1}$ Never used
☐$_{2}$ Use now
☐$_{3}$ Used in past</td><td>Alcohol consumption</td><td>☐$_{1}$ Less than 50 grams/day
☐$_{2}$ 50-100 grams/day
☐$_{3}$ more than 100 grams/day</td></tr>
<tr><td>Start date</td><td>|_|_|_|_|year|_|_|month|_|_|day</td><td>End date</td><td>|_|_|_|_|year|_|_|month|_|_|day</td></tr>
</table>

Drug Allergy History

<table>
<tr><td>Any history of drug allergy?
☐$_{0}$ No ☐$_{1}$ Yes, please record</td><td>Allergic drugs:
____
____
________________________________</td></tr>
</table>

Vital signs

<table>
<tr><th>Measurement items</th><th>Value</th><th>ND</th><th>Measurement location</th></tr>
<tr><td>Height (cm)</td><td>|_|_|_|. |_|</td><td>☐$_{77}$</td><td>☐$_{99}$ NA</td></tr>
<tr><td>Weight (Kg)</td><td>|_|_|_|. |_|</td><td>☐$_{77}$</td><td>☐$_{99}$ NA</td></tr>
<tr><td>BMI(Kg/m^{2})</td><td>|_|_|. |_|</td><td>☐$_{77}$</td><td>☐$_{99}$ NA</td></tr>
<tr><td>Breathing (times/min)</td><td>|_|_|</td><td>☐$_{77}$</td><td>☐$_{99}$ NA</td></tr>
<tr><td>HR (beats/min)</td><td>|_|_|_|</td><td>☐$_{77}$</td><td>☐$_{1}$ Brachial artery ☐$_{2}$ Carotid artery ☐$_{3}$ Radial artery</td></tr>
<tr><td>Temperature (℃)</td><td>|_|_|. |_|</td><td>☐$_{77}$</td><td>☐$_{4}$ Axillary ☐$_{5}$ Ear</td></tr>
<tr><td>SBP (mmHg)</td><td>|_|_|_|</td><td>☐$_{77}$</td><td>☐$_{1}$ Brachial artery ☐$_{2}$ Ankle</td></tr>
<tr><td>DBP (mmHg)</td><td>|_|_|_|</td><td>☐$_{77}$</td><td>☐$_{1}$ Brachial artery ☐$_{2}$ Ankle</td></tr>
</table>

Note: ND: Not done; HR: Resting heart rate; SBP: Systolic blood pressure; DBP: Diastolic blood pressure.

Study No. A00908	Center No. |_|_|	Name Initial |_|_|_|_|	Subject No. |_|_|_|	Visit Date |_|_|_|_|year|_|_|month|_|_|day	Visit |1|	Screening Period

Physical examination

**Items**	**Examination results**	**Abnormal (please specify)**
Heart examination	☐$_1$ Normal<br>☐$_2$ Abnormal<br>☐$_{77}$ Not done	
Others	☐$_1$ Normal<br>☐$_2$ Abnormal<br>☐$_{77}$ Not done	

Current medical history of CAD

Diagnosis:<br>CAD ☐$_1$ Yes ☐$_0$ No	Date of first diagnosis: |_|_|_|_|year|_|_|month|_|_|day
	☐$_1$ SCAD ☐$_{88}$ Others, please specify:____________
	CCS grading: ☐$_1$ grade I ☐$_2$ grade II ☐$_3$ grade III ☐$_4$ grade IV

Note: CAD: Coronary artery disease; SCAD: Stable coronary artery disease

<table>
<tr><td>Study No.
A00908</td><td>Center No.
|_|_|</td><td>Name Initial
|_|_|_|_|</td><td>Subject No.
|_|_|_|</td><td>Visit Date
|_|_|_|_|year|_|_|month|_|_|day</td><td>Visit
|1|</td><td>Screening
Period</td></tr>
</table>

Seattle Angina Questionnaire

Q1: Over the past 4 weeks, the following levels have been limited due to chest pain, chest tightness, and angina

	Severely limited	Moderately limited	Mildly limited	Slightly limited	Not limited	Limited by Other reasons
Dress yourself	☐$_1$	☐$_2$	☐$_3$	☐$_4$	☐$_5$	☐$_6$
Walk indoors	☐$_1$	☐$_2$	☐$_3$	☐$_4$	☐$_5$	☐$_6$
Shower	☐$_1$	☐$_2$	☐$_3$	☐$_4$	☐$_5$	☐$_6$
Climb or stairs (going up three floors without stopping)	☐$_1$	☐$_2$	☐$_3$	☐$_4$	☐$_5$	☐$_6$
Outdoor activities or debris picking	☐$_1$	☐$_2$	☐$_3$	☐$_4$	☐$_5$	☐$_6$
Easy walk (1 km)	☐$_1$	☐$_2$	☐$_3$	☐$_4$	☐$_5$	☐$_6$
Jogging (1 km)	☐$_1$	☐$_2$	☐$_3$	☐$_4$	☐$_5$	☐$_6$
Lift or move heavy objects	☐$_1$	☐$_2$	☐$_3$	☐$_4$	☐$_5$	☐$_6$
Strenuous exercise (such as swimming or playing ball)	☐$_1$	☐$_2$	☐$_3$	☐$_4$	☐$_5$	☐$_6$

Q2: Compared to 4 weeks ago, episodes of chest pain, chest tightness and angina when maximum intensity activity was performed.

☐$_1$ Significant increase ☐$_2$ Slight increase ☐$_3$ Same ☐$_4$ Slight decrease ☐$_5$ Significant decrease

Q3: Over the past 4 weeks, average numbers of episodes of chest pain, chest tightness, and angina.

☐$_1$ ≥4times/day ☐$_2$ 1-3 times/day ☐$_3$ ≥3times/week ☐$_4$ 1-2times/week ☐$_5$ <1times/week ☐$_6$ No episode

Q4: Over the past 4 weeks, average numbers of times nitro drugs (such as nitroglycerin) were taken for chest pain, chest tightness, and angina.

☐$_1$ ≥4times/day ☐$_2$ 1-3 times/day ☐$_3$ ≥3times/week ☐$_4$ 1-2times/week ☐$_5$ <1times/week ☐$_6$ No use

Q5: Worries caused by chest pain, chest tightness, and angina that require medication as prescribed.

☐$_1$ Severe ☐$_2$ Moderate ☐$_3$ Mild ☐$_4$ Rare ☐$_5$ None ☐$_6$ Physician not administered

Q6: Satisfaction degree with various measures for the treatment of chest pain, chest tightness and angina.

☐$_1$ Dissatisfied ☐$_2$ Mostly dissatisfied ☐$_3$ Partially satisfied ☐$_4$ Mostly satisfied ☐$_5$ Highly satisfied

Q7: Satisfaction degree with the doctor's explanations for chest pain, chest tightness, and angina.

☐$_1$ Dissatisfied ☐$_2$ Mostly dissatisfied ☐$_3$ Partially satisfied ☐$_4$ Mostly satisfied ☐$_5$ Highly satisfied

Q8: Overall satisfaction degree with current treatment of chest pain, chest tightness, and angina.
☐ $_1$ Dissatisfied ☐ $_2$ Mostly dissatisfied ☐ $_3$ Partially satisfied ☐ $_4$ Mostly satisfied ☐ $_5$ Highly satisfied

Q9: Over the past 4 weeks, the extent to which chest pain, chest tightness and angina affected the joy of life.
☐ $_1$ Dissatisfied ☐ $_2$ Mostly dissatisfied ☐ $_3$ Partially satisfied ☐ $_4$ Mostly satisfied ☐ $_5$ Highly satisfied

Q10: How would you feel if you still had chest pain, chest tightness and angina in your future life?☐ $_1$ Dissatisfied ☐ $_2$ Mostly dissatisfied ☐ $_3$ Partially satisfied ☐ $_4$ Mostly satisfied ☐ $_5$ Highly satisfied

Q11: The degree of worry about heart attacks and sudden deaths.
☐ $_1$ Worried all the time ☐ $_2$ Worried often ☐ $_3$ Worried sometimes ☐ $_4$ Rarely worried ☐ $_5$ Never worried

Note: Standard Scores = (actual score - minimum score in this domain) / (highest score in this domain - minimum score in this domain)

- **Score of physical activity limitation (Q 1):**
- **Score of angina steady-state (Q 2):**
- **Score of angina attacks (Q3-Q4):**
- **Score of treatment satisfaction (Q5-Q8):**
- **Score of disease awareness (Q9-Q11):**

Coronary CTA (Inspection date: |_|_|_|_|year|_|_|month|_|_|day)

Lesioned vessels		FFR (CT)	DS (%)	AS (%)	Gensini Score	CACS	Image quality*
LM							☐1 ☐2 ☐3
LAD	prox						☐1 ☐2 ☐3
LAD	mid						☐1 ☐2 ☐3
LAD	apic						☐1 ☐2 ☐3
	1st D						☐1 ☐2 ☐3
	2nd D						☐1 ☐2 ☐3
LCX	prox						☐1 ☐2 ☐3
LCX	apic						☐1 ☐2 ☐3
	OM						☐1 ☐2 ☐3
	PD						☐1 ☐2 ☐3
	PL						☐1 ☐2 ☐3
RCA	prox						☐1 ☐2 ☐3
RCA	mid						☐1 ☐2 ☐3
RCA	dist						☐1 ☐2 ☐3
	PD						☐1 ☐2 ☐3
Totaol Score							☐1 ☐2 ☐3
Description of coronary CTA results							

Note: FFR(CT): Coronary computed tomography angiography-derived fractional flow reserve; DS(%): Percentage of diameter stenosis; % AS: Percentage of area stenosis; CACS: Coronary artery calcification score; *Image quality: ☐1 Excellent, ☐2 Fair, ☐3 Poor.

Observing physicians__________________ Date _ _ _ _ year _ _ month _ _ day

Carotid Artery Ultrasound

Whether to test	☐1 Yes ☐0 No	
Testing date	|_|_|_|_|year|_|_|month|_|_|day	
	Left carotid artery	Right carotid artery
Carotid intima-media thickness (IMT) (mm)		
Is plaque detected	☐1Yes ☐0 No	☐1Yes ☐0 No
Carotid plaque length × thickness (longitudinal section) (mm)		
Plaque type	☐1Vulnerable ☐0Non-vulnerable	☐1Vulnerable ☐0Non-vulnerable
Degree of carotid lumen stenosis (%)		
Description of carotid ultrasound results		

Blood lipids **(Sample date: |_|_|_|_|year|_|_|month|_|_|day)**

Indicators	Test value	Standard unit	Clinical significance determination	
TC		mmol/L	☐1 ☐2 ☐3 ☐77	
TG		mmol/L	☐1 ☐2 ☐3 ☐77	
HDL-C		mmol/L	☐1 ☐2 ☐3 ☐77	
LDL-C		mmol/L	☐1 ☐2 ☐3 ☐77	
Apo A1		g/L	☐1 ☐2 ☐3 ☐77	
Apo B		g/L	☐1 ☐2 ☐3 ☐77	
Lp(a)		nmol/L	☐1 ☐2 ☐3 ☐77	

Note: ☐1 Normal; ☐2 Abnormal without clinical significance; ☐3 Abnormal with clinical significance; ☐77 Not done.

Inflammatory factors **(Sample date: |_|_|_|_|year|_|_|month|_|_|day)**

Indicators	Test value	Standard unit	Clinical significance determination	
hs-CRP		mg/L	☐1 ☐2 ☐3 ☐77	
MMP-9		ng/mL	☐1 ☐2 ☐3 ☐77	

Note: ☐1 Normal; ☐2 Abnormal without clinical significance; ☐3 Abnormal with clinical significance; ☐77 Not done.

Observing physicians__________________ Date _ _ _ _ year _ _ month _ _ day

Laboratory tests

<table>
<tr><th>Indicators</th><th>Test value</th><th>Standard unit</th><th colspan="2">Clinical significance determination</th></tr>
<tr><td>Blood routine</td><td colspan="3">Sample date: |_|_|_|_|year|_|_|month|_|_|day</td><td>Abnormalities</td></tr>
<tr><td>RBC</td><td></td><td>×10^{12}/L</td><td>☐$_{1}$ ☐$_{2}$ ☐$_{3}$ ☐$_{77}$</td><td></td></tr>
<tr><td>HBG</td><td></td><td>g/L</td><td>☐$_{1}$ ☐$_{2}$ ☐$_{3}$ ☐$_{77}$</td><td></td></tr>
<tr><td>WBC</td><td></td><td>×10^{9}/L</td><td>☐$_{1}$ ☐$_{2}$ ☐$_{3}$ ☐$_{77}$</td><td></td></tr>
<tr><td>NEUT%</td><td></td><td>%</td><td>☐$_{1}$ ☐$_{2}$ ☐$_{3}$ ☐$_{77}$</td><td></td></tr>
<tr><td>PLT</td><td></td><td>×10^{9}/L</td><td>☐$_{1}$ ☐$_{2}$ ☐$_{3}$ ☐$_{77}$</td><td></td></tr>
<tr><td>Coagulation</td><td colspan="3">Sample date: |_|_|_|_|year|_|_|month|_|_|day</td><td>Abnormalities</td></tr>
<tr><td>PT</td><td></td><td></td><td>☐$_{1}$ ☐$_{2}$ ☐$_{3}$ ☐$_{77}$</td><td></td></tr>
<tr><td>TT</td><td></td><td></td><td>☐$_{1}$ ☐$_{2}$ ☐$_{3}$ ☐$_{77}$</td><td></td></tr>
<tr><td>INR</td><td></td><td></td><td>☐$_{1}$ ☐$_{2}$ ☐$_{3}$ ☐$_{77}$</td><td></td></tr>
<tr><td>APTT</td><td></td><td></td><td>☐$_{1}$ ☐$_{2}$ ☐$_{3}$ ☐$_{77}$</td><td></td></tr>
<tr><td>fibrinogen</td><td></td><td></td><td>☐$_{1}$ ☐$_{2}$ ☐$_{3}$ ☐$_{77}$</td><td></td></tr>
<tr><td>Urine routine</td><td colspan="3">Sample date: |_|_|_|_|year|_|_|month|_|_|day</td><td>Abnormalities</td></tr>
<tr><td>PRO</td><td></td><td>Qualitative</td><td>☐$_{1}$ ☐$_{2}$ ☐$_{3}$ ☐$_{77}$</td><td></td></tr>
<tr><td>GLU</td><td></td><td>Qualitative</td><td>☐$_{1}$ ☐$_{2}$ ☐$_{3}$ ☐$_{77}$</td><td></td></tr>
<tr><td>RBC</td><td></td><td>pc/μl</td><td>☐$_{1}$ ☐$_{2}$ ☐$_{3}$ ☐$_{77}$</td><td></td></tr>
<tr><td>WBC</td><td></td><td>pc/μl</td><td>☐$_{1}$ ☐$_{2}$ ☐$_{3}$ ☐$_{77}$</td><td></td></tr>
<tr><td>HCG</td><td colspan="4">☐$_{0}$ Negative ☐$_{2}$ Positive ☐$_{3}$ Not application ☐$_{77}$ Not done, reason______</td></tr>
<tr><td>Stool routine</td><td colspan="3">Sample date: |_|_|_|_|year|_|_|month|_|_|day</td><td>Abnormalities</td></tr>
<tr><td>WBC</td><td></td><td>Qualitative</td><td>☐$_{1}$ ☐$_{2}$ ☐$_{3}$ ☐$_{77}$</td><td></td></tr>
<tr><td>OB</td><td></td><td>Qualitative</td><td>☐$_{1}$ ☐$_{2}$ ☐$_{3}$ ☐$_{77}$</td><td></td></tr>
<tr><td>Biochemical test</td><td colspan="3">Sample date: |_|_|_|_|year|_|_|month|_|_|day</td><td>Abnormalities</td></tr>
<tr><td>ALT</td><td></td><td>U/L</td><td>☐$_{1}$ ☐$_{2}$ ☐$_{3}$ ☐$_{77}$</td><td></td></tr>
<tr><td>AST</td><td></td><td>U/L</td><td>☐$_{1}$ ☐$_{2}$ ☐$_{3}$ ☐$_{77}$</td><td></td></tr>
<tr><td>BUN</td><td></td><td>mmol/L</td><td>☐$_{1}$ ☐$_{2}$ ☐$_{3}$ ☐$_{77}$</td><td></td></tr>
<tr><td>Scr</td><td></td><td>μmol/L</td><td>☐$_{1}$ ☐$_{2}$ ☐$_{3}$ ☐$_{77}$</td><td></td></tr>
<tr><td>UA</td><td></td><td>μmol/L</td><td>☐$_{1}$ ☐$_{2}$ ☐$_{3}$ ☐$_{77}$</td><td></td></tr>
<tr><td>Blood glucose</td><td colspan="3">Sample date: |_|_|_|_|year|_|_|month|_|_|day</td><td>Abnormalities</td></tr>
<tr><td>FBG</td><td></td><td>mmol/L</td><td>☐$_{1}$ ☐$_{2}$ ☐$_{3}$ ☐$_{77}$</td><td></td></tr>
</table>

Note: ☐$_{1}$ Normal; ☐$_{2}$ Abnormal without clinical significance; ☐$_{3}$ Abnormal with clinical significance; ☐$_{77}$ Not done.

Observing physicians__________________ Date _ _ _ _ year _ _ month _ _ day

Electrocardiogram (ECG)

Whether to test	☐$_1$ Yes ☐$_0$ No
Testing date	|_|_|_|_|year|_|_|month|_|_|day
Testing position	☐$_1$ Sitting ☐$_2$ Standing ☐$_3$ Supine ☐$_{88}$ Others.
Description of ECG results	
Determination of ECG clinical significance	☐$_1$ Normal ☐$_2$ Abnormal without clinical significance<br>☐$_3$ Abnormal with clinical significance
	Description of abnormalities:

Observing physicians__________________ Date _ _ _ _ year _ _ month _ _ day

Study No.	Center No.	Name Initial	Subject No.	Visit Date	Visit	Screening
A00908	|_|_|	|_|_|_|_|	|_|_|_|	|_|_|_|_|year|_|_|month|_|_|day	|1|	Period

Sticky Place of Inspection Report Forms for Screening Period

Study No. A00908	Center No. |_|_|	Name Initial |_|_|_|_|	Subject No. |_|_|_|	Visit Date |_|_|_|_|year|_|_|month|_|_|day	Visit |1|	Screening Period

Inclusion criteria

**Inclusion criteria** (If "no" to any of the following, you cannot be in the trial)		Yes	No	NA
1	Meeting diagnostic criteria for CAD and CCTA indicating at least one major coronary lumen diameter stenosis between 50% and 70%	□1	□0	□99
2	Conforming to the TCM syndrome differentiation criteria of blood-stasis.	□1	□0	□99
3	Heart function grading of NYHA grade Ⅰ-Ⅱ.	□1	□0	□99
4	ages 18-80 years.	□1	□0	□99
5	Subjects who are informed and voluntarily sign the ICF.	□1	□0	□99

Exclusion criteria

**Exclusion criteria** (If "yes" to any of the following, you cannot be in the trial)		Yes	No	NA
1	Having previous coronary stent implantation, CABG or MI within 3 months.	□1	□0	□99
2	With diffuse lesions throughout the diseased vessels.	□1	□0	□99
3	Having previous other cardiac operations, such as valve replacement.	□1	□0	□99
4	With severe heart, liver and renal insufficiency, thereby not suitable for CAG and related examination and treatment.	□1	□0	□99
5	Suffering from mental disorders.	□1	□0	□99
6	Suffering from hepatitis, tuberculosis, AIDS and other infectious diseases.	□1	□0	□99
7	Allergic constitution.	□1	□0	□99
8	Pregnant and lactating women.	□1	□0	□99
9	Life expectancy is less than one year.	□1	□0	□99
10	Participating in other clinical trials within the last 3 months.	□1	□0	□99

Study No. A00908	Center No. |_|_|	Name Initial |_|_|_|_|	Subject No. |_|_|_|	Visit Date |_|_|_|_|year|_|_|month|_|_|day	Visit |1|	Screening Period

Screening results

Screening qualified for entry into this trial. ☐0 No ☐1 Yes

We have reviewed all the screening records of the subject and confirmed that sufficient clinical information has been collected. The subject meet all the inclusion criteria and none of the exclusion criteria, and agree to be enrolled in this clinical trial.

Drug distribution

Date of distribution	Number of distribution
|_|_|_|_|year|_|_|month|_|_|day	

Appointment for next follow-up visit: |_|_|_|_|year|_|_|month|_|_|day

**I have verified the completeness and accuracy of the information filled out for this visit.**<br>**Signature of the observing physician__________ Date: |_|_|_|_|year|_|_|month|_|_|day**

Study No. A00908	Center No. |_|_|	Name Initial |_|_|_|_|	Subject No. |_|_|_|	Visit Date |_|_|_|_|year|_|_|month|_|_|day	Visit |2|	Treatment Period

Vital signs

Measurement items	Value	ND	Measurement location
Height (cm)	|_|_|_|. |_|	☐77	☐99 NA
Weight (Kg)	|_|_|_|. |_|	☐77	☐99 NA
BMI(Kg/m^2)	|_|_|. |_|	☐77	☐99 NA
Breathing (times/min)	|_|_|	☐77	☐99 NA
HR (beats/min)	|_|_|_|	☐77	☐1 Brachial artery ☐2 Carotid artery ☐3 Radial artery
Temperature (℃)	|_|_|. |_|	☐77	☐4 Axillary ☐5 Ear
SBP (mmHg)	|_|_|_|	☐77	☐1 Brachial artery ☐2 Ankle
DBP (mmHg)	|_|_|_|	☐77	☐1 Brachial artery ☐2 Ankle

Note: ND: Not done; HR: Resting heart rate; SBP: Systolic blood pressure; DBP: Diastolic blood pressure.

Physical examination

Items	Examination results	Abnormal (please specify)
Heart examination	☐1 Normal<br>☐2 Abnormal<br>☐77 Not done	
Others	☐1 Normal<br>☐2 Abnormal<br>☐77 Not done	

Study No.	Center No.	Name Initial	Subject No.	Visit Date	Visit	Treatment
A00908	|_|_|	|_|_|_|_|	|_|_|_|	|_|_|_|_|year|_|_|month|_|_|day	|2|	Period

Seattle Angina Questionnaire

Q1: Over the past 4 weeks, the following levels have been limited due to chest pain, chest tightness, and angina

	Severely limited	Moderately limited	Mildly limited	Slightly limited	Not limited	Limited by Other reasons
Dress yourself	☐$_1$	☐$_2$	☐$_3$	☐$_4$	☐$_5$	☐$_6$
Walk indoors	☐$_1$	☐$_2$	☐$_3$	☐$_4$	☐$_5$	☐$_6$
Shower	☐$_1$	☐$_2$	☐$_3$	☐$_4$	☐$_5$	☐$_6$
Climb or stairs (going up three floors without stopping)	☐$_1$	☐$_2$	☐$_3$	☐$_4$	☐$_5$	☐$_6$
Outdoor activities or debris picking	☐$_1$	☐$_2$	☐$_3$	☐$_4$	☐$_5$	☐$_6$
Easy walk (1 km)	☐$_1$	☐$_2$	☐$_3$	☐$_4$	☐$_5$	☐$_6$
Jogging (1 km)	☐$_1$	☐$_2$	☐$_3$	☐$_4$	☐$_5$	☐$_6$
Lift or move heavy objects	☐$_1$	☐$_2$	☐$_3$	☐$_4$	☐$_5$	☐$_6$
Strenuous exercise (such as swimming or playing ball)	☐$_1$	☐$_2$	☐$_3$	☐$_4$	☐$_5$	☐$_6$

Q2: Compared to 4 weeks ago, episodes of chest pain, chest tightness and angina when maximum intensity activity was performed.

☐$_1$ Significant increase ☐$_2$ Slight increase ☐$_3$ Same ☐$_4$ Slight decrease ☐$_5$ Significant decrease

Q3: Over the past 4 weeks, average numbers of episodes of chest pain, chest tightness, and angina.

☐$_1$ ≥4times/day ☐$_2$ 1-3 times/day ☐$_3$ ≥3times/week ☐$_4$ 1-2times/week ☐$_5$ <1times/week ☐$_6$ No episode

Q4: Over the past 4 weeks, average numbers of times nitro drugs (such as nitroglycerin) were taken for chest pain, chest tightness, and angina.

☐$_1$ ≥4times/day ☐$_2$ 1-3 times/day ☐$_3$ ≥3times/week ☐$_4$ 1-2times/week ☐$_5$ <1times/week ☐$_6$ No use

Q5: Worries caused by chest pain, chest tightness, and angina that require medication as prescribed.

☐$_1$ Severe ☐$_2$ Moderate ☐$_3$ Mild ☐$_4$ Rare ☐$_5$ None ☐$_6$ Physician not administered

Q6: Satisfaction degree with various measures for the treatment of chest pain, chest tightness and angina.

☐$_1$ Dissatisfied ☐$_2$ Mostly dissatisfied ☐$_3$ Partially satisfied ☐$_4$ Mostly satisfied ☐$_5$ Highly satisfied

Q7: Satisfaction degree with the doctor's explanations for chest pain, chest tightness, and angina.

☐$_1$ Dissatisfied ☐$_2$ Mostly dissatisfied ☐$_3$ Partially satisfied ☐$_4$ Mostly satisfied ☐$_5$ Highly satisfied

Q8: Overall satisfaction degree with current treatment of chest pain, chest tightness, and angina.
☐1 Dissatisfied ☐2 Mostly dissatisfied ☐3 Partially satisfied ☐4 Mostly satisfied ☐5 Highly satisfied

Q9: Over the past 4 weeks, the extent to which chest pain, chest tightness and angina affected the joy of life.
☐1 Dissatisfied ☐2 Mostly dissatisfied ☐3 Partially satisfied ☐4 Mostly satisfied ☐5 Highly satisfied

Q10: How would you feel if you still had chest pain, chest tightness and angina in your future life?☐1 Dissatisfied ☐2 Mostly dissatisfied ☐3 Partially satisfied ☐4 Mostly satisfied ☐5 Highly satisfied

Q11: The degree of worry about heart attacks and sudden deaths.
☐1 Worried all the time ☐2 Worried often ☐3 Worried sometimes ☐4 Rarely worried ☐5 Never worried

Note: Standard Scores = (actual score - minimum score in this domain) / (highest score in this domain - minimum score in this domain)

- **Score of physical activity limitation (Q 1):**
- **Score of angina steady-state (Q 2):**
- **Score of angina attacks (Q3-Q4):**
- **Score of treatment satisfaction (Q5-Q8):**
- **Score of disease awareness (Q9-Q11):**

Study No.	Center No.	Name Initial	Subject No.	Visit Date	Visit	Screening
A00908	|_|_|	|_|_|_|_|	|_|_|_|	|_|_|_|_|year|_|_|month|_|_|day	|2|	Period

Laboratory tests

**Indicators**	**Test value**	**Standard unit**	**Clinical significance determination**	
**Blood routine**	**Sample date: |_|_|_|_|year|_|_|month|_|_|day**			**Abnormalities**
RBC		×10^{12}/L	☐$_1$ ☐$_2$ ☐$_3$ ☐$_{77}$	
HBG		g/L	☐$_1$ ☐$_2$ ☐$_3$ ☐$_{77}$	
WBC		×10^{9}/L	☐$_1$ ☐$_2$ ☐$_3$ ☐$_{77}$	
NEUT%		%	☐$_1$ ☐$_2$ ☐$_3$ ☐$_{77}$	
PLT		×10^{9}/L	☐$_1$ ☐$_2$ ☐$_3$ ☐$_{77}$	
**Coagulation**	**Sample date: |_|_|_|_|year|_|_|month|_|_|day**			**Abnormalities**
PT			☐$_1$ ☐$_2$ ☐$_3$ ☐$_{77}$	
TT			☐$_1$ ☐$_2$ ☐$_3$ ☐$_{77}$	
INR			☐$_1$ ☐$_2$ ☐$_3$ ☐$_{77}$	
APTT			☐$_1$ ☐$_2$ ☐$_3$ ☐$_{77}$	
fibrinogen			☐$_1$ ☐$_2$ ☐$_3$ ☐$_{77}$	
**Urine routine**	**Sample date: |_|_|_|_|year|_|_|month|_|_|day**			**Abnormalities**
PRO		Qualitative	☐$_1$ ☐$_2$ ☐$_3$ ☐$_{77}$	
GLU		Qualitative	☐$_1$ ☐$_2$ ☐$_3$ ☐$_{77}$	
RBC		pc/μl	☐$_1$ ☐$_2$ ☐$_3$ ☐$_{77}$	
WBC		pc/μl	☐$_1$ ☐$_2$ ☐$_3$ ☐$_{77}$	
HCG	☐$_0$ Negative ☐$_2$ Positive ☐$_3$ Not application ☐$_{77}$ Not done, reason______			
**Stool routine**	**Sample date: |_|_|_|_|year|_|_|month|_|_|day**			**Abnormalities**
WBC		Qualitative	☐$_1$ ☐$_2$ ☐$_3$ ☐$_{77}$	
OB		Qualitative	☐$_1$ ☐$_2$ ☐$_3$ ☐$_{77}$	
**Biochemical test**	**Sample date: |_|_|_|_|year|_|_|month|_|_|day**			**Abnormalities**
ALT		U/L	☐$_1$ ☐$_2$ ☐$_3$ ☐$_{77}$	
AST		U/L	☐$_1$ ☐$_2$ ☐$_3$ ☐$_{77}$	
BUN		mmol/L	☐$_1$ ☐$_2$ ☐$_3$ ☐$_{77}$	
Scr		μmol/L	☐$_1$ ☐$_2$ ☐$_3$ ☐$_{77}$	
UA		μmol/L	☐$_1$ ☐$_2$ ☐$_3$ ☐$_{77}$	
**Blood glucose**	**Sample date: |_|_|_|_|year|_|_|month|_|_|day**			**Abnormalities**
FBG		mmol/L	☐$_1$ ☐$_2$ ☐$_3$ ☐$_{77}$	

Note: ☐$_1$ Normal; ☐$_2$ Abnormal without clinical significance; ☐$_3$ Abnormal with clinical significance; ☐$_{77}$ Not done.

Observing physicians__________________ Date _ _ _ _ year _ _ month _ _ day

Electrocardiogram (ECG)

Whether to test	☐1 Yes ☐0 No	
Testing date	|_|_|_|_|year|_|_|month|_|_|day	
Testing position	☐1 Sitting ☐2 Standing ☐3 Supine ☐88 Others.	
Description of ECG results		
Determination of ECG clinical significance	☐1 Normal ☐2 Abnormal without clinical significance<br>☐3 Abnormal with clinical significance	
	Description of abnormalities:	

Observing physicians_________________ Date _ _ _ _ year _ _ month _ _ day

Study No. A00908	Center No. |_|_|	Name Initial |_|_|_|_|	Subject No. |_|_|_|	Visit Date |_|_|_|_|year|_|_|month|_|_|day	Visit |2|	Treatment Period

Combination Medication/Treatment

Have there been any new combined medications/non-medications or any changes in previous combined medications/non-medications since the last follow-up visit?
☐0 No ☐1 Yes → Please fill in the combined medication/treatment page. P

Composite events of bleeding

Have there been any bleeding events since the last follow-up visit?
☐0 No ☐1 Yes → Please fill in the adverse events page. P37

Adverse Events

Have there been any adverse events since the last follow-up visit?
☐0 No ☐1 Yes → Please fill in the adverse events page. P38-39

Drugs Management

Actual amount of drugs taken	Whether the remaining drugs are fully recovered	Date of recovery	Amount of recovered drugs	Date of distribution	Amount of distributed drugs
______bags	☐ Yes, please fill in the recovering situation.<br>☐ No, the amount of unrecovered bags_________.<br>Reason for not recovering___<br>________________________		_____bags		_____bags

Appointment for next follow-up visit: |_|_|_|_|year|_|_|month|_|_|day

I have verified the completeness and accuracy of the information filled out for this visit.

Signature of the observing physician **Date: |_|_|_|_|year|_|_|month|_|_|day**

Study No. A00908	Center No. |_|_|	Name Initial |_|_|_|_|	Subject No. |_|_|_|	Visit Date |_|_|_|_|year|_|_|month|_|_|day	Visit |3|	Treatment Period

Vital signs

Measurement items	Value	ND	Measurement location
Height (cm)	|_|_|_|. |_|	□77	□99 NA
Weight (Kg)	|_|_|_|. |_|	□77	□99 NA
BMI(Kg/m^2)	|_|_|. |_|	□77	□99 NA
Breathing (times/min)	|_|_|	□77	□99 NA
HR (beats/min)	|_|_|_|	□77	□1 Brachial artery □2 Carotid artery □3 Radial artery
Temperature (℃)	|_|_|. |_|	□77	□4 Axillary □5 Ear
SBP (mmHg)	|_|_|_|	□77	□1 Brachial artery □2 Ankle
DBP (mmHg)	|_|_|_|	□77	□1 Brachial artery □2 Ankle

Note: ND: Not done; HR: Resting heart rate; SBP: Systolic blood pressure; DBP: Diastolic blood pressure.

Physical examination

Items	Examination results	Abnormal (please specify)
Heart examination	□1 Normal<br>□2 Abnormal<br>□77 Not done	
Others	□1 Normal<br>□2 Abnormal<br>□77 Not done	

<table>
<tr><td>Study No.
A00908</td><td>Center No.
|_|_|</td><td>Name Initial
|_|_|_|_|</td><td>Subject No.
|_|_|_|</td><td>Visit Date
|_|_|_|_|year|_|_|month|_|_|day</td><td>Visit
|3|</td><td>Treatment
Period</td></tr>
</table>

Seattle Angina Questionnaire

Q1: Over the past 4 weeks, the following levels have been limited due to chest pain, chest tightness, and angina

	Severely limited	Moderately limited	Mildly limited	Slightly limited	Not limited	Limited by Other reasons
Dress yourself	☐1	☐2	☐3	☐4	☐5	☐6
Walk indoors	☐1	☐2	☐3	☐4	☐5	☐6
Shower	☐1	☐2	☐3	☐4	☐5	☐6
Climb or stairs (going up three floors without stopping)	☐1	☐2	☐3	☐4	☐5	☐6
Outdoor activities or debris picking	☐1	☐2	☐3	☐4	☐5	☐6
Easy walk (1 km)	☐1	☐2	☐3	☐4	☐5	☐6
Jogging (1 km)	☐1	☐2	☐3	☐4	☐5	☐6
Lift or move heavy objects	☐1	☐2	☐3	☐4	☐5	☐6
Strenuous exercise (such as swimming or playing ball)	☐1	☐2	☐3	☐4	☐5	☐6

Q2: Compared to 4 weeks ago, episodes of chest pain, chest tightness and angina when maximum intensity activity was performed.

☐1 Significant increase ☐2 Slight increase ☐3 Same ☐4 Slight decrease ☐5 Significant decrease

Q3: Over the past 4 weeks, average numbers of episodes of chest pain, chest tightness, and angina.

☐1 ≥4times/day ☐2 1-3 times/day ☐3 ≥3times/week ☐4 1-2times/week ☐5 <1times/week ☐6 No episode

Q4: Over the past 4 weeks, average numbers of times nitro drugs (such as nitroglycerin) were taken for chest pain, chest tightness, and angina.

☐1 ≥4times/day ☐2 1-3 times/day ☐3 ≥3times/week ☐4 1-2times/week ☐5 <1times/week ☐6 No use

Q5: Worries caused by chest pain, chest tightness, and angina that require medication as prescribed.

☐1 Severe ☐2 Moderate ☐3 Mild ☐4 Rare ☐5 None ☐6 Physician not administered

Q6: Satisfaction degree with various measures for the treatment of chest pain, chest tightness and angina.

☐1 Dissatisfied ☐2 Mostly dissatisfied ☐3 Partially satisfied ☐4 Mostly satisfied ☐5 Highly satisfied

Q7: Satisfaction degree with the doctor's explanations for chest pain, chest tightness, and angina.

☐1 Dissatisfied ☐2 Mostly dissatisfied ☐3 Partially satisfied ☐4 Mostly satisfied ☐5 Highly satisfied

Q8: Overall satisfaction degree with current treatment of chest pain, chest tightness, and angina.
☐1 Dissatisfied ☐2 Mostly dissatisfied ☐3 Partially satisfied ☐4 Mostly satisfied ☐5 Highly satisfied

Q9: Over the past 4 weeks, the extent to which chest pain, chest tightness and angina affected the joy of life.
☐1 Dissatisfied ☐2 Mostly dissatisfied ☐3 Partially satisfied ☐4 Mostly satisfied ☐5 Highly satisfied

Q10: How would you feel if you still had chest pain, chest tightness and angina in your future life?☐1 Dissatisfied ☐2 Mostly dissatisfied ☐3 Partially satisfied ☐4 Mostly satisfied ☐5 Highly satisfied

Q11: The degree of worry about heart attacks and sudden deaths.
☐1 Worried all the time ☐2 Worried often ☐3 Worried sometimes ☐4 Rarely worried ☐5 Never worried

Note: Standard Scores = (actual score - minimum score in this domain) / (highest score in this domain - minimum score in this domain)

- **Score of physical activity limitation (Q 1):**
- **Score of angina steady-state (Q 2):**
- **Score of angina attacks (Q3-Q4):**
- **Score of treatment satisfaction (Q5-Q8):**
- **Score of disease awareness (Q9-Q11):**

Combination Medication/Treatment

**Have there been any new combined medications/non-medications or any changes in previous combined medications/non-medications since the last follow-up visit?**<br>**□0 No □1 Yes → Please fill in the combined medication/treatment page. P**

Composite events of bleeding

**Have there been any bleeding events since the last follow-up visit?**<br>**□0 No □1 Yes → Please fill in the adverse events page. P37**

Adverse Events

**Have there been any adverse events since the last follow-up visit?**<br>**□0 No □1 Yes → Please fill in the adverse events page. P38-39**

Drugs Management

Actual amount of drugs taken	Whether the remaining drugs are fully recovered	Date of recovery	Amount of recovered drugs	Date of distribution	Amount of distributed drugs
______bags	☐ Yes, please fill in the recovering situation.<br>☐ No, the amount of unrecovered bags_________.<br>Reason for not recovering___<br>________________________		_____bags		_____bags

Appointment for next follow-up visit: |_|_|_|_|year|_|_|month|_|_|day

**I have verified the completeness and accuracy of the information filled out for this visit.**<br>**Signature of the observing physician Date: |_|_|_|_|year|_|_|month|_|_|day**

Vital signs

**Measurement items**	**Value**	**ND**	**Measurement location**
Height (cm)	|_|_|_|. |_|	☐77	☐99 NA
Weight (Kg)	|_|_|_|. |_|	☐77	☐99 NA
BMI(Kg/m^2)	|_|_|. |_|	☐77	☐99 NA
Breathing (times/min)	|_|_|	☐77	☐99 NA
HR (beats/min)	|_|_|_|	☐77	☐1 Brachial artery ☐2 Carotid artery ☐3 Radial artery
Temperature (℃)	|_|_|. |_|	☐77	☐4 Axillary ☐5 Ear
SBP (mmHg)	|_|_|_|	☐77	☐1 Brachial artery ☐2 Ankle
DBP (mmHg)	|_|_|_|	☐77	☐1 Brachial artery ☐2 Ankle

Note: ND: Not done; HR: Resting heart rate; SBP: Systolic blood pressure; DBP: Diastolic blood pressure.

Physical examination

**Items**	**Examination results**	**Abnormal (please specify)**
Heart examination	☐1 Normal<br>☐2 Abnormal<br>☐77 Not done	
Others	☐1 Normal<br>☐2 Abnormal<br>☐77 Not done	

<table>
<tr><td>Study No.
A00908</td><td>Center No.
|_|_|</td><td>Name Initial
|_|_|_|_|</td><td>Subject No.
|_|_|_|</td><td>Visit Date
|_|_|_|_|year|_|_|month|_|_|day</td><td>Visit
|4|</td><td>Treatment
Period</td></tr>
</table>

Seattle Angina Questionnaire

Q1: Over the past 4 weeks, the following levels have been limited due to chest pain, chest tightness, and angina

	Severely limited	Moderately limited	Mildly limited	Slightly limited	Not limited	Limited by Other reasons
Dress yourself	☐$_1$	☐$_2$	☐$_3$	☐$_4$	☐$_5$	☐$_6$
Walk indoors	☐$_1$	☐$_2$	☐$_3$	☐$_4$	☐$_5$	☐$_6$
Shower	☐$_1$	☐$_2$	☐$_3$	☐$_4$	☐$_5$	☐$_6$
Climb or stairs (going up three floors without stopping)	☐$_1$	☐$_2$	☐$_3$	☐$_4$	☐$_5$	☐$_6$
Outdoor activities or debris picking	☐$_1$	☐$_2$	☐$_3$	☐$_4$	☐$_5$	☐$_6$
Easy walk (1 km)	☐$_1$	☐$_2$	☐$_3$	☐$_4$	☐$_5$	☐$_6$
Jogging (1 km)	☐$_1$	☐$_2$	☐$_3$	☐$_4$	☐$_5$	☐$_6$
Lift or move heavy objects	☐$_1$	☐$_2$	☐$_3$	☐$_4$	☐$_5$	☐$_6$
Strenuous exercise (such as swimming or playing ball)	☐$_1$	☐$_2$	☐$_3$	☐$_4$	☐$_5$	☐$_6$

Q2: Compared to 4 weeks ago, episodes of chest pain, chest tightness and angina when maximum intensity activity was performed.
☐$_1$ Significant increase ☐$_2$ Slight increase ☐$_3$ Same ☐$_4$ Slight decrease ☐$_5$ Significant decrease

Q3: Over the past 4 weeks, average numbers of episodes of chest pain, chest tightness, and angina.
☐$_1$ ≥4times/day ☐$_2$ 1-3 times/day ☐$_3$ ≥3times/week ☐$_4$ 1-2times/week ☐$_5$ <1times/week ☐$_6$ No episode

Q4: Over the past 4 weeks, average numbers of times nitro drugs (such as nitroglycerin) were taken for chest pain, chest tightness, and angina.
☐$_1$ ≥4times/day ☐$_2$ 1-3 times/day ☐$_3$ ≥3times/week ☐$_4$ 1-2times/week ☐$_5$ <1times/week ☐$_6$ No use

Q5: Worries caused by chest pain, chest tightness, and angina that require medication as prescribed.
☐$_1$ Severe ☐$_2$ Moderate ☐$_3$ Mild ☐$_4$ Rare ☐$_5$ None ☐$_6$ Physician not administered

Q6: Satisfaction degree with various measures for the treatment of chest pain, chest tightness and angina.
☐$_1$ Dissatisfied ☐$_2$ Mostly dissatisfied ☐$_3$ Partially satisfied ☐$_4$ Mostly satisfied ☐$_5$ Highly satisfied

Q7: Satisfaction degree with the doctor's explanations for chest pain, chest tightness, and angina.
☐$_1$ Dissatisfied ☐$_2$ Mostly dissatisfied ☐$_3$ Partially satisfied ☐$_4$ Mostly satisfied ☐$_5$ Highly satisfied

Q8: Overall satisfaction degree with current treatment of chest pain, chest tightness, and angina.
□$_1$ Dissatisfied □$_2$ Mostly dissatisfied □$_3$ Partially satisfied □$_4$ Mostly satisfied □$_5$ Highly satisfied

Q9: Over the past 4 weeks, the extent to which chest pain, chest tightness and angina affected the joy of life.
□$_1$ Dissatisfied □$_2$ Mostly dissatisfied □$_3$ Partially satisfied □$_4$ Mostly satisfied □$_5$ Highly satisfied

Q10: How would you feel if you still had chest pain, chest tightness and angina in your future life?□$_1$ Dissatisfied □$_2$ Mostly dissatisfied □$_3$ Partially satisfied □$_4$ Mostly satisfied □$_5$ Highly satisfied

Q11: The degree of worry about heart attacks and sudden deaths.
□$_1$ Worried all the time □$_2$ Worried often □$_3$ Worried sometimes □$_4$ Rarely worried □$_5$ Never worried

Note: Standard Scores = (actual score - minimum score in this domain) / (highest score in this domain - minimum score in this domain)

- **Score of physical activity limitation (Q 1):**
- **Score of angina steady-state (Q 2):**
- **Score of angina attacks (Q3-Q4):**
- **Score of treatment satisfaction (Q5-Q8):**
- **Score of disease awareness (Q9-Q11):**

Coronary CTA **(Inspection date: |_|_|_|_|year|_|_|month|_|_|day)**

Lesioned vessels		FFR (CT)	DS (%)	AS (%)	Gensini Score	CACS	Image quality*
LM							☐1 ☐2 ☐3
LAD	prox						☐1 ☐2 ☐3
LAD	mid						☐1 ☐2 ☐3
LAD	apic						☐1 ☐2 ☐3
	1st D						☐1 ☐2 ☐3
	2nd D						☐1 ☐2 ☐3
LCX	prox						☐1 ☐2 ☐3
LCX	apic						☐1 ☐2 ☐3
	OM						☐1 ☐2 ☐3
	PD						☐1 ☐2 ☐3
	PL						☐1 ☐2 ☐3
RCA	prox						☐1 ☐2 ☐3
RCA	mid						☐1 ☐2 ☐3
RCA	dist						☐1 ☐2 ☐3
	PD						☐1 ☐2 ☐3
Totaol Score							☐1 ☐2 ☐3
Description of coronary CTA results							

Note: FFR(CT): Coronary computed tomography angiography-derived fractional flow reserve; DS(%): Percentage of diameter stenosis; % AS: Percentage of area stenosis; CACS: Coronary artery calcification score; *Image quality: ☐1 Excellent, ☐2 Fair, ☐3 Poor.

Observing physicians__________________ Date _ _ _ _ year _ _ month _ _ day

Study No. A00908	Center No. |_|_|	Name Initial |_|_|_|_|	Subject No. |_|_|_|	Visit Date |_|_|_|_|year|_|_|month|_|_|day	Visit |4|	Treatment Period

Carotid Artery Ultrasound

Whether to test	☐1 Yes ☐0 No	
Testing date	|_|_|_|_|year|_|_|month|_|_|day	
	Left carotid artery	Right carotid artery
Carotid intima-media thickness (IMT) (mm)		
Is plaque detected	☐1Yes ☐0 No	☐1Yes ☐0 No
Carotid plaque length × thickness (longitudinal section) (mm)		
Plaque type	☐1Vulnerable ☐0Non-vulnerable	☐1Vulnerable ☐0Non-vulnerable
Degree of carotid lumen stenosis (%)		
Description of carotid ultrasound results		

Study No. A00908	Center No. |_|_|	Name Initial |_|_|_|_|	Subject No. |_|_|_|	Visit Date |_|_|_|_|year|_|_|month|_|_|day	Visit |4|	Treatment Period

Blood lipids (Sample date: |_|_|_|_|year|_|_|month|_|_|day)

Indicators	Test value	Standard unit	Clinical significance determination	
TC		mmol/L	☐1 ☐2 ☐3 ☐77	
TG		mmol/L	☐1 ☐2 ☐3 ☐77	
HDL-C		mmol/L	☐1 ☐2 ☐3 ☐77	
LDL-C		mmol/L	☐1 ☐2 ☐3 ☐77	
Apo A1		g/L	☐1 ☐2 ☐3 ☐77	
Apo B		g/L	☐1 ☐2 ☐3 ☐77	
Lp(a)		nmol/L	☐1 ☐2 ☐3 ☐77	

Note: ☐1 Normal; ☐2 Abnormal without clinical significance; ☐3 Abnormal with clinical significance; ☐77 Not done.

Inflammatory factors (Sample date: |_|_|_|_|year|_|_|month|_|_|day)

Indicators	Test value	Standard unit	Clinical significance determination	
hs-CRP		mg/L	☐1 ☐2 ☐3 ☐77	
MMP-9		ng/mL	☐1 ☐2 ☐3 ☐77	

Note: ☐1 Normal; ☐2 Abnormal without clinical significance; ☐3 Abnormal with clinical significance; ☐77 Not done.

Observing physicians__________________ Date _ _ _ _ year _ _ month _ _ day

Laboratory tests

<table>
<tr><th>Indicators</th><th>Test value</th><th>Standard unit</th><th colspan="2">Clinical significance determination</th></tr>
<tr><td>Blood routine</td><td colspan="3">Sample date: |_|_|_|_|year|_|_|month|_|_|day</td><td>Abnormalities</td></tr>
<tr><td>RBC</td><td></td><td>$\times 10^{12}$/L</td><td>☐$_1$ ☐$_2$ ☐$_3$ ☐$_{77}$</td><td></td></tr>
<tr><td>HBG</td><td></td><td>g/L</td><td>☐$_1$ ☐$_2$ ☐$_3$ ☐$_{77}$</td><td></td></tr>
<tr><td>WBC</td><td></td><td>$\times 10^9$/L</td><td>☐$_1$ ☐$_2$ ☐$_3$ ☐$_{77}$</td><td></td></tr>
<tr><td>NEUT%</td><td></td><td>%</td><td>☐$_1$ ☐$_2$ ☐$_3$ ☐$_{77}$</td><td></td></tr>
<tr><td>PLT</td><td></td><td>$\times 10^9$/L</td><td>☐$_1$ ☐$_2$ ☐$_3$ ☐$_{77}$</td><td></td></tr>
<tr><td>Coagulation</td><td colspan="3">Sample date: |_|_|_|_|year|_|_|month|_|_|day</td><td>Abnormalities</td></tr>
<tr><td>PT</td><td></td><td></td><td>☐$_1$ ☐$_2$ ☐$_3$ ☐$_{77}$</td><td></td></tr>
<tr><td>TT</td><td></td><td></td><td>☐$_1$ ☐$_2$ ☐$_3$ ☐$_{77}$</td><td></td></tr>
<tr><td>INR</td><td></td><td></td><td>☐$_1$ ☐$_2$ ☐$_3$ ☐$_{77}$</td><td></td></tr>
<tr><td>APTT</td><td></td><td></td><td>☐$_1$ ☐$_2$ ☐$_3$ ☐$_{77}$</td><td></td></tr>
<tr><td>fibrinogen</td><td></td><td></td><td>☐$_1$ ☐$_2$ ☐$_3$ ☐$_{77}$</td><td></td></tr>
<tr><td>Urine routine</td><td colspan="3">Sample date: |_|_|_|_|year|_|_|month|_|_|day</td><td>Abnormalities</td></tr>
<tr><td>PRO</td><td></td><td>Qualitative</td><td>☐$_1$ ☐$_2$ ☐$_3$ ☐$_{77}$</td><td></td></tr>
<tr><td>GLU</td><td></td><td>Qualitative</td><td>☐$_1$ ☐$_2$ ☐$_3$ ☐$_{77}$</td><td></td></tr>
<tr><td>RBC</td><td></td><td>pc/μl</td><td>☐$_1$ ☐$_2$ ☐$_3$ ☐$_{77}$</td><td></td></tr>
<tr><td>WBC</td><td></td><td>pc/μl</td><td>☐$_1$ ☐$_2$ ☐$_3$ ☐$_{77}$</td><td></td></tr>
<tr><td>HCG</td><td colspan="4">☐$_0$ Negative ☐$_2$ Positive ☐$_3$ Not application ☐$_{77}$ Not done, reason______</td></tr>
<tr><td>Stool routine</td><td colspan="3">Sample date: |_|_|_|_|year|_|_|month|_|_|day</td><td>Abnormalities</td></tr>
<tr><td>WBC</td><td></td><td>Qualitative</td><td>☐$_1$ ☐$_2$ ☐$_3$ ☐$_{77}$</td><td></td></tr>
<tr><td>OB</td><td></td><td>Qualitative</td><td>☐$_1$ ☐$_2$ ☐$_3$ ☐$_{77}$</td><td></td></tr>
<tr><td>Biochemical test</td><td colspan="3">Sample date: |_|_|_|_|year|_|_|month|_|_|day</td><td>Abnormalities</td></tr>
<tr><td>ALT</td><td></td><td>U/L</td><td>☐$_1$ ☐$_2$ ☐$_3$ ☐$_{77}$</td><td></td></tr>
<tr><td>AST</td><td></td><td>U/L</td><td>☐$_1$ ☐$_2$ ☐$_3$ ☐$_{77}$</td><td></td></tr>
<tr><td>BUN</td><td></td><td>mmol/L</td><td>☐$_1$ ☐$_2$ ☐$_3$ ☐$_{77}$</td><td></td></tr>
<tr><td>Scr</td><td></td><td>μmol/L</td><td>☐$_1$ ☐$_2$ ☐$_3$ ☐$_{77}$</td><td></td></tr>
<tr><td>UA</td><td></td><td>μmol/L</td><td>☐$_1$ ☐$_2$ ☐$_3$ ☐$_{77}$</td><td></td></tr>
<tr><td>Blood glucose</td><td colspan="3">Sample date: |_|_|_|_|year|_|_|month|_|_|day</td><td>Abnormalities</td></tr>
<tr><td>FBG</td><td></td><td>mmol/L</td><td>☐$_1$ ☐$_2$ ☐$_3$ ☐$_{77}$</td><td></td></tr>
</table>

Note: ☐$_1$ Normal; ☐$_2$ Abnormal without clinical significance; ☐$_3$ Abnormal with clinical significance; ☐$_{77}$ Not done.

Observing physicians__________________ Date _ _ _ _ year _ _ month _ _ day

Study No. A00908	Center No. |_|_|	Name Initial |_|_|_|_|	Subject No. |_|_|_|	Visit Date |_|_|_|_|year|_|_|month|_|_|day	Visit |4|	Treatment Period

Electrocardiogram (ECG)

Whether to test	☐1 Yes ☐0 No
Testing date	|_|_|_|_|year|_|_|month|_|_|day
Testing position	☐1 Sitting ☐2 Standing ☐3 Supine ☐88 Others.
Description of ECG results	
Determination of ECG clinical significance	☐1 Normal ☐2 Abnormal without clinical significance<br>☐3 Abnormal with clinical significance
	Description of abnormalities:

Observing physicians__________________ Date _ _ _ _ year _ _ month _ _ day

Study No. A00908	Center No. |_|_|	Name Initial |_|_|_|_|	Subject No. |_|_|_|	Visit Date |_|_|_|_|year|_|_|month|_|_|day	Visit |4|	Treatment Period

Sticky Place of Inspection Report Forms for Treatment Period

Study No. A00908	Center No. |_|_|	Name Initial |_|_|_|_|	Subject No. |_|_|_|	Visit Date |_|_|_|_|year|_|_|month|_|_|day	Visit |4|	Treatment Period

Combination Medication/Treatment

**Have there been any new combined medications/non-medications or any changes in previous combined medications/non-medications since the last follow-up visit?**<br>**☐0 No ☐1 Yes → Please fill in the combined medication/treatment page. P**

Composite events of bleeding

**Have there been any bleeding events since the last follow-up visit?**<br>**☐0 No ☐1 Yes → Please fill in the adverse events page. P37**

Adverse Events

**Have there been any adverse events since the last follow-up visit?**<br>**☐0 No ☐1 Yes → Please fill in the adverse events page. P38-39**

Drugs Management

Actual amount of drugs taken	Whether the remaining drugs are fully recovered	Date of recovery	Amount of recovered drugs	Date of distribution	Amount of distributed drugs
______bags	☐ Yes, please fill in the recovering situation.<br>☐ No, the amount of unrecovered bags_________.<br>Reason for not recovering___<br>_________________________		_____bags		_____bags

**I have verified the completeness and accuracy of the information filled out for this visit.**
**Signature of the observing physician** **Date: |_|_|_|_|year|_|_|month|_|_|day**

Composite events of bleeding

Event No.	
Discovery Date	|_|_|_|_|year|_|_|month|_|_|day
Bleeding type	☐1 Type 2<br>☐2 Type 3a ☐3 Type 3b ☐4 Type 3c<br>☐5 Type 5a ☐6 Type 5b
Description of the course of the bleeding event (including symptoms, signs, clinical tests, etc.) and its treatment:	

Note:

Type 2: any obvious, actionable signs of bleeding that meet at least one of the following criteria: (1) requires non-surgical, medical professional intervention, (2) results in hospitalization or improved care, or (3) prompts evaluation.

Type 3a: Overt bleeding plus hemoglobin decreased by 3 ~ 5 g/dL* (decreased hemoglobin related to bleeding), or plus transfusion.

Type 3b: Overt bleeding plus a decrease in hemoglobin by 5 g/dL * (decreased hemoglobin related to bleeding), or cardiac tamponade, or bleeding requiring surgical intervention to control, or bleeding requiring intravenous vasoactive drugs.

Type 3c: Intracranial hemorrhage, or subcategories confirmed by autopsy or imaging or lumbar puncture, or intraocular bleed compromising vision.

Type 5a: Probable fatal bleeding; no autopsy or imaging confirmation but clinically suspicious

Type 5a: Definite fatal bleeding: overt bleeding, or confirmed by autopsy or imaging.

Observing physicians__________________ Date _ _ _ _ year _ _ month _ _ day

Adverse Event (AE)

(Using standard medical terms) Record all observations and use the question, “How have you felt differently since your last examination?” Record the name of the diagnosis if there is a definite diagnosis, or the name of the symptom if there is no definite diagnosis. Record one adverse event per sheet.

Did the subject have any adverse events during the study?

☐$_0$ No ☐$_1$ Yes → Please fill in the combined medication form.

<table>
<tr><td>AE No.</td><td colspan="2"></td><td>AE Name</td><td></td></tr>
<tr><td>Discovery Date</td><td colspan="2">|_|_|_|_|year|_|_|month|_|_|day</td><td>End Date</td><td>|_|_|_|_|year|_|_|month|_|_|day</td></tr>
<tr><td>AE Degree*</td><td colspan="4">☐$_1$ Mild ☐$_2$ Moderate ☐$_3$ Severe</td></tr>
<tr><td>Whether SAE</td><td colspan="4">☐$_0$ No ☐$_1$ Yes → (Please fill in the SAE report form)</td></tr>
<tr><td>SAE Status</td><td colspan="4">☐$_1$ Resulting in hospitalization ☐$_2$ Extending hospitalization ☐$_3$ Disability ☐$_4$ Functional disorder ☐$_5$ Resulting in congenital malformation ☐$_6$ Life-threatening or death ☐$_{88}$ Others</td></tr>
<tr><td>Measures taken for test drugs</td><td colspan="4">☐$_1$ Increased dose ☐$_2$ Unchanged dose ☐$_3$ Decreased dose ☐$_4$ Discontinued ☐$_5$ Terminated ☐$_{99}$ No Applicable ☐$_{77}$ Unknown</td></tr>
<tr><td>AE regression</td><td colspan="4">☐$_1$ Death ☐$_2$ Uncured/unremitted ☐$_3$ Cured ☐$_4$ Symptoms disappeared but with sequelae ☐$_5$ Remitted ☐$_{66}$ Unknown</td></tr>
<tr><td>Whether symptomatic treatment</td><td colspan="4">☐$_0$ No
☐$_1$ Nom-medication→ (fill in the combined medication/treatment form)
☐$_2$ Medication→ (fill in the combined medication/treatment form)</td></tr>
<tr><td>Whether AE broke the blind</td><td>☐$_0$ No ☐$_1$ Yes</td><td colspan="2">Did patients with-draw because of it</td><td>☐$_0$ No ☐$_1$ Yes</td></tr>
<tr><td>The association between AE and test drugs</td><td colspan="4">☐$_1$ Definitely ☐$_2$ Most likely ☐$_3$ Probably ☐$_4$ Probably not relevant
☐$_5$ To be evaluated ☐$_6$ Unable to evaluate</td></tr>
<tr><td colspan="5">Description of the course of the adverse reaction/event (including symptoms, signs, clinical tests, etc.) and its treatment:</td></tr>
</table>

* AE Degree: Mild: tolerated by the subject, not affecting treatment, not requiring special treatment, without effect on the subject's recovery. Moderate: intolerable to the subject, requiring, withdrawal of the drug or special treatment, with direct effect on the subject's recovery. Severe: life-threatening, lethal or disabling to the subject, requiring immediate withdrawal of the drug or emergency treatment.

Note: For serious adverse events, please fill out the Serious Adverse Event Report Form and report according to the prescribed procedures.

Adverse Event (AE)

Did the subject have any adverse events during the study?

□$_0$ No □$_1$ Yes → Please fill in the combined medication form.

**AE No.**		**AE Name**	
**Discovery Date**	|_|_|_|_|year|_|_|month|_|_|day	**End Date**	|_|_|_|_|year|_|_|month|_|_|day
**AE Degree***	□$_1$ Mild □$_2$ Moderate □$_3$ Severe		
**Whether SAE**	□$_0$ No □$_1$ Yes → (Please fill in the SAE report form)		
**SAE Status**	□$_1$ Resulting in hospitalization □$_2$ Extending hospitalization □$_3$ Disability □$_4$ Functional disorder □$_5$ Resulting in congenital malformation □$_6$ Life-threatening or death □$_{88}$ Others		
**Measures taken for test drugs**	□$_1$ Increased dose □$_2$ Unchanged dose □$_3$ Decreased dose □$_4$ Discontinued □$_5$ Terminated □$_{99}$ No Applicable □$_{77}$ Unknown		
**AE regression**	□$_1$ Death □$_2$ Uncured/unremitted □$_3$ Cured □$_4$ Symptoms disappeared but with sequelae □$_5$ Remitted □$_{66}$ Unknown		
**Whether symptomatic treatment**	□$_0$ No<br>□$_1$ Nom-medication→ (fill in the combined medication/treatment form)<br>□$_2$ Medication→ (fill in the combined medication/treatment form)		
**Whether AE broke the blind**	□$_0$ No □$_1$ Yes	**Did patients with-draw because of it**	□$_0$ No □$_1$ Yes
**The association between AE and test drugs**	□$_1$ Definitely □$_2$ Most likely □$_3$ Probably □$_4$ Probably not relevant □$_5$ Unlikely to be relevant		
Description of the course of the adverse reaction/event (including symptoms, signs, clinical tests, etc.) and its treatment:			

* AE Degree: Mild: tolerated by the subject, not affecting treatment, not requiring special treatment, without effect on the subject's recovery. Moderate: intolerable to the subject, requiring, withdrawal of the drug or special treatment, with direct effect on the subject's recovery. Severe: life-threatening, lethal or disabling to the subject, requiring immediate withdrawal of the drug or emergency treatment.

Note: For serious adverse events, please fill out the Serious Adverse Event Report Form and report according to the prescribed procedures.

Observing physicians__________________ Date _ _ _ _ year _ _ month _ _ day

Major Adverse Cardiovascular Events (MACE)

<table>
<tr><td>MACE No.</td><td colspan="3"></td></tr>
<tr><td>Discovery Date</td><td>|_|_|_|_|year|_|_|month|_|_|day</td><td>End Date</td><td>|_|_|_|_|year|_|_|month|_|_|day</td></tr>
<tr><td>MACE Degree*</td><td colspan="3">☐$_{1}$ Mild ☐$_{2}$ Moderate ☐$_{3}$ Severe</td></tr>
<tr><td>MACE Classification</td><td colspan="3">☐$_{1}$ All-cause death ☐$_{2}$ Non-fatal myocardial infarction ☐$_{3}$ Stroke
☐$_{4}$ Revascularization (PCI) ☐$_{5}$ Revascularization (CABG)
☐$_{6}$ Rehospitalization due to ACS or heart failure</td></tr>
<tr><td>MACE Regression</td><td colspan="3">☐$_{1}$ Death, time of death: |_|_|_|_|year|_|_|month|_|_|day
☐$_{2}$ Uncured/unremitted ☐$_{3}$ Cured ☐$_{4}$ Symptoms disappeared but with sequelae ☐$_{5}$ Remitted ☐$_{6}$ Unknown</td></tr>
<tr><td>Whether symptomatic treatment</td><td colspan="3">☐$_{0}$ No ☐$_{1}$ Yes</td></tr>
<tr><td>Did patients withdraw because of it</td><td colspan="3">☐$_{0}$ No ☐$_{1}$ Yes</td></tr>
</table>

* MACE Degree: Mild: tolerated by the subject, not affecting treatment, noting require special treatment, without effect on the subject's recovery. Moderate: intolerable to the subject, requiring treatment, with direct effect on the subject's recovery. Severe: life-threatening, lethal or disabling to the subject, requiring emergency treatment.
Note: For serious adverse events, please fill out the Serious Adverse Event Report Form and report according to the prescribed procedures.

I have verified the completeness and accuracy of the information filled out for this visit.

Signature of the observing physician **Date: |_|_|_|_|year|_|_|month|_|_|day**

Study No. A00908	Center No. |_|_|	Name Initial |_|_|_|_|	Screening No. |_|_|_|	Study Period (12 months)
				Combined Medication

Combined Medication/Treatment

Did the subject have any new combined medications/non-medications or any changes in pre-study medications/non-medications during the follow-up?

☐$_0$ No ☐$_1$ Yes → Please fill in the table below.

No	Medication or non-medication name①	Single dose	Dose unit②	Fre-quency③	Administra-tion route④	Indication of use	Start date⑤	End date⑥ or still in use
1							|_|_|_|_|year|_|_|month|_|_|day	|_|_|_|_|year|_|_|month|_|_|day or go on ☐$_1$
2							|_|_|_|_|year|_|_|month|_|_|day	|_|_|_|_|year|_|_|month|_|_|day or go on ☐$_1$
3							|_|_|_|_|year|_|_|month|_|_|day	|_|_|_|_|year|_|_|month|_|_|day or go on ☐$_1$
4							|_|_|_|_|year|_|_|month|_|_|day	|_|_|_|_|year|_|_|month|_|_|day or go on ☐$_1$
5							|_|_|_|_|year|_|_|month|_|_|day	|_|_|_|_|year|_|_|month|_|_|day or go on ☐$_1$
6							|_|_|_|_|year|_|_|month|_|_|day	|_|_|_|_|year|_|_|month|_|_|day or go on ☐$_1$
7							|_|_|_|_|year|_|_|month|_|_|day	|_|_|_|_|year|_|_|month|_|_|day or go on ☐$_1$
8							|_|_|_|_|year|_|_|month|_|_|day	|_|_|_|_|year|_|_|month|_|_|day or go on ☐$_1$
9							|_|_|_|_|year|_|_|month|_|_|day	|_|_|_|_|year|_|_|month|_|_|day or go on ☐$_1$

<table>
<tr><td rowspan="2">Study No.
A00908</td><td rowspan="2">Center No.
|_|_|</td><td rowspan="2">Name Initial
|_|_|_|_|</td><td rowspan="2">Screening No.
|_|_|_|</td><td>Study Period (12 months)</td></tr>
<tr><td>Combined Medication</td></tr>
</table>

<table>
<tr><td>10</td><td></td><td></td><td></td><td></td><td></td><td></td><td>|_|_|_|_|year|_|_|month|_|_|day</td><td>|_|_|_|_|year|_|_|month|_|_|day or go on ☐1</td></tr>
<tr><td>11</td><td></td><td></td><td></td><td></td><td></td><td></td><td>|_|_|_|_|year|_|_|month|_|_|day</td><td>|_|_|_|_|year|_|_|month|_|_|day or go on ☐1</td></tr>
<tr><td>12</td><td></td><td></td><td></td><td></td><td></td><td></td><td>|_|_|_|_|year|_|_|month|_|_|day</td><td>|_|_|_|_|year|_|_|month|_|_|day or go on ☐1</td></tr>
<tr><td>13</td><td></td><td></td><td></td><td></td><td></td><td></td><td>|_|_|_|_|year|_|_|month|_|_|day</td><td>|_|_|_|_|year|_|_|month|_|_|day or go on ☐1</td></tr>
<tr><td>14</td><td></td><td></td><td></td><td></td><td></td><td></td><td>|_|_|_|_|year|_|_|month|_|_|day</td><td>|_|_|_|_|year|_|_|month|_|_|day or go on ☐1</td></tr>
<tr><td>15</td><td></td><td></td><td></td><td></td><td></td><td></td><td>|_|_|_|_|year|_|_|month|_|_|day</td><td>|_|_|_|_|year|_|_|month|_|_|day or go on ☐1</td></tr>
</table>

Remarks: ① If non-medication, fill in NA in the column of Single Dose, Dose Unit and Administration Route.

② Dose unit: 1.mg, 2.ug, 3.mL, 4.g, 5.IU, 6.TABLET, 7.CAPSULE, 8.PUFF, 9.Others, please specify, 10. NA

③ Frequency: 1.BID (twice a day), 2.TID (three times a day), 3.QID (four times a day), 4.QOD (every other day), 5.QM(once a month), 6.PRN(on demand), 7.UNKONWN(unknown), 8.QD(once a day), 9.Others, please specify

④ Administration route: 1.oral, 2.topical, 3.subcutaneous, 4. transdermal, 5.intraocular, 6.intramuscular, 7.respiratory/inhalation, 8.intralesional, 9.intraperitoneal, 10. nasal, 11. vaginal, 12. rectal, 13. sublingual, 14. intravenous injection, 15.Others, please specify, 16. NA

⑤ Please fill in start date and end date as completely as possible. if unclear, "UK" can be filled in.

.

I have verified the completeness and accuracy of the information filled out for this visit.

Signature of the observing physician **Date: |_|_|_|_|year|_|_|month|_|_|day**

Coronary CTA **(Inspection date: |_|_|_|_|year|_|_|month|_|_|day)**

**Lesioned vessels**		**FFR (CT)**	**DS (%)**	**AS (%)**	**Gensini Score**	**CACS**	**Image quality***
LM							☐1 ☐2 ☐3
LAD	prox						☐1 ☐2 ☐3
LAD	mid						☐1 ☐2 ☐3
LAD	apic						☐1 ☐2 ☐3
	1st D						☐1 ☐2 ☐3
	2nd D						☐1 ☐2 ☐3
LCX	prox						☐1 ☐2 ☐3
LCX	apic						☐1 ☐2 ☐3
	OM						☐1 ☐2 ☐3
	PD						☐1 ☐2 ☐3
	PL						☐1 ☐2 ☐3
RCA	prox						☐1 ☐2 ☐3
RCA	mid						☐1 ☐2 ☐3
RCA	dist						☐1 ☐2 ☐3
	PD						☐1 ☐2 ☐3
Totaol Score							☐1 ☐2 ☐3
Description of coronary CTA results							

Note: FFR(CT): Coronary computed tomography angiography-derived fractional flow reserve; DS(%): Percentage of diameter stenosis; % AS: Percentage of area stenosis; CACS: Coronary artery calcification score; *Image quality: ☐1 Excellent, ☐2 Fair, ☐3 Poor.

Study No. A00908	Center No. |_|_|	Name Initial |_|_|_|_|	Subject No. |_|_|_|	Out-of-time visit

Major Adverse Cardiovascular Events (MACE)

**MACE No.**			
**Discovery Date**	|_|_|_|_|year|_|_|month|_|_|day	**End Date**	|_|_|_|_|year|_|_|month|_|_|day
**MACE Degree***	☐1 Mild ☐2 Moderate ☐3 Severe		
**MACE Classification**	☐1 All-cause death ☐2 Non-fatal myocardial infarction ☐3 Stroke ☐4 Revascularization (PCI) ☐5 Revascularization (CABG) ☐6 Rehospitalization due to ACS or heart failure		
**MACE Regression**	☐1 Death, time of death: |_|_|_|_|year|_|_|month|_|_|day ☐2 Uncured/unremitted ☐3 Cured ☐4 Symptoms disappeared but with sequelae ☐5 Remitted ☐6 Unknown		
**Whether symptomatic treatment**	☐0 No ☐1 Yes		
**Did patients withdraw because of it**	☐0 No ☐1 Yes		

* MACE Degree: Mild: tolerated by the subject, not affecting treatment, noting require special treatment, without effect on the subject's recovery. Moderate: intolerable to the subject, requiring treatment, with direct effect on the subject's recovery. Severe: life-threatening, lethal or disabling to the subject, requiring emergency treatment.
Note: For serious adverse events, please fill out the Serious Adverse Event Report Form and report according to the prescribed procedures.

Study No. A00908	Center No. |_|_|	Name Initial |_|_|_|_|	Subject No. |_|_|_|	Out-of-time visit

Adverse Event (AE)

Did the subject have any adverse events during the study?

□$_0$ No □$_1$ Yes → Please fill in the combined medication form.

**AE No.**		**AE Name**	
**Discovery Date**	|_|_|_|_|year|_|_|month|_|_|day	**End Date**	|_|_|_|_|year|_|_|month|_|_|day
**AE Degree***	□$_1$ Mild □$_2$ Moderate □$_3$ Severe		
**Whether SAE**	□$_0$ No □$_1$ Yes → (Please fill in the SAE report form)		
**SAE Status**	□$_1$ Resulting in hospitalization □$_2$ Extending hospitalization □$_3$ Disability □$_4$ Functional disorder □$_5$ Resulting in congenital malformation □$_6$ Life-threatening or death □$_{88}$ Others		
**Measures taken for test drugs**	□$_1$ Increased dose □$_2$ Unchanged dose □$_3$ Decreased dose □$_4$ Discontinued □$_5$ Terminated □$_{99}$ No Applicable □$_{77}$ Unknown		
**AE regression**	□$_1$ Death □$_2$ Uncured/unremitted □$_3$ Cured □$_4$ Symptoms disappeared but with sequelae □$_5$ Remitted □$_{66}$ Unknown		
**Whether symptomatic treatment**	□$_0$ No<br>□$_1$ Nom-medication→ (fill in the combined medication/treatment form)<br>□$_2$ Medication→ (fill in the combined medication/treatment form)		
**Whether AE broke the blind**	□$_0$ No □$_1$ Yes	**Did patients with-draw because of it**	□$_0$ No □$_1$ Yes
**The relationship between AE and the test drug**	□$_1$ Definitely □$_2$ Most likely □$_3$ Probably □$_4$ Probably not relevant □$_5$ Unlikely to be relevant		
Description of the course of the adverse reaction/event (including symptoms, signs, clinical tests, etc.) and its treatment:			

* AE Degree: Mild: tolerated by the subject, not affecting treatment, not requiring special treatment, without effect on the subject's recovery. Moderate: intolerable to the subject, requiring, withdrawal of the drug or special treatment, with direct effect on the subject's recovery. Severe: life-threatening, lethal or disabling to the subject, requiring immediate withdrawal of the drug or emergency treatment.

Note: For serious adverse events, please fill out the Serious Adverse Event Report Form and report according to the prescribed procedures.

Observing physicians__________________ Date _ _ _ _ year _ _ month _ _ day

Study No. A00908	Center No. |_|_|	Name Initial |_|_|_|_|	Subject No. |_|_|_|	Out-of-time visit

Sticky Place of Inspection Report Forms for Out-of time Visit

Study No. A00908	Center No. |_|_|	Name Initial |_|_|_|_|	Subject No. |_|_|_|	Out-of-time visit

Patient Outcome

Study Completion/Discontinuation

**Date of Completion/Discontinuation**	|_|_|_|_|year|_|_|month|_|_|day
**Did the subject complete what was required by the protocol?**	☑1 Yes<br>☐0 No, please fill in reason for discontinuation
**Reason for discontinuation**	☐1 Investigator's decision to discontinue, reason________<br>☐2 Subject's decision to discontinue, reason___________<br>☐3 Lost to follow-up<br>☐4 Death, reason_____________<br>☐5 Other reason for discontinuation, please specify: ____<br>____

Protocol Deviation

No.	Deviation Description	Start Date	Handling measures	End Date
1		|_|_|_|_|year|_|_|month|_|_|day		|_|_|_|_|year|_|_|month|_|_|day
2		|_|_|_|_|year|_|_|month|_|_|day		|_|_|_|_|year|_|_|month|_|_|day
3		|_|_|_|_|year|_|_|month|_|_|day		|_|_|_|_|year|_|_|month|_|_|day

Review Signature Page

I have reviewed all of the contents in the CRF and have ensured that it is complete and authentic and meets the requirements of the study protocol.

Study leader's signature: _____________ **Date: |_|_|_|_|year|_|_|month|_|_|day**
